# Delivery Modality Affect Neonatal Levels of Inflammation, Stress, and Growth Factors

**DOI:** 10.3389/fped.2021.709765

**Published:** 2021-09-22

**Authors:** Pia Kiilerich, Rikke Cortes, Ulrik Lausten-Thomsen, Nis Borbye-Lorenzen, Solveig Holmgaard, Kristin Skogstrand

**Affiliations:** ^1^Department for Congenital Disorders, Danish Center for Neonatal Screening, Statens Serum Institute, Copenhagen, Denmark; ^2^Neonatal Intensive Care Unit, Copenhagen University Hospital Rigshospitalet, Copenhagen, Denmark

**Keywords:** inflammation, neonatal stress, CODIBINE, in-labor cesarean section, pre-labor cesarean section, labor process, biomarkers, dried blood spot sample

## Abstract

**Introduction:** As part of the study CODIBINE, Correlations and Diagnoses for Biomarkers in New-borns, the main objective of the study was to explore neonatal inflammation, stress, neurodevelopment, and growth factors after in-labor and pre-labor cesarean section compared to vaginal delivery. Increasing evidence has shown that birth delivery mode has an impact on imminent and long-term child health. However, the effect of the timing of cesarean section is insufficiently elucidated. The main objective of the study was to explore the effect of different delivery modes, vaginal delivery compared to cesarean section with or without initiation of labor, on the infants.

**Methods:** We designed a retrospective cohort study, including dried blood spot samples from mature (gestational age ≥ 37) newborns delivered in the years 2009-2011. The newborns were divided into three groups after delivery mode: (1) pre-labor cesarean section (*n* = 714), i.e., cesarean delivery without initiation of labor, (2) in-labor cesarean section (*n* = 655), i.e., cesarean section after initiation of labor, and (3) vaginal delivery (*n* = 5,897). We measured infant levels of inflammatory (IL-18, MCP-1, CRP, sTNF RI), stress (HSP-70), growth (EGF, VEGF-A), and neurotrophic factors (BDNF, NT-3, S100B) 2–4 days after birth.

**Results:** The neonatal levels of inflammatory and stress markers were significantly lower, while the levels of growth factors were higher after pre-labor cesarean section compared to vaginal delivery. The biomarker levels were similar after in-labor cesarean section and vaginal delivery. Removing cases with pre-labor rupture of membranes and artificial rupture of membranes in the calculations did not change the results. The levels of neurotrophic factors were unaffected by delivery form. Males had generally higher levels of inflammation and lower levels of growth and neurotrophic factors. Overall, the levels of inflammatory markers increased, and the growth factors decreased with increasing gestational age.

**Conclusion:** The present study of the biomarker levels after birth suggests that the labor process has an important effect on the fetal immune system and level of stress, regardless if the delivery ends with cesarean section or vaginal birth.

## Introduction

Cesarean section (CS) is a well-known and potentially life-saving surgical procedure. The global rate of deliveries by CS has increased from 12.1% in 2000 to 21.1% of deliveries in 2015 (1). It is estimated that 9–19% of CSs can be justified by medical indications (2), and the World Health Organization currently recommends CS for up to 15% of deliveries (3). Yet, national frequencies vary greatly and range from 0.6% in South Sudan (1) to 55–65% in Brazil (4).

Recently, increasing evidence has shown that birth delivery mode has an impact on child health (5). CS is associated with early complications such as birth asphyxia, respiratory disturbances (6), and soft tissue injury (7), and with neurological and psychological complications such as autism spectrum disorders (8), ADHD (9), psychosis (10), anxiety, depression, and sleep disturbances (11). Notable CS-associated late-term complications for the child includes implications of the immune system: systemic connective tissue disorders, juvenile arthritis (12), inflammatory bowel disease, immune deficiencies, asthma (13), sepsis (14), type 1 diabetes (13), celiac disease (15), and autoimmune diseases (11).

Compared to infants delivered vaginally, a delivery by CS is thought to alter the short-term immune response in newborns by variation of bacterial colonization of the intestinal tract, due to lack of exposure to the vaginal and anal microbiota during delivery (16). Further, the level of fetal stress during pre-labor CS is lower compared to vaginal delivery (VD), as both the initiation of birth and the contractions during VD that may trigger many biological effects are lacking (17). Pre-labor CS, i.e., CS before the onset of labor, are more often associated with several of the abovementioned complications compared with in-labor CS (6, 10, 12, 13, 15), suggesting that important endocrine, physiological, and biochemical processes in the infant are initiated by labor. The idea to induce mild contractions with oxytocin before pre-labor CS has been described to reduce neonatal respiratory morbidity (18).

Most biomarker studies reported regarding CS vs. VD have excluded cases with in-labor CS; thus, the studies cannot explain if the differences found are due to the surgery or the lack of labor.

Accordingly, we aimed to explore the delivery mode's effects on the immune system and brain *per se* of the infants, by measuring inflammatory, stress, growth, and neurotrophic biomarkers in more than 7,000 neonatal dried blood spot samples (DBSS). We carefully selected three biomarkers for inflammation [interleukin-18 (IL-18), monocyte chemotactic protein (MCP-1), C-reactive protein (CRP)], one anti-inflammatory marker [soluble tumor necrosis factor 1 (sTNF RI)], one biomarker for stress [heat shock protein-70 (HSP70)], two growth factors [epidermal growth factor (EGF), vascular endothelial growth factor A (VEGF-A)], and three neurotrophic biomarkers for brain development and/or damage [brain-derived neurotrophic factor (BDNF), neurotrophin-3 (NT-3), and S100 calcium-binding protein B (S100B)]. The inflammatory biomarkers were selected if quantifiable in DBSS and to cover a broad range of the immune system. Classic inflammatory markers like IL-6, TNF, and IL-10 were not included, due to very low concentrations in DBSS.

Finally, we wanted to elucidate potential gender- and gestational age-dependent differences of the biomarker levels.

## Materials and Methods

### Sample Selection

This is the first of a series of papers from the project called CODIBINE, Correlations and Diagnoses for Biomarkers in New-borns, aiming to explore biomarkers in newborns correlated with birth, complications, and diagnoses later in life. The CODIBINE cohort comprises all newborns born with gestational age (GA) < 37 weeks (*n* = 7,946) in Denmark between March 2009 and March 2011 and maturely born controls with GA ≥ 37 weeks (*n* = 7,946) matched by birth hospital and day. The blood samples were drawn from a heel prick as DBSS for routine newborn screening. Samples are shipped by mail and stored at the Danish New-born Screening Biobank, Statens Serum Institut, at −24°C after screening (19). Our cohort is linked with data from the Danish Medical Birth Register (20) using the Danish Civil Registration System (21).

In the present study, we reduced the cohort to only include mature births, with samples drawn 2–4 days after birth, in total 7,266 individuals: 714 delivered by pre-labor CS, i.e., CS before onset of labor, 655 by in-labor CS, i.e., CS after initiation of labor, and 5,897 delivered vaginally. The study was designed as a retrospective cohort study. Table 1 shows the demographic data for the mothers and newborns.

**Table 1:** Maternal and neonatal demographic data.

**Type of birth**	**Pre-labor** **cesarean section**, ***n*****=714**		**In-labor** **cesarean section**, ***n*****=655**		**Vaginal delivery**, ***n*****=5,897**	
**Gender**	**Male**	**Female**	**Male**	**Female**	**Male**	**Female**
Number (% of total *n* = 7,266)	379 (5.2%)	335 (4.6%)	389 (5.4%)	266 (3.7%)	3,132 (43.1%)	2,765 (38.1%)
**Birth weight (gram)**
(95% CI)	3,543	3,326	3,683	3,571	3,613	3,499
	(3,491–3,594)	(3,271–3,381)	(3,629–3,737)	(3,508–3,634)	(3,597–3,630)	(3,482–3,516)
**Maternal BMI**
Mean	25.5	25.4	25.4	25.8	24.0	24.1
(95% CI)	(24.9–26.1)	(24.7–26.1)	(24.8–26.0)	(25.1–26.4)	(23.9–24.2)	(23.9–24.3)
**Maternal age (years)**
Mean	32.6	31.9	30.1	30.8	30.2	30.4
(95% CI)	(32.2–33.1)	(31.4–32.4)	(29.6–30.6)	(30.2–31.4)	(30.0–30.4)	(30.2–30.6)
**Gestational age (weeks, % of** ***n*** **in the delivery group)**
37	52 (7.3%)	50 (7.0%)	28 (4.3%)	17 (2.6%)	155 (2.6%)	138 (2.3%)
38	159 (22.3%)	150 (21.0%)	49 (7.5%)	40 (6.1%)	388 (6.6%)	294 (5.0%)
39	139 (19.5%)	120 (16.8%)	70 (10.7%)	51 (7.8%)	695 (11.8%)	602 (10.2%)
40	21 (2.9%)	7 (1.0%)	91 (13.9%)	65 (9.9%)	1041 (17.7%)	935 (15.9%)
41	6 (0.8%)	6 (0.8%)	99 (15.1%)	64 (9.8%)	661 (11.2%)	637 (10.8%)
42	<5 (<0.7%)	<5 (<0.7%)	52 (7.9%)	29 (4.4%)	192 (3.3%)	159 (2.7%)
**Age at sampling (days, % of** ***n*** **in the delivery group)**
2	270 (37.8%)	234 (32.8%)	184 (25.4%)	132 (18.2%)	1656 (28.1%)	1405 (23.8%)
3	105 (14.7%)	98 (13.7%)	195 (26.9%)	125 (17.3%)	1406 (23.8%)	1307 (22.2%)
4	<5 (<0.7%)	<5 (<0.7%)	10 (1.4%)	9 (1.2%)	70 (1.2%)	53 (0.9%)
**Season variation (% of n in the delivery group)**
Summer (May–Oct)	195 (27.3%)	180 (25.2%)	197 (30.1%)	132 (20.2%)	1589 (26.9%)	1453 (24.6%)
Winter (Nov–April)	184 (25.8%)	155 (21.7%)	192 (29.3%)	134 (20.5%)	1543 (26.2%)	1312 (22.2%)
Anesthetics^*^ **(number, % of** ***n*** **in the delivery group)**
General	6 (0.8%)	7 (1.0%)	25 (3.8%)	11 (1.7%)	18 (0.3%)	32 (0.5%)
Regional	370 (51.8%)	323 (45.2%)	349 (53.3%)	249 (38.0%)	418 (7.1%)	366 (6.2%)
Both	<5 (<0.7%)	5 (0.7%)	15 (2.3%)	6 (0.9%)	5 (0.1%)	6 (0.1%)
PROM (number, % of n in the delivery group)	<5 (<0.7%)	<5 (<0.7%)	48 (7.3%)	36 (6.0%)	221 (3.7%)	188 (3.2%)
AROM (number, % of n in the delivery group)	<5 (<0.7%)	<5 (<0.7%)	73 (18.8%)	49 (18.4%)	506 (16.2%)	415 (15.0%)

*PPROM, pre-labor rupture of the membranes; AROM, artificial rupture of membranes*.

* *We were unable to distinguish between anesthetics given during and after delivery. Thus, some may be given after delivery due to immediate acute surgery*.

Exclusion criteria were active dissent to participation in scientific studies (as registered in the Danish national register for use of biological tissue, “Vævsanvendelsesregisteret”), lack of or insufficient sample material, incomplete civil registration number, and non-participation in the National New-born Screening Program.

The study was approved by the Danish Ethical Committee (VEK), Project-ID H-6-2014-078 and H-6-2014-079. According to Danish law, dispensation can be given for not getting written informed consent when using material from a biobank, when individuals are not contacted or affected in any way (komitélovens § 10, stk. 1). Dispensation was given for this study.

### Sample Analysis

The samples were analyzed using an in-house-developed multiplex assay based on Meso Scale Discovery (MSD) technology, targeting IL-18, MCP-1, CRP, sTNF RI, HSP70, EGF, VEGF-A, BDNF, NT-3, and S100B.

All antibodies were purchased from R&D Systems, with the exception of anti-S100B, which was purchased from Sigma-Aldrich (capture) and DAKO (detection), respectively. The capture antibodies were purchased biotinylated, except for anti-S100B that was in-house purified using a protein A column, and biotinylated using EZ-Link Sulfo-NHS-LC-Biotin (Thermo Fisher) as per the manufacturer's instructions. The biotinylated antibodies were diluted to the concentration 0.1 μg/ml for CRP and 10 μg/ml for the other antibodies, bound to different linkers 1–10 (MSD) according to the manufacturer's instructions, and finally added to each plate well (50 μl/well, U-PLEX plates, MSD) and incubated at room temperature (RT) for 1 h. After washing with washing buffer (PBS containing 0.05% Tween 20), the plates were stored at 4°C until use. Calibrators and controls were prepared by recombinant antigens diluted in diluent 7 (MSD). All detection antibodies were sulfo-tagged using MSD Gold Sulfo-tag NHS-Ester (MSD) as per the manufacturer's instructions and were mixed together to a final concentration of 0.1 μg/ml of each antibody.

The DBSS were punched as 2 × 3.2-mm disks in microtiter wells using a DBS Puncher^®^ instrument (Perkin Elmer), and 130 μl extraction buffer [PBS containing protease inhibitor, as earlier described (22)] was added to each well, sealed, and extracted for 1 h at RT on a shaker set at 450 rpm. After extraction, 50 μl extract was aspirated from each well and transferred to the precoated plates with a pipetting robot (Biomek 4000, Ramcon) together with calibrators and controls. The plates were sealed and incubated on a shaker set at 600 rpm for 2 h at RT. After washing, detection antibodies were added to each well, followed by a 2-h incubation at 600 rpm at RT.

Finally, the plates were washed, added 2x Read Buffer T (MSD), and immediately read at the QuickPlex SQ 120 reader (MSD).

Concentrations of the biomarkers were calculated from the calibration curves with a four-parameter logistic fit using Discovery Workbench 4.0 software (MSD).

Analytical characteristics are shown in Table 2.

**Table 2:** Analytical characteristics.

	**Working range pg/ml**		**Intra**	**Inter**

**Analyte**	**Low**	**High**	**Assay CV%**	
BDNF	61.5	40,000	19.8	28.7
CRP	43.6	10,000,000	14.8	47.3
EGF	2.5	5,000	6.9	12.2
HSP70	246.1	5,000,000	6.9	17.8
IL-18	0.2	5,000	7.5	13.8
MCP-1	4.4	50,000	9.9	22.4
NT-3	2.0	5,000	8.4	16.9
S100B	28.8	50,000	15.2	24.6
sTNF-R1	16.9	100,000	6.7	11.9
VEGF	3.0	5,000	6.1	9.5

*The lowest concentrations in the working range were calculated as the concentrations 2.5 standard deviations above the lowest points on the calibration curves, and the highest points were set as the highest calibration points. Assay CV% was calculated from controls present on all the sample plates, in total 211 plates. Intra-assay CV% was calculated as the mean CV% calculated from controls first and last on each plate, while inter-assay CV% was calculated as the variation between controls on all plates. Values measured below or above the working ranges were set to the lowest or highest measurable concentrations*.

### Statistics

For each of the 10 biomarkers, the data were log-transformed using the natural logarithm in order to obtain approximately normal distribution. The mode of delivery was stratified into three groups; VD, pre-labor CS, and in-labor CS, as classified by the obstetrician at the time of birth. Analysis of covariance (ANCOVA) was used to test for the overall difference between birth types for each biomarker, controlling for GA. The pairwise comparisons of each birth type per gestational week were calculated using contrasts from estimated marginal means from the respective ANCOVA model using the emmeans R package (23). The *p*-values for the pairwise comparisons were adjusted with the Holms method (24) for multiple testing. ANCOVA was further used to test for the overall difference in birth types for each gender and to calculate pairwise comparisons between the genders for each birth type, using contrasts and adjusting with the Holms method for multiple testing. The same method was used to test the overall difference between GA and calculate pairwise comparisons using contrasts, adjusted with the Holms method for multiple testing.

Neonatal age at sampling, maternal age, and the BMI of the mother all contributed to the variation of multiple biomarkers and were associated with at least one of the tested variables (Supplementary Material S2.2–S2.4). Thus, these factors were controlled for in the models.

The birth weight and GA were lower in the pre-labor CS group compared to the other groups. Birth weight was further highly correlated with GA (Pearson correlation coefficient = 0.4281, Supplementary Material S2.5). The variation in intrauterine growth was not considered as a cause of variation in the biomarkers, and birth weight was thus excluded in the final calculations.

There were no statistical significant difference between the numbers in each delivery group between summer and winter; numbers are shown in Table 1.

There was a significant difference in the use of anesthetics between pre-labor and in-labor CS, where general anesthetics was more frequent used in in-labor CS (Supplementary Material S2.6). The statistical tests were rerun excluding cases with general anesthetics. No general difference in the results was found (Supplementary Material S3, S3A).

The analyses were rerun excluding the cases with pre-labor rupture of the membranes (PROM) and artificial rupture of membranes (AROM) as well. No general difference in the result was found (Supplementary Material S4, S4A). Only 0.8% of the infants were registered with an infection the first 4 days after birth; thus, this was not adjusted for in the statistics.

All statistical analyses and figures were performed using the dplyr (25), emmeans (23), car (26), and ggplot2 (27) packages in the R software version 4.0.2 (all statistical codes are shown in Supplementary Material S1).

## Results

**Figure 1:**
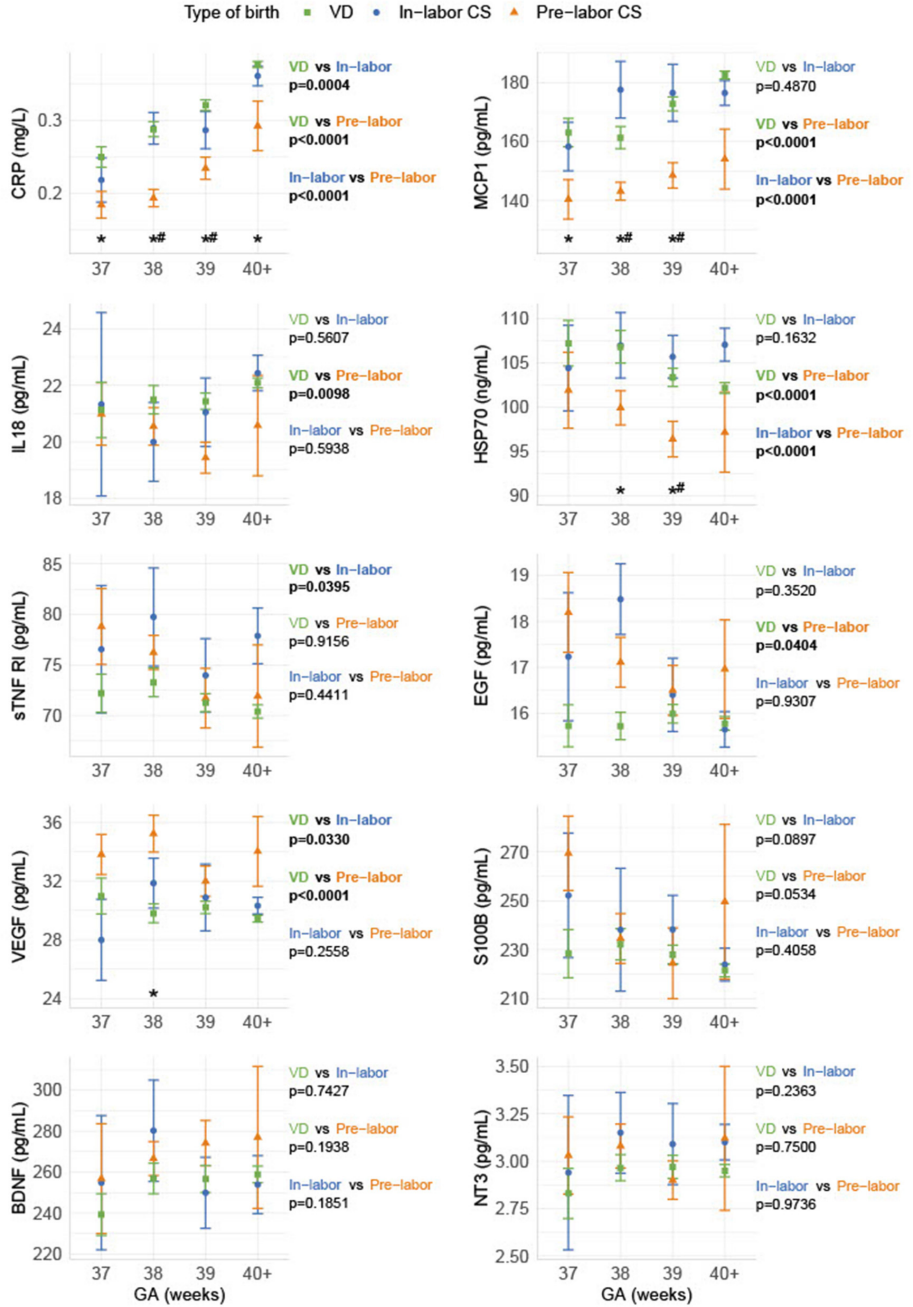
Biomarker levels depending on gestational age and delivery form. The biomarkers are shown as sample median with a bootstrapped estimate of the standard error of the median, divided into GA and pre-labor CS, in-labor CS, and VD, respectively. The *p*-values shown are calculated as an overall difference for the pairs of different birth types. *Statistical significance between VD and pre-labor CS for the specific GA. #Statistical significance between pre-labor CS and in-labor CS for the specific GA.

Infants born by pre-labor CS had significantly lower blood contents of the inflammatory markers (CRP, MCP-1, IL-18) and the stress marker HSP70 and significantly increased levels of the growth factors (VEGF and EGF) compared to infants born by VD. Comparing pre-labor CS with in-labor CS showed a similar pattern: CRP, MCP-1, and HSP70 were significantly decreased in infants born by pre-labor CS compared to infants born by in-labor CS. When comparing in-labor CS to VD, only CRP, VEGF, and sTNF RI were significantly different. We found no delivery form depending on differences in neonatal levels of the neurotrophic factors (S100B, BDNF, and NT-3) (shown in Figure 1).

**Figure 2:**
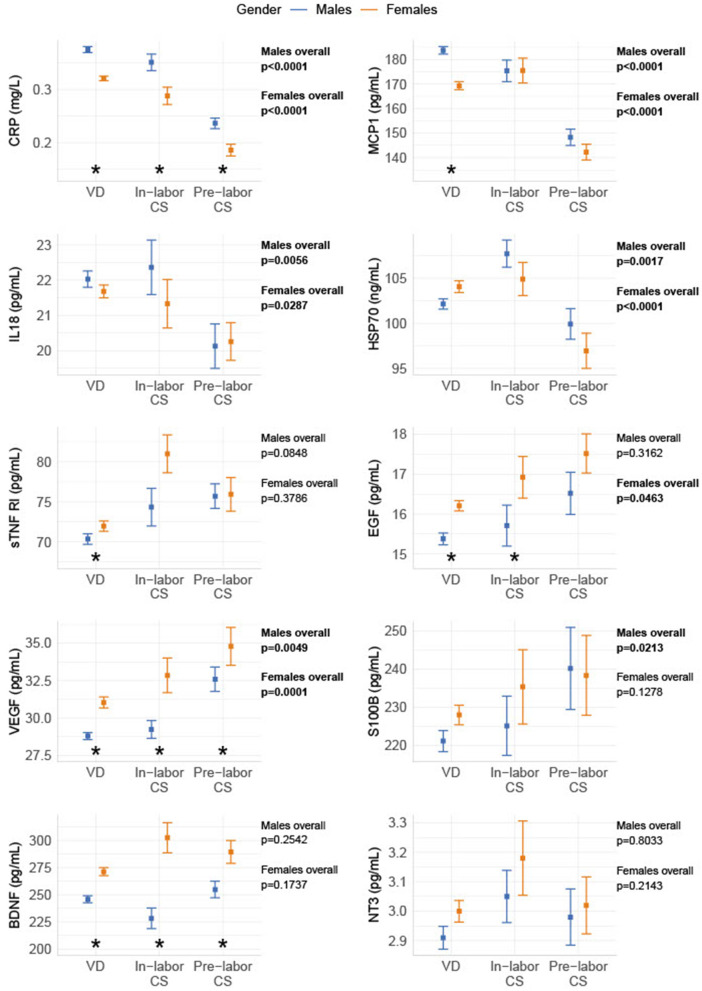
Gender differences in biomarker levels depending on birth form. The biomarkers are divided into gender and grouped into pre-labor CS, in-labor CS, and VD. The data are shown as sample median with a bootstrapped estimate of the standard error of the median. The *p*-values shown are calculated as an overall difference in birth types for males and females, respectively. *Statistical significant difference between genders for the specific delivery form.

Most biomarkers were different between genders: males had significantly lower levels than females of the anti-inflammatory marker sTNF RI, the growth factors (EGF and VEGF), and the neurotrophic factor BDNF. The inflammatory markers CRP and MCP-1 were higher in males compared to females. Delivery form had an overall effect on S100B in males, but not in females, while EGF had an overall effect in females, but not in males. For all other biomarkers, the observed overall effects between delivery forms were similar between genders. The gender differences in biomarkers were generally more significant after VD (CRP, MCP-1, sTNF RI, EGF, BDNF, VEGF), than after in-labor CS (CRP, BDNF, VEGF, EGF) and pre-labor CS (CRP, BDNF, VEGF) (shown in Figure 2).

**Figure 3:**
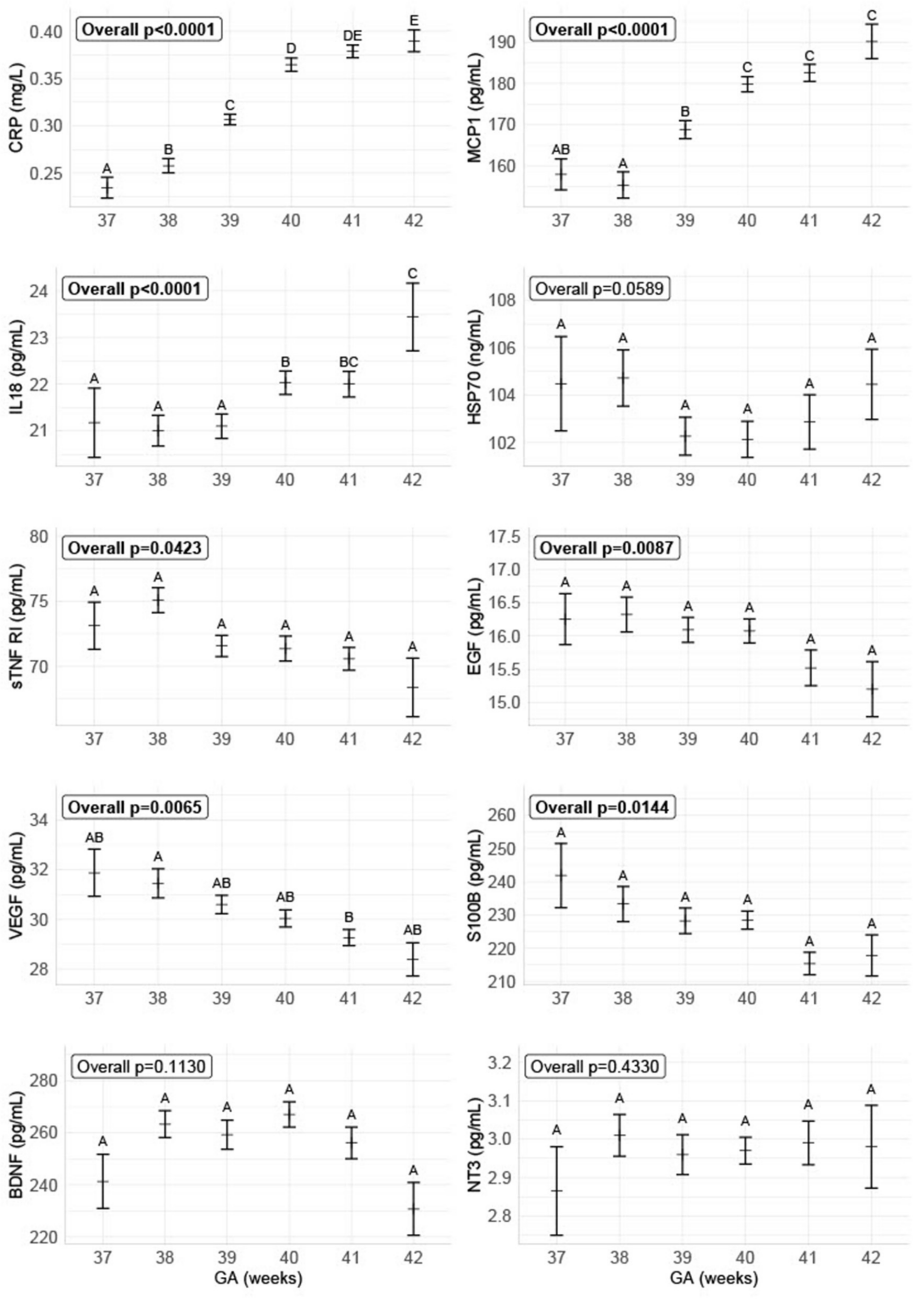
Biomarker levels depending on gestational age on delivery. The figure shows the concentrations of the different biomarkers divided into GA, combined for all delivery forms. The data are plotted as the group sample median with a bootstrapped estimate of the standard error of the median as the whiskers. The biomarker concentrations for the different GA with no letters in common are significantly different with α = 0.05.

We found an overall increase from GA 37-42 of the inflammatory markers CRP, IL-18, and MCP-1 and a decrease of the anti-inflammatory marker sTNF RI, the growth factors EGF and VEGF, and the neurotrophic factor S100B. There was no overall significant difference for the neurotrophic factors BDNF and NT-3, and the stress marker HSP70 (shown in Figure 3).

As expected, anthropometrics varied between the groups. There was a significant difference in infants' birth weight between delivery forms, where the heaviest infants are born by in-labor CS and the lightest by pre-labor CS. Further, the GA were slightly lower for infants born by pre-labor CS compared to the other groups (Table 1). Variables that contributed to biomarker variations were included in the statistical models; please see “Statistics” for details. There were significantly more cases with PROM and AROM before in-labor CS and VD, compared to pre-labor CS. Removing all cases with pre-labor rupture of membranes (PROM) and artificial rupture of membranes (AROM) in the calculations made small changes, but did not change the general results; please see the Supplementary Material S4A. The registration of use of anesthesia was not complete, especially regarding the use of anesthesia during CS. The general recommendation in Denmark is to use regional anesthesia (28); thus, we assume that this was used unless otherwise registered. Removing births with general anesthesia made very small changes; please see the Supplementary Material S3A. As these changes were minimal, we decided to use the results that included as many individuals as possible.

## Discussion

In this study, the neonatal levels of inflammatory and stress markers were generally lower, and the levels of growth factors were higher after pre-labor CS compared to those after VD or in-labor CS. The differences in biomarkers could not be explained by the higher incidence of PROM or AROM before in-labor CS and VD. Accordingly, the data suggest that the labor process has an important physiological effect on the fetal immune system and level of stress, regardless if the delivery ends with cesarean section or vaginal birth.

Decreased levels of inflammatory markers in cord blood and neonatal serum after CS compared with VD has been described before (29), but most previous studies did not stratify for the type of CS (pre-labor or in-labor). A few studies regarding CRP have been described, which found similar differences in newborn levels depending on birth form as observed in the current study (30). In a study among teenagers, whose mothers had entered the active phase of labor before CS, spontaneous and toll-like receptor-stimulated cytokine release was increased, compared to controls born by pre-labor CS (31). This concurs with another study showing that the risk for early childhood infections is higher in children born by pre-labor CS compared to children born by in-labor CS or VD (32). One of the common explanations for the immunological differences after CS and VD has been microbial transmission from mother to child, either by transmission during vaginal birth or by microbial invasion of the amniotic cavity after PROM (32). However, in our study, the exclusion of all cases with PROM and AROM did not make any difference in the significance of the biomarker levels. Following national guidelines, prophylactic antibiotics are given during or after both pre- and in-labor CS in Denmark (33), and the hospital stays afterwards are the same (in contrast to after VD where the woman and infant often leave the hospital a few hours after birth). The explanation for the observed differences in our study is thus more likely to be the influences of stress hormones and/or the physical pressure from the labor process. During VD, cortisol and other stress hormones increase. Elevated cortisol at birth is a known indicator of hypothalamic–pituitary–adrenal axis activation, which is important for the regulation of stress and many other body processes (34). The observed sustained inflammatory response 2–4 days after birth in our study after in-labor CS or VD may thus be protective for the fetus later in life and could possibly even explain some of the increased risk for autoimmune and inflammatory disorders after pre-labor CS.

We found reduced levels of HSP70 after pre-labor CS compared to in-labor CS and VD. The intracellular inducible HSP70 is one of the major HSPs involved in numerous cellular functions, such as cytoprotection, anti-apoptosis, and immune regulatory effects (35). Increased temperature, exposure to oxidative stress, such as hypoxia, viral infection, and ischemia–reperfusion injury can induce the expression (35). The fact that HSP70 had similar infant levels after in-labor CS and VD may indicate that the stress effect is the same provided the labor has been initiated, regardless if the delivery ends with CS or VD.

Animal studies have shown that mouse brains have increased contents of norepinephrine, dopamine, serotonin, and metabolites of dopamine and serotonin after vaginal deliveries compared to mice delivered by CS. The turnover ratios of the neurotransmitters were also higher in the mouse brains after vaginal delivery, and the later adult mice showed different behavioral patterns (36). We have recently found significantly lower neonatal levels of BDNF in newborns later diagnosed with autism spectrum disorders (37). In the current study, we did not see any correlation with the neurotrophic markers BDNF, NT-3, and S100B, and the delivery form. This might indicate that neurodevelopment is not dependent on delivery form.

We found increased levels of VEGF after pre-labor and in-labor CS compared to VD and increased levels of EGF after pre-labor CS compared to VD. VEGF is a growth factor that stimulates vasculogenesis and angiogenesis after stress, and it is an essential factor for placental development (38). VEGF is expressed at sites of injury and inhibits the activity of nitric oxide synthase, preventing inflammation (39), and it is present at high levels in the central nervous system (40). EGF is widely expressed in the body and plays a fundamental role in embryonic development, stem cell and tissue regeneration, and ion transport (41). VEGF is a potent stimulator of angiogenesis in asthma (42), and both VEGF and EGF are stimulators of mucins in the respiratory tract, whose concentrations have been reported as positively correlated with asthma disease severity (43). We are not aware of any other studies regarding delivery forms and growth factors. The increased levels after pre-labor CS could indicate that the mechanisms for the increased risk for asthma have been initiated already 2–4 days after birth, but this needs to be more thoroughly investigated in further studies using asthma as an outcome.

We found significantly different levels for most biomarkers between genders. Higher concentrations of CRP in neonatal males compared to females have been described before (44). This may be due to hormonal differences, as estradiol (which is highest in females) decreases the production of CRP (45). The higher levels of BDNF in females can also be explained by the higher levels of estradiol, which increases the gene expression of BDNF (46). Although these early-life differences may simply be an epiphenomenon, it might be the precursors to later-in-life higher frequencies of different disorders in males, but this needs further investigation. Circumcision of baby males are rarely performed in Denmark (47); thus, this cannot explain any of the gender biomarker differences.

The overall increasing levels of inflammation biomarkers from GA 37 to 42 concur with a study showing that the leukocyte count increases with GA up to week 40 (48). The growth factors and S100B decreased for each week of GA, while the neurotrophic and stress factors did not depend on GA. We did not see any differences in the neurotrophic markers, which could explain cognitive impairment when delivering at lower GA, but the higher levels of growth factors and S100B could indicate a less mature body and brain.

The strengths of this study are the study size and the unbiased and random selection of the participants from all of Denmark, meaning that there was no selection for social status, income, race, etc. All samples were handled in the same way and were analyzed in the same laboratory; thus, the analytic variance was minimized. Drawing parallels on the later outcome based on biomarker analysis in dry blood after prolonged storage may not be appropriate, but it may point out areas for more thorough investigations. We were not able to account for lifestyle factors associated with maternal request for CS or factors increasing the risk of CS. Further, we did not have any information regarding length of labor.

## Conclusion

The present results provide further evidence that the labor process is an important and necessary part of delivery for the infants' immune system and also adds to the growing body of evidence, suggesting that an unnecessary “overproduction” of CS leads to not only maternal and socioeconomic but certainly also neonatal consequences. The long-term outcome, after pre-labor and in-labor CS, on the developing immune system in the children in this study remains however to be investigated.

## Data Availability Statement

The datasets presented in this article are not readily available because all statistical codes used, and all calculated results are available in Supplements. According to the European General Data Protection Regulation (GDPR), we are not allowed to publish data on individual levels. Thus the raw data cannot be publicly available. Requests to access the datasets should be directed to Kristin Skogstrand, ksk@ssi.dk.

## Ethics Statement

The studies involving human participants were reviewed and approved by Danish Ethical Committee (VEK), Project-ID H6-2014-078 and H-6-2014-079. Written informed consent from the participants' legal guardian was not required to participate in this study in accordance with the national legislation and the institutional requirements.

## Author Contributions

PK, RC, UL-T, NB-L, and SH analyzing and writing up the work. KS conception, planning, carrying out, analyzing, and writing up the work. All authors contributed to the article and approved the submitted version.

## Funding

This study was supported by Læge Sofus Carl Emil Friis og hustru Olga Doris Friis' legat and Fonden til Lægevidenskabens Fremme. The sponsors were not involved in any part of the study.

## Conflict of Interest

The authors declare that the research was conducted in the absence of any commercial or financial relationships that could be construed as a potential conflict of interest.

## Publisher's Note

All claims expressed in this article are solely those of the authors and do not necessarily represent those of their affiliated organizations, or those of the publisher, the editors and the reviewers. Any product that may be evaluated in this article, or claim that may be made by its manufacturer, is not guaranteed or endorsed by the publisher.

## Abbreviations

ADHD: attention-deficit hyperactivity disorder
ANCOVA: analysis of covariance
AROM: artificial rupture of membranes
BDNF: brain-derived neurotrophic factor
CODIBINE: Correlations and Diagnoses for Biomarkers in New-borns
CRP: C-reactive protein
CS: Cesarean section
DBSS: dried blood spot samples
EGF: epidermal growth factor
GA: gestational age
HSP70: heat shock protein-70
IL-18: interleukin-18
MCP-1: monocyte chemotactic protein
MSD: mesoscale diagnostics
NT-3: neurotrophin-3
PROM: pre-labor rupture of the membranes
S100B: S100 calcium-binding protein B
sTNF RI: soluble tumor necrosis factor 1
VD: vaginal delivery
VEGF-A: vascular endothelial growth factor A.
