# Supplementary material for: Delivery Modality Affect Neonatal Levels of Inflammation, Stress, and Growth Factors
Source: Front Pediatr. 2021 Sep 22;9:709765. doi: 10.3389/fped.2021.709765 (PMC8492985; doi:10.3389/fped.2021.709765)
Supplement: Supplementary file 1 [file Data_Sheet_1.zip › S1_Code_for_analysis.docx]

- S1.1 Loading packages and data
  - S1.1.1 CS is redefines in an understandable way and added to the other dataset
    - Renaming variables with information that are understandable instead of codes
  - S1.1.2 Filtering so we only have the children born on time
  - S1.1.3 Biomakers are log transformed
  - S1.1.4 Functions are defined
- S1.2 Testing birth type
- S1.3 Testing gender
- S1.4 Testing the GA for all biomarkers

S1: Code for data analysis for ‘The effect of delivery mode, divided into vaginal delivery and caesarean section with or without initiation of labour, on neonatal inflammarion, stress, neurotrophic and growth factors: a retrospective cohort study’

The variables maternal age, BMI_MODER and age at sampling are all explaining some variation for some of the biomarkers, therefore all analysis of variance are made with ANCOVA (analysis of co-variance) controlling for these variables. This is done to make sure the effects seen are because of the variables tested, not one of the other variables (ruling out confounders). Birth weight are not included, since is very correlated with GA, but we expect the effects seen to be because of GA, not weight.

S1.1 Loading packages and data

S1.1.1 CS is redefines in an understandable way and added to the other dataset

MFR_kejsersnit <- rbind(MFR_KEJSERSNIT2009, MFR_KEJSERSNIT2010, MFR_KEJSERSNIT2011)

MFR_kejsersnit <- MFR_kejsersnit %>%

filter(SKSKODE %**in**% c("KMCA10B", "KMCA10E", "KMCA10A","KMCA10D" )) %>%

mutate(Birth_type = ifelse(SKSKODE == "KMCA10B", "Pre-labor",

ifelse(SKSKODE== "KMCA10E", "In-labor", "Slet"))) %>%

arrange(FK_MFR, Birth_type) %>%

distinct(FK_MFR, .keep_all=TRUE)

MFR2 <- MFR_kejsersnit %>%

right_join(upload_MFR, by="FK_MFR")

Renaming variables with information that are understandable instead of codes

MFR <- MFR2 %>%

mutate(tvilling = ifelse(str_detect(FLERFOLDSGRAVIDITET, "DO300"), "Ja", "Nej")) %>%

mutate(flerfold = ifelse(str_detect(FLERFOLDSGRAVIDITET, "DO30"), "Ja", "Nej")) %>%

mutate(PPROM = ifelse(PPROM=="DO422", "Ja", "Nej")) %>%

mutate(sepsis = ifelse(str_detect(SEPSIS_BARN, "DP36"), "Ja", "Nej")) %>%

replace_na(list(tvilling="Nej")) %>%

replace_na(list(flerfold="Nej")) %>%

replace_na(list(PPROM="Nej")) %>%

replace_na(list(sepsis="Nej")) %>%

replace_na(list(Birth_type="Vaginal")) %>%

mutate(CRP = CRP*1e-06) %>%

mutate(gestation_uge_f = as.factor(gestation_uge)) %>%

filter(gestation_uge > 23 & gestation_uge < 43) %>%

filter(Birth_type != "Slet") %>%

data.table

S1.1.2 Filtering so we only have the children born on time

MFR_3742 <- MFR %>%

filter(gestation_uge > 36 & gestation_uge < 43) %>%

data.table

MFR_3742 <- MFR_3742 %>%

mutate(Gender = ifelse(KOEN_BARN=="M", "Boys", "Girls")) %>%

data.table

rm(MFR_kejsersnit, MFR_KEJSERSNIT2009, MFR_KEJSERSNIT2010, MFR_KEJSERSNIT2011, MFR2, upload_MFR)

S1.1.3 Biomakers are log transformed

MFR_3742$logCRP <- log(MFR_3742$CRP)

MFR_3742$logIL18 <- log(MFR_3742$IL18)

MFR_3742$logMCP1 <- log(MFR_3742$MCP1)

MFR_3742$logHSP70 <- log(MFR_3742$HSP70)

MFR_3742$logSTNF_RI <- log(MFR_3742$STNF_RI)

MFR_3742$logEGF <- log(MFR_3742$EGF)

MFR_3742$logBDNF <- log(MFR_3742$BDNF)

MFR_3742$logNT3 <- log(MFR_3742$NT3)

MFR_3742$logS100B <- log(MFR_3742$S100B)

MFR_3742$logVEGF <- log(MFR_3742$VEGF)

MFR_3742$gestation_uge_f <- as.factor(MFR_3742$gestation_uge)

MFR_3740 <- MFR_3742 %>%

mutate(gest = ifelse(gestation_uge == 37, "37",

ifelse(gestation_uge == 38, "38",

ifelse(gestation_uge == 39, "39", "40+"))))

S1.1.4 Functions are defined

*# Testing the effect of the GA on the biomarkers*

bio_gest <- **function**(data, bio){

fit <- aov(log(bio) ~ AGE_AT_COLLECTION + ALDER_MODER + BMI_MODER + gestation_uge_f, data=data)

sf <- Anova(fit, type="III")

sf_lm<- summary.lm(fit)

test <- summary(pairs(emmeans(fit, ~gestation_uge_f)), adjust="holm")

**return**(list(sf, sf_lm$r.squared, test))

}

*#Testing the effect of birth type on the biomarkers*

gest_type <- **function**(data=MFR_3740, bio){

MFR_3740_2 <- data %>%

filter(Birth_type != "Slet") %>%

data.table

m_under <- MFR_3740_2 %>%

filter(Birth_type != "In-labor") %>%

data.table

m_planlagt <- MFR_3740_2 %>%

filter(Birth_type != "Pre-labor") %>%

data.table

m_vaginalt <- MFR_3740_2 %>%

filter(Birth_type != "Vaginal") %>%

data.table

vag_in <- Anova(aov(m_planlagt[[bio]] ~ AGE_AT_COLLECTION + ALDER_MODER + BMI_MODER + gest + Birth_type, data=m_planlagt), type="III")

vag_pre <- Anova(aov(m_under[[bio]] ~ AGE_AT_COLLECTION + ALDER_MODER + BMI_MODER + gest + Birth_type, data=m_under), type="III")

in_pre <- Anova(aov(m_vaginalt[[bio]] ~ AGE_AT_COLLECTION + ALDER_MODER + BMI_MODER + gest + Birth_type, data=m_vaginalt), type="III")

fit <- aov(MFR_3740_2[[bio]] ~ AGE_AT_COLLECTION + ALDER_MODER + BMI_MODER + Birth_type + gest + Birth_type:gest, data=MFR_3740_2)

test <- summary(pairs(emmeans(fit, ~ Birth_type|gest)),by=NULL, adjust="holm")

**return**(list("Vaginal-inlabor"=vag_in, "Vaginal-prelabor"=vag_pre,"Inlabor-prelabor"=in_pre,"Pairwise test"=test))

}

*#Testing the effect of gender on the biomarkers*

koen_type <- **function**(data=MFR_3740, bio){

MFR_3740_2 <- data %>%

filter(Birth_type != "Slet") %>%

data.table

boys <- MFR_3740_2 %>%

filter(Gender == "Boys") %>%

data.table

girls <- MFR_3740_2 %>%

filter(Gender == "Girls") %>%

data.table

b<-Anova(aov(boys[[bio]] ~ AGE_AT_COLLECTION + ALDER_MODER + BMI_MODER + Birth_type, data=boys), type="III")

g<-Anova(aov(girls[[bio]] ~ AGE_AT_COLLECTION + ALDER_MODER + BMI_MODER + Birth_type, data=girls), type="III")

fit <- aov(MFR_3740_2[[bio]] ~ AGE_AT_COLLECTION + ALDER_MODER + BMI_MODER + Birth_type + KOEN_BARN + Birth_type:KOEN_BARN, data=MFR_3740_2)

test <- summary(pairs(emmeans(fit, ~ KOEN_BARN|Birth_type)), by=NULL, adjust="holm")

**return**(list("Boys"=b, "Girls"=g,"Pairwise test"=test))

}

S1.2 Testing birth type

Figure 1 i paper

cleaning up the data and splitting it up

Testing Here an ancova is made for each biomaker for each of the pairs of birthtypes. This is further corrected for GA

gest_type(bio="logCRP")

## $`Vaginal-inlabor`

## Anova Table (Type III tests)

##

## Response: m_planlagt[[bio]]

## Sum Sq Df F value Pr(>F)

## (Intercept) 76.9 1 144.5491 < 2.2e-16 ***

## AGE_AT_COLLECTION 17.0 1 32.0200 1.593e-08 ***

## ALDER_MODER 4.8 1 8.9605 0.0027696 **

## BMI_MODER 0.9 1 1.6261 0.2022923

## gest 110.4 3 69.1831 < 2.2e-16 ***

## Birth_type 6.7 1 12.6238 0.0003837 ***

## Residuals 3343.2 6284

## ---

## Signif. codes: 0 '***' 0.001 '**' 0.01 '*' 0.05 '.' 0.1 ' ' 1

##

## $`Vaginal-prelabor`

## Anova Table (Type III tests)

##

## Response: m_under[[bio]]

## Sum Sq Df F value Pr(>F)

## (Intercept) 112.5 1 209.5305 < 2.2e-16 ***

## AGE_AT_COLLECTION 19.1 1 35.6321 2.512e-09 ***

## ALDER_MODER 4.4 1 8.1966 0.004211 **

## BMI_MODER 0.7 1 1.3369 0.247624

## gest 99.9 3 62.0491 < 2.2e-16 ***

## Birth_type 67.3 1 125.2858 < 2.2e-16 ***

## Residuals 3403.3 6339

## ---

## Signif. codes: 0 '***' 0.001 '**' 0.01 '*' 0.05 '.' 0.1 ' ' 1

##

## $`Inlabor-prelabor`

## Anova Table (Type III tests)

##

## Response: m_vaginalt[[bio]]

## Sum Sq Df F value Pr(>F)

## (Intercept) 29.07 1 49.7921 2.757e-12 ***

## AGE_AT_COLLECTION 0.56 1 0.9554 0.3285

## ALDER_MODER 0.03 1 0.0599 0.8066

## BMI_MODER 0.77 1 1.3243 0.2500

## gest 24.71 3 14.1080 4.707e-09 ***

## Birth_type 10.57 1 18.1127 2.230e-05 ***

## Residuals 767.70 1315

## ---

## Signif. codes: 0 '***' 0.001 '**' 0.01 '*' 0.05 '.' 0.1 ' ' 1

##

## $`Pairwise test`

## contrast gest estimate SE df t.ratio p.value

## (In-labor) - (Pre-labor) 37 0.0852 0.1334 6966 0.639 0.5228

## (In-labor) - Vaginal 37 -0.2453 0.1192 6966 -2.059 0.1768

## (Pre-labor) - Vaginal 37 -0.3306 0.0866 6966 -3.819 0.0014

## (In-labor) - (Pre-labor) 38 0.2650 0.0896 6966 2.958 0.0217

## (In-labor) - Vaginal 38 -0.1140 0.0838 6966 -1.359 0.4093

## (Pre-labor) - Vaginal 38 -0.3790 0.0516 6966 -7.341 <.0001

## (In-labor) - (Pre-labor) 39 0.2550 0.0819 6966 3.114 0.0167

## (In-labor) - Vaginal 39 -0.1051 0.0705 6966 -1.489 0.4093

## (Pre-labor) - Vaginal 39 -0.3601 0.0510 6966 -7.055 <.0001

## (In-labor) - (Pre-labor) 40+ 0.2544 0.1209 6966 2.105 0.1768

## (In-labor) - Vaginal 40+ -0.0941 0.0395 6966 -2.381 0.1037

## (Pre-labor) - Vaginal 40+ -0.3485 0.1156 6966 -3.015 0.0206

##

## P value adjustment: holm method for 12 tests

gest_type(bio="logMCP1")

## $`Vaginal-inlabor`

## Anova Table (Type III tests)

##

## Response: m_planlagt[[bio]]

## Sum Sq Df F value Pr(>F)

## (Intercept) 1945.23 1 10491.8430 < 2.2e-16 ***

## AGE_AT_COLLECTION 52.92 1 285.4247 < 2.2e-16 ***

## ALDER_MODER 0.53 1 2.8527 0.09127 .

## BMI_MODER 0.03 1 0.1400 0.70834

## gest 10.30 3 18.5188 5.877e-12 ***

## Birth_type 0.09 1 0.4832 0.48701

## Residuals 1165.08 6284

## ---

## Signif. codes: 0 '***' 0.001 '**' 0.01 '*' 0.05 '.' 0.1 ' ' 1

##

## $`Vaginal-prelabor`

## Anova Table (Type III tests)

##

## Response: m_under[[bio]]

## Sum Sq Df F value Pr(>F)

## (Intercept) 1896.17 1 10407.940 < 2.2e-16 ***

## AGE_AT_COLLECTION 50.31 1 276.141 < 2.2e-16 ***

## ALDER_MODER 0.43 1 2.336 0.1265

## BMI_MODER 0.08 1 0.441 0.5067

## gest 10.85 3 19.859 8.283e-13 ***

## Birth_type 13.63 1 74.800 < 2.2e-16 ***

## Residuals 1154.87 6339

## ---

## Signif. codes: 0 '***' 0.001 '**' 0.01 '*' 0.05 '.' 0.1 ' ' 1

##

## $`Inlabor-prelabor`

## Anova Table (Type III tests)

##

## Response: m_vaginalt[[bio]]

## Sum Sq Df F value Pr(>F)

## (Intercept) 377.22 1 2243.6930 < 2.2e-16 ***

## AGE_AT_COLLECTION 4.25 1 25.2897 5.615e-07 ***

## ALDER_MODER 0.11 1 0.6678 0.41397

## BMI_MODER 0.03 1 0.1498 0.69875

## gest 1.22 3 2.4107 0.06536 .

## Birth_type 5.10 1 30.3321 4.374e-08 ***

## Residuals 221.08 1315

## ---

## Signif. codes: 0 '***' 0.001 '**' 0.01 '*' 0.05 '.' 0.1 ' ' 1

##

## $`Pairwise test`

## contrast gest estimate SE df t.ratio p.value

## (In-labor) - (Pre-labor) 37 0.0969 0.0776 6966 1.249 1.0000

## (In-labor) - Vaginal 37 -0.0529 0.0693 6966 -0.763 1.0000

## (Pre-labor) - Vaginal 37 -0.1498 0.0503 6966 -2.975 0.0235

## (In-labor) - (Pre-labor) 38 0.1931 0.0521 6966 3.706 0.0019

## (In-labor) - Vaginal 38 0.0271 0.0488 6966 0.556 1.0000

## (Pre-labor) - Vaginal 38 -0.1660 0.0300 6966 -5.528 <.0001

## (In-labor) - (Pre-labor) 39 0.2033 0.0476 6966 4.269 0.0002

## (In-labor) - Vaginal 39 0.0295 0.0410 6966 0.719 1.0000

## (Pre-labor) - Vaginal 39 -0.1739 0.0297 6966 -5.857 <.0001

## (In-labor) - (Pre-labor) 40+ 0.0902 0.0703 6966 1.283 1.0000

## (In-labor) - Vaginal 40+ -0.0306 0.0230 6966 -1.329 1.0000

## (Pre-labor) - Vaginal 40+ -0.1207 0.0672 6966 -1.797 0.5070

##

## P value adjustment: holm method for 12 tests

gest_type(bio="logIL18")

## $`Vaginal-inlabor`

## Anova Table (Type III tests)

##

## Response: m_planlagt[[bio]]

## Sum Sq Df F value Pr(>F)

## (Intercept) 567.34 1 3154.6674 < 2.2e-16 ***

## AGE_AT_COLLECTION 0.12 1 0.6485 0.4206730

## ALDER_MODER 0.00 1 0.0042 0.9481658

## BMI_MODER 0.48 1 2.6575 0.1031142

## gest 3.52 3 6.5224 0.0002115 ***

## Birth_type 0.06 1 0.3384 0.5607473

## Residuals 1130.12 6284

## ---

## Signif. codes: 0 '***' 0.001 '**' 0.01 '*' 0.05 '.' 0.1 ' ' 1

##

## $`Vaginal-prelabor`

## Anova Table (Type III tests)

##

## Response: m_under[[bio]]

## Sum Sq Df F value Pr(>F)

## (Intercept) 556.39 1 3136.4867 < 2.2e-16 ***

## AGE_AT_COLLECTION 0.00 1 0.0072 0.932423

## ALDER_MODER 0.08 1 0.4362 0.508991

## BMI_MODER 0.49 1 2.7417 0.097809 .

## gest 2.84 3 5.3422 0.001130 **

## Birth_type 1.18 1 6.6789 0.009778 **

## Residuals 1124.49 6339

## ---

## Signif. codes: 0 '***' 0.001 '**' 0.01 '*' 0.05 '.' 0.1 ' ' 1

##

## $`Inlabor-prelabor`

## Anova Table (Type III tests)

##

## Response: m_vaginalt[[bio]]

## Sum Sq Df F value Pr(>F)

## (Intercept) 117.244 1 608.2396 <2e-16 ***

## AGE_AT_COLLECTION 0.269 1 1.3957 0.2377

## ALDER_MODER 0.000 1 0.0004 0.9846

## BMI_MODER 0.028 1 0.1455 0.7029

## gest 0.888 3 1.5350 0.2037

## Birth_type 0.055 1 0.2846 0.5938

## Residuals 253.480 1315

## ---

## Signif. codes: 0 '***' 0.001 '**' 0.01 '*' 0.05 '.' 0.1 ' ' 1

##

## $`Pairwise test`

## contrast gest estimate SE df t.ratio p.value

## (In-labor) - (Pre-labor) 37 0.019079 0.0771 6966 0.248 1.0000

## (In-labor) - Vaginal 37 0.013396 0.0689 6966 0.195 1.0000

## (Pre-labor) - Vaginal 37 -0.005684 0.0500 6966 -0.114 1.0000

## (In-labor) - (Pre-labor) 38 -0.020292 0.0518 6966 -0.392 1.0000

## (In-labor) - Vaginal 38 -0.059658 0.0484 6966 -1.232 1.0000

## (Pre-labor) - Vaginal 38 -0.039365 0.0298 6966 -1.320 1.0000

## (In-labor) - (Pre-labor) 39 0.055374 0.0473 6966 1.170 1.0000

## (In-labor) - Vaginal 39 -0.015200 0.0407 6966 -0.373 1.0000

## (Pre-labor) - Vaginal 39 -0.070574 0.0295 6966 -2.393 0.2006

## (In-labor) - (Pre-labor) 40+ 0.042433 0.0698 6966 0.608 1.0000

## (In-labor) - Vaginal 40+ -0.000924 0.0228 6966 -0.040 1.0000

## (Pre-labor) - Vaginal 40+ -0.043357 0.0668 6966 -0.649 1.0000

##

## P value adjustment: holm method for 12 tests

gest_type(bio="logHSP70")

## $`Vaginal-inlabor`

## Anova Table (Type III tests)

##

## Response: m_planlagt[[bio]]

## Sum Sq Df F value Pr(>F)

## (Intercept) 8423.9 1 90457.3249 < 2.2e-16 ***

## AGE_AT_COLLECTION 0.1 1 1.5827 0.2084190

## ALDER_MODER 0.1 1 0.8442 0.3582225

## BMI_MODER 0.7 1 7.0485 0.0079532 **

## gest 1.9 3 6.6450 0.0001776 ***

## Birth_type 0.2 1 1.9451 0.1631669

## Residuals 585.2 6284

## ---

## Signif. codes: 0 '***' 0.001 '**' 0.01 '*' 0.05 '.' 0.1 ' ' 1

##

## $`Vaginal-prelabor`

## Anova Table (Type III tests)

##

## Response: m_under[[bio]]

## Sum Sq Df F value Pr(>F)

## (Intercept) 8523.3 1 91790.5373 < 2.2e-16 ***

## AGE_AT_COLLECTION 0.1 1 1.2452 0.26451

## ALDER_MODER 0.0 1 0.1667 0.68307

## BMI_MODER 0.6 1 6.6169 0.01012 *

## gest 2.3 3 8.1764 1.980e-05 ***

## Birth_type 3.8 1 41.0153 1.619e-10 ***

## Residuals 588.6 6339

## ---

## Signif. codes: 0 '***' 0.001 '**' 0.01 '*' 0.05 '.' 0.1 ' ' 1

##

## $`Inlabor-prelabor`

## Anova Table (Type III tests)

##

## Response: m_vaginalt[[bio]]

## Sum Sq Df F value Pr(>F)

## (Intercept) 1789.03 1 19840.2841 < 2.2e-16 ***

## AGE_AT_COLLECTION 0.02 1 0.1932 0.6604

## ALDER_MODER 0.03 1 0.2924 0.5888

## BMI_MODER 0.20 1 2.2382 0.1349

## gest 0.08 3 0.2998 0.8256

## Birth_type 1.63 1 18.1068 2.237e-05 ***

## Residuals 118.58 1315

## ---

## Signif. codes: 0 '***' 0.001 '**' 0.01 '*' 0.05 '.' 0.1 ' ' 1

##

## $`Pairwise test`

## contrast gest estimate SE df t.ratio p.value

## (In-labor) - (Pre-labor) 37 0.000881 0.0553 6966 0.016 1.0000

## (In-labor) - Vaginal 37 -0.062490 0.0494 6966 -1.265 0.9169

## (Pre-labor) - Vaginal 37 -0.063371 0.0359 6966 -1.766 0.6198

## (In-labor) - (Pre-labor) 38 0.086002 0.0371 6966 2.316 0.1855

## (In-labor) - Vaginal 38 -0.015755 0.0348 6966 -0.453 1.0000

## (Pre-labor) - Vaginal 38 -0.101757 0.0214 6966 -4.755 <.0001

## (In-labor) - (Pre-labor) 39 0.130804 0.0340 6966 3.852 0.0012

## (In-labor) - Vaginal 39 0.042185 0.0292 6966 1.443 0.8950

## (Pre-labor) - Vaginal 39 -0.088619 0.0212 6966 -4.188 0.0003

## (In-labor) - (Pre-labor) 40+ 0.066670 0.0501 6966 1.331 0.9169

## (In-labor) - Vaginal 40+ 0.026442 0.0164 6966 1.614 0.7460

## (Pre-labor) - Vaginal 40+ -0.040229 0.0479 6966 -0.840 1.0000

##

## P value adjustment: holm method for 12 tests

gest_type(bio="logSTNF_RI")

## $`Vaginal-inlabor`

## Anova Table (Type III tests)

##

## Response: m_planlagt[[bio]]

## Sum Sq Df F value Pr(>F)

## (Intercept) 1462.59 1 5090.2424 < 2.2e-16 ***

## AGE_AT_COLLECTION 41.13 1 143.1488 < 2.2e-16 ***

## ALDER_MODER 2.81 1 9.7771 0.0017751 **

## BMI_MODER 3.54 1 12.3302 0.0004488 ***

## gest 1.54 3 1.7862 0.1474547

## Birth_type 1.22 1 4.2417 0.0394847 *

## Residuals 1805.60 6284

## ---

## Signif. codes: 0 '***' 0.001 '**' 0.01 '*' 0.05 '.' 0.1 ' ' 1

##

## $`Vaginal-prelabor`

## Anova Table (Type III tests)

##

## Response: m_under[[bio]]

## Sum Sq Df F value Pr(>F)

## (Intercept) 1477.28 1 5204.2116 < 2.2e-16 ***

## AGE_AT_COLLECTION 42.45 1 149.5534 < 2.2e-16 ***

## ALDER_MODER 2.63 1 9.2514 0.002363 **

## BMI_MODER 2.69 1 9.4758 0.002091 **

## gest 2.23 3 2.6206 0.049049 *

## Birth_type 0.00 1 0.0112 0.915559

## Residuals 1799.40 6339

## ---

## Signif. codes: 0 '***' 0.001 '**' 0.01 '*' 0.05 '.' 0.1 ' ' 1

##

## $`Inlabor-prelabor`

## Anova Table (Type III tests)

##

## Response: m_vaginalt[[bio]]

## Sum Sq Df F value Pr(>F)

## (Intercept) 294.86 1 1038.7270 < 2.2e-16 ***

## AGE_AT_COLLECTION 4.14 1 14.5935 0.0001396 ***

## ALDER_MODER 0.78 1 2.7559 0.0971352 .

## BMI_MODER 0.03 1 0.1060 0.7448254

## gest 1.75 3 2.0547 0.1044456

## Birth_type 0.17 1 0.5939 0.4410622

## Residuals 373.28 1315

## ---

## Signif. codes: 0 '***' 0.001 '**' 0.01 '*' 0.05 '.' 0.1 ' ' 1

##

## $`Pairwise test`

## contrast gest estimate SE df t.ratio p.value

## (In-labor) - (Pre-labor) 37 0.0213 0.0971 6966 0.219 1.0000

## (In-labor) - Vaginal 37 0.0578 0.0867 6966 0.667 1.0000

## (Pre-labor) - Vaginal 37 0.0365 0.0630 6966 0.580 1.0000

## (In-labor) - (Pre-labor) 38 0.0403 0.0652 6966 0.619 1.0000

## (In-labor) - Vaginal 38 0.0561 0.0610 6966 0.920 1.0000

## (Pre-labor) - Vaginal 38 0.0158 0.0376 6966 0.420 1.0000

## (In-labor) - (Pre-labor) 39 0.0537 0.0596 6966 0.902 1.0000

## (In-labor) - Vaginal 39 0.0164 0.0513 6966 0.319 1.0000

## (Pre-labor) - Vaginal 39 -0.0374 0.0371 6966 -1.006 1.0000

## (In-labor) - (Pre-labor) 40+ 0.0158 0.0879 6966 0.180 1.0000

## (In-labor) - Vaginal 40+ 0.0513 0.0288 6966 1.784 0.8944

## (Pre-labor) - Vaginal 40+ 0.0355 0.0841 6966 0.422 1.0000

##

## P value adjustment: holm method for 12 tests

gest_type(bio="logEGF")

## $`Vaginal-inlabor`

## Anova Table (Type III tests)

##

## Response: m_planlagt[[bio]]

## Sum Sq Df F value Pr(>F)

## (Intercept) 561.99 1 2602.3201 < 2.2e-16 ***

## AGE_AT_COLLECTION 2.65 1 12.2793 0.0004612 ***

## ALDER_MODER 0.88 1 4.0954 0.0430412 *

## BMI_MODER 0.84 1 3.8704 0.0491876 *

## gest 0.49 3 0.7491 0.5227347

## Birth_type 0.19 1 0.8662 0.3520413

## Residuals 1357.07 6284

## ---

## Signif. codes: 0 '***' 0.001 '**' 0.01 '*' 0.05 '.' 0.1 ' ' 1

##

## $`Vaginal-prelabor`

## Anova Table (Type III tests)

##

## Response: m_under[[bio]]

## Sum Sq Df F value Pr(>F)

## (Intercept) 578.08 1 2735.0902 < 2.2e-16 ***

## AGE_AT_COLLECTION 2.36 1 11.1602 0.0008406 ***

## ALDER_MODER 0.48 1 2.2606 0.1327567

## BMI_MODER 0.65 1 3.0779 0.0794098 .

## gest 0.47 3 0.7416 0.5271374

## Birth_type 0.89 1 4.2045 0.0403583 *

## Residuals 1339.78 6339

## ---

## Signif. codes: 0 '***' 0.001 '**' 0.01 '*' 0.05 '.' 0.1 ' ' 1

##

## $`Inlabor-prelabor`

## Anova Table (Type III tests)

##

## Response: m_vaginalt[[bio]]

## Sum Sq Df F value Pr(>F)

## (Intercept) 117.995 1 596.7094 < 2e-16 ***

## AGE_AT_COLLECTION 0.232 1 1.1709 0.27941

## ALDER_MODER 0.019 1 0.0960 0.75668

## BMI_MODER 0.120 1 0.6077 0.43581

## gest 1.322 3 2.2277 0.08324 .

## Birth_type 0.001 1 0.0076 0.93065

## Residuals 260.032 1315

## ---

## Signif. codes: 0 '***' 0.001 '**' 0.01 '*' 0.05 '.' 0.1 ' ' 1

##

## $`Pairwise test`

## contrast gest estimate SE df t.ratio p.value

## (In-labor) - (Pre-labor) 37 -0.02759 0.0836 6966 -0.330 1.0000

## (In-labor) - Vaginal 37 0.04445 0.0747 6966 0.595 1.0000

## (Pre-labor) - Vaginal 37 0.07204 0.0543 6966 1.327 1.0000

## (In-labor) - (Pre-labor) 38 0.03588 0.0562 6966 0.639 1.0000

## (In-labor) - Vaginal 38 0.09697 0.0526 6966 1.844 0.7175

## (Pre-labor) - Vaginal 38 0.06108 0.0324 6966 1.887 0.7109

## (In-labor) - (Pre-labor) 39 0.02410 0.0514 6966 0.469 1.0000

## (In-labor) - Vaginal 39 0.03316 0.0442 6966 0.750 1.0000

## (Pre-labor) - Vaginal 39 0.00906 0.0320 6966 0.283 1.0000

## (In-labor) - (Pre-labor) 40+ -0.07611 0.0758 6966 -1.004 1.0000

## (In-labor) - Vaginal 40+ -0.00733 0.0248 6966 -0.296 1.0000

## (Pre-labor) - Vaginal 40+ 0.06878 0.0725 6966 0.949 1.0000

##

## P value adjustment: holm method for 12 tests

gest_type(bio="logVEGF")

## $`Vaginal-inlabor`

## Anova Table (Type III tests)

##

## Response: m_planlagt[[bio]]

## Sum Sq Df F value Pr(>F)

## (Intercept) 963.53 1 4915.7213 < 2e-16 ***

## AGE_AT_COLLECTION 41.42 1 211.3075 < 2e-16 ***

## ALDER_MODER 0.63 1 3.1995 0.07371 .

## BMI_MODER 1.07 1 5.4495 0.01960 *

## gest 0.48 3 0.8224 0.48131

## Birth_type 0.89 1 4.5474 0.03301 *

## Residuals 1231.73 6284

## ---

## Signif. codes: 0 '***' 0.001 '**' 0.01 '*' 0.05 '.' 0.1 ' ' 1

##

## $`Vaginal-prelabor`

## Anova Table (Type III tests)

##

## Response: m_under[[bio]]

## Sum Sq Df F value Pr(>F)

## (Intercept) 1015.87 1 5240.5285 < 2.2e-16 ***

## AGE_AT_COLLECTION 45.91 1 236.8116 < 2.2e-16 ***

## ALDER_MODER 0.53 1 2.7471 0.09748 .

## BMI_MODER 0.63 1 3.2443 0.07172 .

## gest 0.71 3 1.2277 0.29787

## Birth_type 2.95 1 15.2384 9.574e-05 ***

## Residuals 1228.81 6339

## ---

## Signif. codes: 0 '***' 0.001 '**' 0.01 '*' 0.05 '.' 0.1 ' ' 1

##

## $`Inlabor-prelabor`

## Anova Table (Type III tests)

##

## Response: m_vaginalt[[bio]]

## Sum Sq Df F value Pr(>F)

## (Intercept) 199.615 1 1032.9848 < 2.2e-16 ***

## AGE_AT_COLLECTION 8.816 1 45.6210 2.149e-11 ***

## ALDER_MODER 0.000 1 0.0008 0.9777

## BMI_MODER 0.012 1 0.0638 0.8006

## gest 0.828 3 1.4284 0.2328

## Birth_type 0.250 1 1.2924 0.2558

## Residuals 254.112 1315

## ---

## Signif. codes: 0 '***' 0.001 '**' 0.01 '*' 0.05 '.' 0.1 ' ' 1

##

## $`Pairwise test`

## contrast gest estimate SE df t.ratio p.value

## (In-labor) - (Pre-labor) 37 -0.04228 0.0802 6966 -0.527 1.0000

## (In-labor) - Vaginal 37 0.03635 0.0716 6966 0.508 1.0000

## (Pre-labor) - Vaginal 37 0.07863 0.0520 6966 1.511 1.0000

## (In-labor) - (Pre-labor) 38 -0.06852 0.0538 6966 -1.273 1.0000

## (In-labor) - Vaginal 38 0.04686 0.0504 6966 0.930 1.0000

## (Pre-labor) - Vaginal 38 0.11538 0.0310 6966 3.719 0.0024

## (In-labor) - (Pre-labor) 39 0.00864 0.0492 6966 0.176 1.0000

## (In-labor) - Vaginal 39 0.03805 0.0424 6966 0.898 1.0000

## (Pre-labor) - Vaginal 39 0.02941 0.0307 6966 0.959 1.0000

## (In-labor) - (Pre-labor) 40+ -0.08855 0.0726 6966 -1.219 1.0000

## (In-labor) - Vaginal 40+ 0.03862 0.0237 6966 1.626 1.0000

## (Pre-labor) - Vaginal 40+ 0.12717 0.0694 6966 1.831 0.7382

##

## P value adjustment: holm method for 12 tests

gest_type(bio="logS100B")

## $`Vaginal-inlabor`

## Anova Table (Type III tests)

##

## Response: m_planlagt[[bio]]

## Sum Sq Df F value Pr(>F)

## (Intercept) 1884.50 1 6196.7963 < 2e-16 ***

## AGE_AT_COLLECTION 0.32 1 1.0370 0.30856

## ALDER_MODER 0.40 1 1.3145 0.25162

## BMI_MODER 0.01 1 0.0368 0.84781

## gest 1.53 3 1.6794 0.16913

## Birth_type 0.88 1 2.8812 0.08967 .

## Residuals 1911.02 6284

## ---

## Signif. codes: 0 '***' 0.001 '**' 0.01 '*' 0.05 '.' 0.1 ' ' 1

##

## $`Vaginal-prelabor`

## Anova Table (Type III tests)

##

## Response: m_under[[bio]]

## Sum Sq Df F value Pr(>F)

## (Intercept) 1935.27 1 6332.6268 < 2e-16 ***

## AGE_AT_COLLECTION 0.12 1 0.3991 0.52760

## ALDER_MODER 0.03 1 0.1112 0.73877

## BMI_MODER 0.02 1 0.0812 0.77564

## gest 1.52 3 1.6581 0.17380

## Birth_type 1.14 1 3.7322 0.05342 .

## Residuals 1937.22 6339

## ---

## Signif. codes: 0 '***' 0.001 '**' 0.01 '*' 0.05 '.' 0.1 ' ' 1

##

## $`Inlabor-prelabor`

## Anova Table (Type III tests)

##

## Response: m_vaginalt[[bio]]

## Sum Sq Df F value Pr(>F)

## (Intercept) 381.41 1 1178.7818 <2e-16 ***

## AGE_AT_COLLECTION 1.24 1 3.8351 0.0504 .

## ALDER_MODER 0.05 1 0.1662 0.6836

## BMI_MODER 0.00 1 0.0001 0.9927

## gest 0.93 3 0.9543 0.4136

## Birth_type 0.22 1 0.6916 0.4058

## Residuals 425.49 1315

## ---

## Signif. codes: 0 '***' 0.001 '**' 0.01 '*' 0.05 '.' 0.1 ' ' 1

##

## $`Pairwise test`

## contrast gest estimate SE df t.ratio p.value

## (In-labor) - (Pre-labor) 37 -0.02215 0.1006 6966 -0.220 1.0000

## (In-labor) - Vaginal 37 0.08658 0.0899 6966 0.963 1.0000

## (Pre-labor) - Vaginal 37 0.10874 0.0653 6966 1.666 1.0000

## (In-labor) - (Pre-labor) 38 -0.02459 0.0676 6966 -0.364 1.0000

## (In-labor) - Vaginal 38 0.03035 0.0632 6966 0.480 1.0000

## (Pre-labor) - Vaginal 38 0.05494 0.0389 6966 1.411 1.0000

## (In-labor) - (Pre-labor) 39 0.00826 0.0618 6966 0.134 1.0000

## (In-labor) - Vaginal 39 0.01350 0.0532 6966 0.254 1.0000

## (Pre-labor) - Vaginal 39 0.00524 0.0385 6966 0.136 1.0000

## (In-labor) - (Pre-labor) 40+ -0.10404 0.0911 6966 -1.142 1.0000

## (In-labor) - Vaginal 40+ 0.04388 0.0298 6966 1.472 1.0000

## (Pre-labor) - Vaginal 40+ 0.14792 0.0871 6966 1.697 1.0000

##

## P value adjustment: holm method for 12 tests

gest_type(bio="logBDNF")

## $`Vaginal-inlabor`

## Anova Table (Type III tests)

##

## Response: m_planlagt[[bio]]

## Sum Sq Df F value Pr(>F)

## (Intercept) 2111.4 1 3810.0499 < 2.2e-16 ***

## AGE_AT_COLLECTION 5.3 1 9.6067 0.001947 **

## ALDER_MODER 4.1 1 7.4732 0.006280 **

## BMI_MODER 6.0 1 10.7721 0.001036 **

## gest 1.5 3 0.9091 0.435676

## Birth_type 0.1 1 0.1078 0.742678

## Residuals 3482.4 6284

## ---

## Signif. codes: 0 '***' 0.001 '**' 0.01 '*' 0.05 '.' 0.1 ' ' 1

##

## $`Vaginal-prelabor`

## Anova Table (Type III tests)

##

## Response: m_under[[bio]]

## Sum Sq Df F value Pr(>F)

## (Intercept) 2226.4 1 4083.9207 < 2.2e-16 ***

## AGE_AT_COLLECTION 5.2 1 9.5859 0.001969 **

## ALDER_MODER 5.0 1 9.2000 0.002430 **

## BMI_MODER 4.7 1 8.7032 0.003188 **

## gest 1.0 3 0.6171 0.603868

## Birth_type 0.9 1 1.6892 0.193757

## Residuals 3455.7 6339

## ---

## Signif. codes: 0 '***' 0.001 '**' 0.01 '*' 0.05 '.' 0.1 ' ' 1

##

## $`Inlabor-prelabor`

## Anova Table (Type III tests)

##

## Response: m_vaginalt[[bio]]

## Sum Sq Df F value Pr(>F)

## (Intercept) 448.85 1 804.3313 <2e-16 ***

## AGE_AT_COLLECTION 0.23 1 0.4096 0.5223

## ALDER_MODER 1.33 1 2.3748 0.1235

## BMI_MODER 0.91 1 1.6271 0.2023

## gest 0.79 3 0.4700 0.7032

## Birth_type 0.98 1 1.7581 0.1851

## Residuals 733.83 1315

## ---

## Signif. codes: 0 '***' 0.001 '**' 0.01 '*' 0.05 '.' 0.1 ' ' 1

##

## $`Pairwise test`

## contrast gest estimate SE df t.ratio p.value

## (In-labor) - (Pre-labor) 37 -0.1421 0.1347 6966 -1.055 1.0000

## (In-labor) - Vaginal 37 -0.0876 0.1204 6966 -0.728 1.0000

## (Pre-labor) - Vaginal 37 0.0545 0.0875 6966 0.623 1.0000

## (In-labor) - (Pre-labor) 38 0.0508 0.0905 6966 0.562 1.0000

## (In-labor) - Vaginal 38 0.0713 0.0847 6966 0.842 1.0000

## (Pre-labor) - Vaginal 38 0.0205 0.0522 6966 0.392 1.0000

## (In-labor) - (Pre-labor) 39 -0.0637 0.0827 6966 -0.770 1.0000

## (In-labor) - Vaginal 39 -0.0279 0.0713 6966 -0.391 1.0000

## (Pre-labor) - Vaginal 39 0.0358 0.0516 6966 0.695 1.0000

## (In-labor) - (Pre-labor) 40+ -0.1924 0.1221 6966 -1.575 1.0000

## (In-labor) - Vaginal 40+ -0.0149 0.0399 6966 -0.372 1.0000

## (Pre-labor) - Vaginal 40+ 0.1775 0.1167 6966 1.520 1.0000

##

## P value adjustment: holm method for 12 tests

gest_type(bio="logNT3")

## $`Vaginal-inlabor`

## Anova Table (Type III tests)

##

## Response: m_planlagt[[bio]]

## Sum Sq Df F value Pr(>F)

## (Intercept) 121.02 1 494.3439 < 2.2e-16 ***

## AGE_AT_COLLECTION 5.52 1 22.5565 2.086e-06 ***

## ALDER_MODER 1.03 1 4.1898 0.04071 *

## BMI_MODER 0.60 1 2.4571 0.11705

## gest 0.75 3 1.0252 0.38019

## Birth_type 0.34 1 1.4030 0.23626

## Residuals 1538.34 6284

## ---

## Signif. codes: 0 '***' 0.001 '**' 0.01 '*' 0.05 '.' 0.1 ' ' 1

##

## $`Vaginal-prelabor`

## Anova Table (Type III tests)

##

## Response: m_under[[bio]]

## Sum Sq Df F value Pr(>F)

## (Intercept) 123.49 1 512.3227 < 2.2e-16 ***

## AGE_AT_COLLECTION 5.37 1 22.2919 2.392e-06 ***

## ALDER_MODER 0.92 1 3.8183 0.05074 .

## BMI_MODER 0.63 1 2.6056 0.10653

## gest 0.64 3 0.8841 0.44847

## Birth_type 0.02 1 0.1015 0.75004

## Residuals 1527.96 6339

## ---

## Signif. codes: 0 '***' 0.001 '**' 0.01 '*' 0.05 '.' 0.1 ' ' 1

##

## $`Inlabor-prelabor`

## Anova Table (Type III tests)

##

## Response: m_vaginalt[[bio]]

## Sum Sq Df F value Pr(>F)

## (Intercept) 18.005 1 82.8325 <2e-16 ***

## AGE_AT_COLLECTION 0.084 1 0.3875 0.5337

## ALDER_MODER 0.008 1 0.0377 0.8462

## BMI_MODER 0.205 1 0.9412 0.3321

## gest 0.862 3 1.3226 0.2654

## Birth_type 0.000 1 0.0011 0.9736

## Residuals 285.830 1315

## ---

## Signif. codes: 0 '***' 0.001 '**' 0.01 '*' 0.05 '.' 0.1 ' ' 1

##

## $`Pairwise test`

## contrast gest estimate SE df t.ratio p.value

## (In-labor) - (Pre-labor) 37 -0.0872 0.0891 6966 -0.979 1.0000

## (In-labor) - Vaginal 37 -0.0622 0.0796 6966 -0.782 1.0000

## (Pre-labor) - Vaginal 37 0.0250 0.0578 6966 0.432 1.0000

## (In-labor) - (Pre-labor) 38 0.0198 0.0598 6966 0.331 1.0000

## (In-labor) - Vaginal 38 0.0541 0.0560 6966 0.966 1.0000

## (Pre-labor) - Vaginal 38 0.0343 0.0345 6966 0.994 1.0000

## (In-labor) - (Pre-labor) 39 0.0725 0.0547 6966 1.326 1.0000

## (In-labor) - Vaginal 39 0.0390 0.0471 6966 0.828 1.0000

## (Pre-labor) - Vaginal 39 -0.0335 0.0341 6966 -0.984 1.0000

## (In-labor) - (Pre-labor) 40+ -0.0385 0.0807 6966 -0.477 1.0000

## (In-labor) - Vaginal 40+ 0.0227 0.0264 6966 0.862 1.0000

## (Pre-labor) - Vaginal 40+ 0.0613 0.0772 6966 0.794 1.0000

##

## P value adjustment: holm method for 12 tests

S1.3 Testing gender

Figure 2 in the paper

Splitting the dataset in boys and girls to get an overall p-value for each of the genders

Testing

koen_type(bio="logCRP")

## $Boys

## Anova Table (Type III tests)

##

## Response: boys[[bio]]

## Sum Sq Df F value Pr(>F)

## (Intercept) 28.88 1 52.5955 4.950e-13 ***

## AGE_AT_COLLECTION 11.18 1 20.3539 6.633e-06 ***

## ALDER_MODER 0.67 1 1.2173 0.27

## BMI_MODER 0.50 1 0.9107 0.34

## Birth_type 70.00 2 63.7376 < 2.2e-16 ***

## Residuals 2068.04 3766

## ---

## Signif. codes: 0 '***' 0.001 '**' 0.01 '*' 0.05 '.' 0.1 ' ' 1

##

## $Girls

## Anova Table (Type III tests)

##

## Response: girls[[bio]]

## Sum Sq Df F value Pr(>F)

## (Intercept) 27.71 1 50.3332 1.590e-12 ***

## AGE_AT_COLLECTION 9.12 1 16.5665 4.811e-05 ***

## ALDER_MODER 3.40 1 6.1725 0.01303 *

## BMI_MODER 1.41 1 2.5622 0.10954

## Birth_type 98.99 2 89.9111 < 2.2e-16 ***

## Residuals 1763.22 3203

## ---

## Signif. codes: 0 '***' 0.001 '**' 0.01 '*' 0.05 '.' 0.1 ' ' 1

##

## $`Pairwise test`

## contrast Birth_type estimate SE df t.ratio p.value

## K - M In-labor -0.154 0.0601 6972 -2.567 0.0103

## K - M Pre-labor -0.267 0.0566 6972 -4.721 <.0001

## K - M Vaginal -0.140 0.0198 6972 -7.060 <.0001

##

## P value adjustment: holm method for 3 tests

koen_type(bio="logMCP1")

## $Boys

## Anova Table (Type III tests)

##

## Response: boys[[bio]]

## Sum Sq Df F value Pr(>F)

## (Intercept) 1437.30 1 7684.4110 <2e-16 ***

## AGE_AT_COLLECTION 25.88 1 138.3724 <2e-16 ***

## ALDER_MODER 0.00 1 0.0137 0.9068

## BMI_MODER 0.05 1 0.2690 0.6040

## Birth_type 15.90 2 42.5066 <2e-16 ***

## Residuals 704.40 3766

## ---

## Signif. codes: 0 '***' 0.001 '**' 0.01 '*' 0.05 '.' 0.1 ' ' 1

##

## $Girls

## Anova Table (Type III tests)

##

## Response: girls[[bio]]

## Sum Sq Df F value Pr(>F)

## (Intercept) 1184.91 1 6677.8252 < 2.2e-16 ***

## AGE_AT_COLLECTION 28.36 1 159.8077 < 2.2e-16 ***

## ALDER_MODER 0.53 1 3.0140 0.08265 .

## BMI_MODER 0.02 1 0.0918 0.76196

## Birth_type 12.70 2 35.7965 4.217e-16 ***

## Residuals 568.34 3203

## ---

## Signif. codes: 0 '***' 0.001 '**' 0.01 '*' 0.05 '.' 0.1 ' ' 1

##

## $`Pairwise test`

## contrast Birth_type estimate SE df t.ratio p.value

## K - M In-labor -0.0519 0.0346 6972 -1.499 0.1340

## K - M Pre-labor -0.0616 0.0326 6972 -1.888 0.1182

## K - M Vaginal -0.0753 0.0114 6972 -6.609 <.0001

##

## P value adjustment: holm method for 3 tests

koen_type(bio="logIL18")

## $Boys

## Anova Table (Type III tests)

##

## Response: boys[[bio]]

## Sum Sq Df F value Pr(>F)

## (Intercept) 425.74 1 2333.2559 < 2.2e-16 ***

## AGE_AT_COLLECTION 0.00 1 0.0130 0.909206

## ALDER_MODER 0.00 1 0.0003 0.986187

## BMI_MODER 0.58 1 3.2033 0.073572 .

## Birth_type 1.90 2 5.1965 0.005575 **

## Residuals 687.16 3766

## ---

## Signif. codes: 0 '***' 0.001 '**' 0.01 '*' 0.05 '.' 0.1 ' ' 1

##

## $Girls

## Anova Table (Type III tests)

##

## Response: girls[[bio]]

## Sum Sq Df F value Pr(>F)

## (Intercept) 341.40 1 1917.6331 < 2e-16 ***

## AGE_AT_COLLECTION 0.05 1 0.2876 0.59183

## ALDER_MODER 0.08 1 0.4653 0.49519

## BMI_MODER 0.08 1 0.4299 0.51209

## Birth_type 1.27 2 3.5552 0.02869 *

## Residuals 570.23 3203

## ---

## Signif. codes: 0 '***' 0.001 '**' 0.01 '*' 0.05 '.' 0.1 ' ' 1

##

## $`Pairwise test`

## contrast Birth_type estimate SE df t.ratio p.value

## K - M In-labor -0.01532 0.0344 6972 -0.445 1.0000

## K - M Pre-labor -0.00123 0.0324 6972 -0.038 1.0000

## K - M Vaginal -0.01471 0.0113 6972 -1.299 0.5820

##

## P value adjustment: holm method for 3 tests

koen_type(bio="logHSP70")

## $Boys

## Anova Table (Type III tests)

##

## Response: boys[[bio]]

## Sum Sq Df F value Pr(>F)

## (Intercept) 6174.3 1 68337.9687 < 2.2e-16 ***

## AGE_AT_COLLECTION 0.2 1 2.5344 0.111476

## ALDER_MODER 0.0 1 0.3401 0.559814

## BMI_MODER 0.4 1 4.4442 0.035085 *

## Birth_type 1.2 2 6.3920 0.001693 **

## Residuals 340.3 3766

## ---

## Signif. codes: 0 '***' 0.001 '**' 0.01 '*' 0.05 '.' 0.1 ' ' 1

##

## $Girls

## Anova Table (Type III tests)

##

## Response: girls[[bio]]

## Sum Sq Df F value Pr(>F)

## (Intercept) 4990.8 1 52006.1930 < 2.2e-16 ***

## AGE_AT_COLLECTION 0.0 1 0.0294 0.86391

## ALDER_MODER 0.2 1 2.4271 0.11936

## BMI_MODER 0.3 1 2.8499 0.09148 .

## Birth_type 1.8 2 9.5214 7.537e-05 ***

## Residuals 307.4 3203

## ---

## Signif. codes: 0 '***' 0.001 '**' 0.01 '*' 0.05 '.' 0.1 ' ' 1

##

## $`Pairwise test`

## contrast Birth_type estimate SE df t.ratio p.value

## K - M In-labor 0.00607 0.02470 6972 0.246 0.8857

## K - M Pre-labor -0.01786 0.02327 6972 -0.767 0.8857

## K - M Vaginal 0.01492 0.00813 6972 1.835 0.1995

##

## P value adjustment: holm method for 3 tests

koen_type(bio="logSTNF_RI")

## $Boys

## Anova Table (Type III tests)

##

## Response: boys[[bio]]

## Sum Sq Df F value Pr(>F)

## (Intercept) 1004.09 1 3608.4393 < 2e-16 ***

## AGE_AT_COLLECTION 20.19 1 72.5695 < 2e-16 ***

## ALDER_MODER 0.50 1 1.7830 0.18186

## BMI_MODER 0.39 1 1.3885 0.23874

## Birth_type 1.37 2 2.4692 0.08479 .

## Residuals 1047.93 3766

## ---

## Signif. codes: 0 '***' 0.001 '**' 0.01 '*' 0.05 '.' 0.1 ' ' 1

##

## $Girls

## Anova Table (Type III tests)

##

## Response: girls[[bio]]

## Sum Sq Df F value Pr(>F)

## (Intercept) 920.24 1 3139.9160 < 2.2e-16 ***

## AGE_AT_COLLECTION 23.25 1 79.3338 < 2.2e-16 ***

## ALDER_MODER 3.48 1 11.8893 0.0005718 ***

## BMI_MODER 3.65 1 12.4505 0.0004238 ***

## Birth_type 0.57 2 0.9716 0.3785729

## Residuals 938.72 3203

## ---

## Signif. codes: 0 '***' 0.001 '**' 0.01 '*' 0.05 '.' 0.1 ' ' 1

##

## $`Pairwise test`

## contrast Birth_type estimate SE df t.ratio p.value

## K - M In-labor 0.0362 0.0433 6972 0.838 0.8044

## K - M Pre-labor -0.0111 0.0408 6972 -0.272 0.8044

## K - M Vaginal 0.0493 0.0142 6972 3.459 0.0016

##

## P value adjustment: holm method for 3 tests

koen_type(bio="logEGF")

## $Boys

## Anova Table (Type III tests)

##

## Response: boys[[bio]]

## Sum Sq Df F value Pr(>F)

## (Intercept) 375.74 1 1747.6043 < 2e-16 ***

## AGE_AT_COLLECTION 1.35 1 6.2840 0.01223 *

## ALDER_MODER 0.01 1 0.0511 0.82121

## BMI_MODER 0.14 1 0.6470 0.42125

## Birth_type 0.50 2 1.1519 0.31615

## Residuals 809.70 3766

## ---

## Signif. codes: 0 '***' 0.001 '**' 0.01 '*' 0.05 '.' 0.1 ' ' 1

##

## $Girls

## Anova Table (Type III tests)

##

## Response: girls[[bio]]

## Sum Sq Df F value Pr(>F)

## (Intercept) 354.87 1 1714.6464 < 2e-16 ***

## AGE_AT_COLLECTION 1.17 1 5.6488 0.01753 *

## ALDER_MODER 1.25 1 6.0350 0.01408 *

## BMI_MODER 0.92 1 4.4624 0.03473 *

## Birth_type 1.27 2 3.0753 0.04631 *

## Residuals 662.90 3203

## ---

## Signif. codes: 0 '***' 0.001 '**' 0.01 '*' 0.05 '.' 0.1 ' ' 1

##

## $`Pairwise test`

## contrast Birth_type estimate SE df t.ratio p.value

## K - M In-labor 0.0860 0.0372 6972 2.309 0.0420

## K - M Pre-labor 0.0662 0.0351 6972 1.885 0.0595

## K - M Vaginal 0.0558 0.0123 6972 4.554 <.0001

##

## P value adjustment: holm method for 3 tests

koen_type(bio="logVEGF")

## $Boys

## Anova Table (Type III tests)

##

## Response: boys[[bio]]

## Sum Sq Df F value Pr(>F)

## (Intercept) 661.90 1 3442.0085 < 2.2e-16 ***

## AGE_AT_COLLECTION 23.31 1 121.2027 < 2.2e-16 ***

## ALDER_MODER 0.03 1 0.1715 0.678762

## BMI_MODER 0.08 1 0.4078 0.523120

## Birth_type 2.05 2 5.3331 0.004865 **

## Residuals 724.21 3766

## ---

## Signif. codes: 0 '***' 0.001 '**' 0.01 '*' 0.05 '.' 0.1 ' ' 1

##

## $Girls

## Anova Table (Type III tests)

##

## Response: girls[[bio]]

## Sum Sq Df F value Pr(>F)

## (Intercept) 611.90 1 3150.0592 < 2.2e-16 ***

## AGE_AT_COLLECTION 25.01 1 128.7281 < 2.2e-16 ***

## ALDER_MODER 0.84 1 4.3349 0.0374174 *

## BMI_MODER 1.17 1 6.0209 0.0141900 *

## Birth_type 3.49 2 8.9959 0.0001271 ***

## Residuals 622.18 3203

## ---

## Signif. codes: 0 '***' 0.001 '**' 0.01 '*' 0.05 '.' 0.1 ' ' 1

##

## $`Pairwise test`

## contrast Birth_type estimate SE df t.ratio p.value

## K - M In-labor 0.0897 0.0356 6972 2.518 0.0118

## K - M Pre-labor 0.1016 0.0336 6972 3.028 0.0049

## K - M Vaginal 0.0763 0.0117 6972 6.510 <.0001

##

## P value adjustment: holm method for 3 tests

koen_type(bio="logS100B")

## $Boys

## Anova Table (Type III tests)

##

## Response: boys[[bio]]

## Sum Sq Df F value Pr(>F)

## (Intercept) 1340.78 1 4243.8208 < 2e-16 ***

## AGE_AT_COLLECTION 0.11 1 0.3350 0.56274

## ALDER_MODER 0.04 1 0.1168 0.73251

## BMI_MODER 0.05 1 0.1466 0.70179

## Birth_type 2.44 2 3.8550 0.02126 *

## Residuals 1189.82 3766

## ---

## Signif. codes: 0 '***' 0.001 '**' 0.01 '*' 0.05 '.' 0.1 ' ' 1

##

## $Girls

## Anova Table (Type III tests)

##

## Response: girls[[bio]]

## Sum Sq Df F value Pr(>F)

## (Intercept) 1126.36 1 3804.7733 <2e-16 ***

## AGE_AT_COLLECTION 0.50 1 1.6831 0.1946

## ALDER_MODER 0.62 1 2.1093 0.1465

## BMI_MODER 0.02 1 0.0625 0.8026

## Birth_type 1.22 2 2.0586 0.1278

## Residuals 948.21 3203

## ---

## Signif. codes: 0 '***' 0.001 '**' 0.01 '*' 0.05 '.' 0.1 ' ' 1

##

## $`Pairwise test`

## contrast Birth_type estimate SE df t.ratio p.value

## K - M In-labor 0.0306 0.0449 6972 0.681 0.9916

## K - M Pre-labor -0.0222 0.0423 6972 -0.524 0.9916

## K - M Vaginal 0.0189 0.0148 6972 1.277 0.6054

##

## P value adjustment: holm method for 3 tests

koen_type(bio="logBDNF")

## $Boys

## Anova Table (Type III tests)

##

## Response: boys[[bio]]

## Sum Sq Df F value Pr(>F)

## (Intercept) 1516.75 1 2712.4699 < 2e-16 ***

## AGE_AT_COLLECTION 1.66 1 2.9728 0.08476 .

## ALDER_MODER 2.63 1 4.7081 0.03008 *

## BMI_MODER 2.52 1 4.5113 0.03374 *

## Birth_type 1.53 2 1.3701 0.25422

## Residuals 2105.86 3766

## ---

## Signif. codes: 0 '***' 0.001 '**' 0.01 '*' 0.05 '.' 0.1 ' ' 1

##

## $Girls

## Anova Table (Type III tests)

##

## Response: girls[[bio]]

## Sum Sq Df F value Pr(>F)

## (Intercept) 1372.98 1 2569.7394 < 2.2e-16 ***

## AGE_AT_COLLECTION 4.37 1 8.1773 0.004269 **

## ALDER_MODER 2.79 1 5.2185 0.022413 *

## BMI_MODER 3.41 1 6.3864 0.011547 *

## Birth_type 1.87 2 1.7512 0.173733

## Residuals 1711.33 3203

## ---

## Signif. codes: 0 '***' 0.001 '**' 0.01 '*' 0.05 '.' 0.1 ' ' 1

##

## $`Pairwise test`

## contrast Birth_type estimate SE df t.ratio p.value

## K - M In-labor 0.2229 0.0599 6972 3.718 0.0004

## K - M Pre-labor 0.1157 0.0565 6972 2.048 0.0406

## K - M Vaginal 0.0849 0.0197 6972 4.305 0.0001

##

## P value adjustment: holm method for 3 tests

koen_type(bio="logNT3")

## $Boys

## Anova Table (Type III tests)

##

## Response: boys[[bio]]

## Sum Sq Df F value Pr(>F)

## (Intercept) 81.02 1 358.3789 < 2.2e-16 ***

## AGE_AT_COLLECTION 1.95 1 8.6077 0.003368 **

## ALDER_MODER 0.17 1 0.7382 0.390282

## BMI_MODER 0.10 1 0.4361 0.509075

## Birth_type 0.10 2 0.2191 0.803259

## Residuals 851.36 3766

## ---

## Signif. codes: 0 '***' 0.001 '**' 0.01 '*' 0.05 '.' 0.1 ' ' 1

##

## $Girls

## Anova Table (Type III tests)

##

## Response: girls[[bio]]

## Sum Sq Df F value Pr(>F)

## (Intercept) 87.26 1 338.8014 < 2.2e-16 ***

## AGE_AT_COLLECTION 2.97 1 11.5344 0.0006915 ***

## ALDER_MODER 0.97 1 3.7475 0.0529735 .

## BMI_MODER 0.90 1 3.4870 0.0619420 .

## Birth_type 0.79 2 1.5410 0.2143168

## Residuals 824.97 3203

## ---

## Signif. codes: 0 '***' 0.001 '**' 0.01 '*' 0.05 '.' 0.1 ' ' 1

##

## $`Pairwise test`

## contrast Birth_type estimate SE df t.ratio p.value

## K - M In-labor 0.0618 0.0397 6972 1.555 0.3602

## K - M Pre-labor -0.0202 0.0374 6972 -0.541 0.5888

## K - M Vaginal 0.0186 0.0131 6972 1.426 0.3602

##

## P value adjustment: holm method for 3 tests

S1.4 Testing the GA for all biomarkers

Figure 3 in the paper.

bio_gest(data=MFR_3742, bio=MFR_3742$CRP)

## [[1]]

## Anova Table (Type III tests)

##

## Response: log(bio)

## Sum Sq Df F value Pr(>F)

## (Intercept) 86.4 1 157.4233 < 2.2e-16 ***

## AGE_AT_COLLECTION 11.9 1 21.7446 3.173e-06 ***

## ALDER_MODER 9.5 1 17.3155 3.204e-05 ***

## BMI_MODER 0.1 1 0.2483 0.6183

## gestation_uge_f 217.0 5 79.1219 < 2.2e-16 ***

## Residuals 3824.6 6972

## ---

## Signif. codes: 0 '***' 0.001 '**' 0.01 '*' 0.05 '.' 0.1 ' ' 1

##

## [[2]]

## [1] 0.05898508

##

## [[3]]

## contrast estimate SE df t.ratio p.value

## 37 - 38 -0.1055 0.0428 6972 -2.462 0.0415

## 37 - 39 -0.2757 0.0406 6972 -6.784 <.0001

## 37 - 40 -0.4800 0.0397 6972 -12.105 <.0001

## 37 - 41 -0.5125 0.0412 6972 -12.455 <.0001

## 37 - 42 -0.5845 0.0512 6972 -11.420 <.0001

## 38 - 39 -0.1702 0.0295 6972 -5.771 <.0001

## 38 - 40 -0.3745 0.0282 6972 -13.304 <.0001

## 38 - 41 -0.4071 0.0302 6972 -13.459 <.0001

## 38 - 42 -0.4790 0.0429 6972 -11.156 <.0001

## 39 - 40 -0.2044 0.0246 6972 -8.301 <.0001

## 39 - 41 -0.2369 0.0270 6972 -8.776 <.0001

## 39 - 42 -0.3088 0.0407 6972 -7.581 <.0001

## 40 - 41 -0.0325 0.0255 6972 -1.275 0.2025

## 40 - 42 -0.1044 0.0397 6972 -2.628 0.0345

## 41 - 42 -0.0719 0.0412 6972 -1.744 0.1624

##

## Results are given on the log (not the response) scale.

## P value adjustment: holm method for 15 tests

bio_gest(data=MFR_3742, bio=MFR_3742$MCP1)

## [[1]]

## Anova Table (Type III tests)

##

## Response: log(bio)

## Sum Sq Df F value Pr(>F)

## (Intercept) 2396.57 1 13001.2445 < 2e-16 ***

## AGE_AT_COLLECTION 48.19 1 261.4419 < 2e-16 ***

## ALDER_MODER 1.09 1 5.8986 0.01518 *

## BMI_MODER 0.20 1 1.0935 0.29573

## gestation_uge_f 25.74 5 27.9282 < 2e-16 ***

## Residuals 1285.18 6972

## ---

## Signif. codes: 0 '***' 0.001 '**' 0.01 '*' 0.05 '.' 0.1 ' ' 1

##

## [[2]]

## [1] 0.05447943

##

## [[3]]

## contrast estimate SE df t.ratio p.value

## 37 - 38 0.00926 0.0248 6972 0.373 0.9004

## 37 - 39 -0.05407 0.0236 6972 -2.295 0.1087

## 37 - 40 -0.13151 0.0230 6972 -5.721 <.0001

## 37 - 41 -0.14268 0.0239 6972 -5.981 <.0001

## 37 - 42 -0.16788 0.0297 6972 -5.658 <.0001

## 38 - 39 -0.06333 0.0171 6972 -3.704 0.0013

## 38 - 40 -0.14077 0.0163 6972 -8.626 <.0001

## 38 - 41 -0.15194 0.0175 6972 -8.666 <.0001

## 38 - 42 -0.17714 0.0249 6972 -7.117 <.0001

## 39 - 40 -0.07744 0.0143 6972 -5.427 <.0001

## 39 - 41 -0.08861 0.0156 6972 -5.663 <.0001

## 39 - 42 -0.11381 0.0236 6972 -4.820 <.0001

## 40 - 41 -0.01117 0.0148 6972 -0.755 0.9004

## 40 - 42 -0.03637 0.0230 6972 -1.578 0.4581

## 41 - 42 -0.02520 0.0239 6972 -1.054 0.8756

##

## Results are given on the log (not the response) scale.

## P value adjustment: holm method for 15 tests

bio_gest(data=MFR_3742, bio=MFR_3742$IL18)

## [[1]]

## Anova Table (Type III tests)

##

## Response: log(bio)

## Sum Sq Df F value Pr(>F)

## (Intercept) 702.28 1 3904.9687 < 2.2e-16 ***

## AGE_AT_COLLECTION 0.00 1 0.0004 0.9851

## ALDER_MODER 0.00 1 0.0010 0.9753

## BMI_MODER 0.34 1 1.8757 0.1709

## gestation_uge_f 7.20 5 8.0094 1.535e-07 ***

## Residuals 1253.85 6972

## ---

## Signif. codes: 0 '***' 0.001 '**' 0.01 '*' 0.05 '.' 0.1 ' ' 1

##

## [[2]]

## [1] 0.006071733

##

## [[3]]

## contrast estimate SE df t.ratio p.value

## 37 - 38 -0.01597 0.0245 6972 -0.651 1.0000

## 37 - 39 -0.02298 0.0233 6972 -0.987 1.0000

## 37 - 40 -0.06308 0.0227 6972 -2.778 0.0329

## 37 - 41 -0.07139 0.0236 6972 -3.030 0.0246

## 37 - 42 -0.13171 0.0293 6972 -4.494 0.0001

## 38 - 39 -0.00701 0.0169 6972 -0.415 1.0000

## 38 - 40 -0.04711 0.0161 6972 -2.923 0.0279

## 38 - 41 -0.05542 0.0173 6972 -3.200 0.0166

## 38 - 42 -0.11574 0.0246 6972 -4.708 <.0001

## 39 - 40 -0.04010 0.0141 6972 -2.845 0.0311

## 39 - 41 -0.04841 0.0155 6972 -3.132 0.0191

## 39 - 42 -0.10874 0.0233 6972 -4.662 <.0001

## 40 - 41 -0.00831 0.0146 6972 -0.569 1.0000

## 40 - 42 -0.06863 0.0228 6972 -3.016 0.0246

## 41 - 42 -0.06032 0.0236 6972 -2.555 0.0532

##

## Results are given on the log (not the response) scale.

## P value adjustment: holm method for 15 tests

bio_gest(data=MFR_3742, bio=MFR_3742$HSP70)

## [[1]]

## Anova Table (Type III tests)

##

## Response: log(bio)

## Sum Sq Df F value Pr(>F)

## (Intercept) 10438.8 1 1.1194e+05 < 2e-16 ***

## AGE_AT_COLLECTION 0.0 1 2.5560e-01 0.61316

## ALDER_MODER 0.2 1 1.7208e+00 0.18963

## BMI_MODER 0.6 1 5.9402e+00 0.01482 *

## gestation_uge_f 1.0 5 2.1302e+00 0.05889 .

## Residuals 650.2 6972

## ---

## Signif. codes: 0 '***' 0.001 '**' 0.01 '*' 0.05 '.' 0.1 ' ' 1

##

## [[2]]

## [1] 0.002654218

##

## [[3]]

## contrast estimate SE df t.ratio p.value

## 37 - 38 0.00177 0.0177 6972 0.100 1.0000

## 37 - 39 0.02814 0.0168 6972 1.679 1.0000

## 37 - 40 0.03210 0.0163 6972 1.963 0.6458

## 37 - 41 0.02058 0.0170 6972 1.213 1.0000

## 37 - 42 0.00646 0.0211 6972 0.306 1.0000

## 38 - 39 0.02637 0.0122 6972 2.168 0.4225

## 38 - 40 0.03032 0.0116 6972 2.612 0.1352

## 38 - 41 0.01881 0.0125 6972 1.508 1.0000

## 38 - 42 0.00469 0.0177 6972 0.265 1.0000

## 39 - 40 0.00396 0.0101 6972 0.390 1.0000

## 39 - 41 -0.00756 0.0111 6972 -0.679 1.0000

## 39 - 42 -0.02168 0.0168 6972 -1.291 1.0000

## 40 - 41 -0.01152 0.0105 6972 -1.095 1.0000

## 40 - 42 -0.02564 0.0164 6972 -1.564 1.0000

## 41 - 42 -0.01412 0.0170 6972 -0.830 1.0000

##

## Results are given on the log (not the response) scale.

## P value adjustment: holm method for 15 tests

bio_gest(data=MFR_3742, bio=MFR_3742$STNF_RI)

## [[1]]

## Anova Table (Type III tests)

##

## Response: log(bio)

## Sum Sq Df F value Pr(>F)

## (Intercept) 1781.40 1 6239.2216 < 2.2e-16 ***

## AGE_AT_COLLECTION 43.63 1 152.8054 < 2.2e-16 ***

## ALDER_MODER 3.12 1 10.9401 0.0009458 ***

## BMI_MODER 2.62 1 9.1623 0.0024796 **

## gestation_uge_f 3.29 5 2.3019 0.0422821 *

## Residuals 1990.62 6972

## ---

## Signif. codes: 0 '***' 0.001 '**' 0.01 '*' 0.05 '.' 0.1 ' ' 1

##

## [[2]]

## [1] 0.02560888

##

## [[3]]

## contrast estimate SE df t.ratio p.value

## 37 - 38 -0.00997 0.0309 6972 -0.323 1.0000

## 37 - 39 0.04483 0.0293 6972 1.529 1.0000

## 37 - 40 0.02839 0.0286 6972 0.992 1.0000

## 37 - 41 0.04849 0.0297 6972 1.633 1.0000

## 37 - 42 0.06131 0.0369 6972 1.660 1.0000

## 38 - 39 0.05481 0.0213 6972 2.576 0.1403

## 38 - 40 0.03836 0.0203 6972 1.889 0.7075

## 38 - 41 0.05847 0.0218 6972 2.679 0.1109

## 38 - 42 0.07128 0.0310 6972 2.301 0.2783

## 39 - 40 -0.01644 0.0178 6972 -0.926 1.0000

## 39 - 41 0.00366 0.0195 6972 0.188 1.0000

## 39 - 42 0.01648 0.0294 6972 0.561 1.0000

## 40 - 41 0.02010 0.0184 6972 1.092 1.0000

## 40 - 42 0.03292 0.0287 6972 1.148 1.0000

## 41 - 42 0.01282 0.0298 6972 0.431 1.0000

##

## Results are given on the log (not the response) scale.

## P value adjustment: holm method for 15 tests

bio_gest(data=MFR_3742, bio=MFR_3742$EGF)

## [[1]]

## Anova Table (Type III tests)

##

## Response: log(bio)

## Sum Sq Df F value Pr(>F)

## (Intercept) 685.99 1 3235.9892 < 2.2e-16 ***

## AGE_AT_COLLECTION 2.84 1 13.4114 0.000252 ***

## ALDER_MODER 0.48 1 2.2456 0.134044

## BMI_MODER 0.63 1 2.9820 0.084240 .

## gestation_uge_f 3.27 5 3.0858 0.008728 **

## Residuals 1477.97 6972

## ---

## Signif. codes: 0 '***' 0.001 '**' 0.01 '*' 0.05 '.' 0.1 ' ' 1

##

## [[2]]

## [1] 0.004901638

##

## [[3]]

## contrast estimate SE df t.ratio p.value

## 37 - 38 0.0127 0.0266 6972 0.476 1.0000

## 37 - 39 0.0386 0.0253 6972 1.528 0.9734

## 37 - 40 0.0269 0.0247 6972 1.090 1.0000

## 37 - 41 0.0651 0.0256 6972 2.543 0.1539

## 37 - 42 0.0793 0.0318 6972 2.492 0.1638

## 38 - 39 0.0259 0.0183 6972 1.414 0.9734

## 38 - 40 0.0142 0.0175 6972 0.811 1.0000

## 38 - 41 0.0524 0.0188 6972 2.787 0.0800

## 38 - 42 0.0666 0.0267 6972 2.496 0.1638

## 39 - 40 -0.0117 0.0153 6972 -0.767 1.0000

## 39 - 41 0.0265 0.0168 6972 1.578 0.9734

## 39 - 42 0.0407 0.0253 6972 1.607 0.9734

## 40 - 41 0.0382 0.0159 6972 2.409 0.1761

## 40 - 42 0.0524 0.0247 6972 2.122 0.3391

## 41 - 42 0.0142 0.0256 6972 0.554 1.0000

##

## Results are given on the log (not the response) scale.

## P value adjustment: holm method for 15 tests

bio_gest(data=MFR_3742, bio=MFR_3742$VEGF)

## [[1]]

## Anova Table (Type III tests)

##

## Response: log(bio)

## Sum Sq Df F value Pr(>F)

## (Intercept) 1180.90 1 6050.8707 < 2.2e-16 ***

## AGE_AT_COLLECTION 50.92 1 260.9298 < 2.2e-16 ***

## ALDER_MODER 0.26 1 1.3491 0.245483

## BMI_MODER 0.56 1 2.8452 0.091692 .

## gestation_uge_f 3.15 5 3.2290 0.006483 **

## Residuals 1360.67 6972

## ---

## Signif. codes: 0 '***' 0.001 '**' 0.01 '*' 0.05 '.' 0.1 ' ' 1

##

## [[2]]

## [1] 0.03900837

##

## [[3]]

## contrast estimate SE df t.ratio p.value

## 37 - 38 0.00209 0.0256 6972 0.082 1.0000

## 37 - 39 0.03353 0.0242 6972 1.383 1.0000

## 37 - 40 0.03640 0.0237 6972 1.539 0.8670

## 37 - 41 0.06290 0.0245 6972 2.563 0.1458

## 37 - 42 0.06605 0.0305 6972 2.163 0.3665

## 38 - 39 0.03144 0.0176 6972 1.787 0.6812

## 38 - 40 0.03431 0.0168 6972 2.043 0.4518

## 38 - 41 0.06081 0.0180 6972 3.371 0.0113

## 38 - 42 0.06396 0.0256 6972 2.497 0.1629

## 39 - 40 0.00287 0.0147 6972 0.196 1.0000

## 39 - 41 0.02937 0.0161 6972 1.824 0.6812

## 39 - 42 0.03252 0.0243 6972 1.339 1.0000

## 40 - 41 0.02650 0.0152 6972 1.742 0.6812

## 40 - 42 0.02965 0.0237 6972 1.251 1.0000

## 41 - 42 0.00315 0.0246 6972 0.128 1.0000

##

## Results are given on the log (not the response) scale.

## P value adjustment: holm method for 15 tests

bio_gest(data=MFR_3742, bio=MFR_3742$S100B)

## [[1]]

## Anova Table (Type III tests)

##

## Response: log(bio)

## Sum Sq Df F value Pr(>F)

## (Intercept) 2303.90 1 7511.1058 < 2e-16 ***

## AGE_AT_COLLECTION 0.35 1 1.1389 0.28592

## ALDER_MODER 0.04 1 0.1319 0.71643

## BMI_MODER 0.06 1 0.2040 0.65152

## gestation_uge_f 4.36 5 2.8405 0.01444 *

## Residuals 2138.54 6972

## ---

## Signif. codes: 0 '***' 0.001 '**' 0.01 '*' 0.05 '.' 0.1 ' ' 1

##

## [[2]]

## [1] 0.002210052

##

## [[3]]

## contrast estimate SE df t.ratio p.value

## 37 - 38 0.0129 0.0320 6972 0.403 1.0000

## 37 - 39 0.0343 0.0304 6972 1.128 1.0000

## 37 - 40 0.0455 0.0297 6972 1.534 0.9740

## 37 - 41 0.0775 0.0308 6972 2.519 0.1651

## 37 - 42 0.0876 0.0383 6972 2.288 0.2663

## 38 - 39 0.0214 0.0221 6972 0.969 1.0000

## 38 - 40 0.0326 0.0211 6972 1.548 0.9740

## 38 - 41 0.0646 0.0226 6972 2.856 0.0645

## 38 - 42 0.0746 0.0321 6972 2.325 0.2616

## 39 - 40 0.0112 0.0184 6972 0.609 1.0000

## 39 - 41 0.0432 0.0202 6972 2.142 0.3547

## 39 - 42 0.0533 0.0305 6972 1.749 0.8037

## 40 - 41 0.0320 0.0191 6972 1.678 0.8399

## 40 - 42 0.0421 0.0297 6972 1.415 0.9740

## 41 - 42 0.0100 0.0308 6972 0.325 1.0000

##

## Results are given on the log (not the response) scale.

## P value adjustment: holm method for 15 tests

bio_gest(data=MFR_3742, bio=MFR_3742$BDNF)

## [[1]]

## Anova Table (Type III tests)

##

## Response: log(bio)

## Sum Sq Df F value Pr(>F)

## (Intercept) 2642.4 1 4805.4888 < 2.2e-16 ***

## AGE_AT_COLLECTION 5.7 1 10.3129 0.001327 **

## ALDER_MODER 4.7 1 8.5419 0.003482 **

## BMI_MODER 5.3 1 9.6684 0.001882 **

## gestation_uge_f 4.9 5 1.7814 0.112981

## Residuals 3833.7 6972

## ---

## Signif. codes: 0 '***' 0.001 '**' 0.01 '*' 0.05 '.' 0.1 ' ' 1

##

## [[2]]

## [1] 0.00548866

##

## [[3]]

## contrast estimate SE df t.ratio p.value

## 37 - 38 -0.06196 0.0429 6972 -1.444 1.0000

## 37 - 39 -0.03957 0.0407 6972 -0.973 1.0000

## 37 - 40 -0.07333 0.0397 6972 -1.847 0.8420

## 37 - 41 -0.03968 0.0412 6972 -0.963 1.0000

## 37 - 42 0.02461 0.0512 6972 0.480 1.0000

## 38 - 39 0.02238 0.0295 6972 0.758 1.0000

## 38 - 40 -0.01138 0.0282 6972 -0.404 1.0000

## 38 - 41 0.02227 0.0303 6972 0.736 1.0000

## 38 - 42 0.08656 0.0430 6972 2.014 0.6171

## 39 - 40 -0.03376 0.0246 6972 -1.370 1.0000

## 39 - 41 -0.00011 0.0270 6972 -0.004 1.0000

## 39 - 42 0.06418 0.0408 6972 1.574 1.0000

## 40 - 41 0.03365 0.0255 6972 1.318 1.0000

## 40 - 42 0.09794 0.0398 6972 2.461 0.2080

## 41 - 42 0.06429 0.0413 6972 1.557 1.0000

##

## Results are given on the log (not the response) scale.

## P value adjustment: holm method for 15 tests

bio_gest(data=MFR_3742, bio=MFR_3742$NT3)

## [[1]]

## Anova Table (Type III tests)

##

## Response: log(bio)

## Sum Sq Df F value Pr(>F)

## (Intercept) 142.25 1 591.3344 < 2.2e-16 ***

## AGE_AT_COLLECTION 4.73 1 19.6624 9.382e-06 ***

## ALDER_MODER 0.89 1 3.6956 0.05460 .

## BMI_MODER 0.66 1 2.7344 0.09825 .

## gestation_uge_f 1.17 5 0.9724 0.43303

## Residuals 1677.20 6972

## ---

## Signif. codes: 0 '***' 0.001 '**' 0.01 '*' 0.05 '.' 0.1 ' ' 1

##

## [[2]]

## [1] 0.00443751

##

## [[3]]

## contrast estimate SE df t.ratio p.value

## 37 - 38 -0.042391 0.0284 6972 -1.494 1.0000

## 37 - 39 -0.024960 0.0269 6972 -0.927 1.0000

## 37 - 40 -0.049346 0.0263 6972 -1.879 0.9040

## 37 - 41 -0.032549 0.0273 6972 -1.194 1.0000

## 37 - 42 -0.033011 0.0339 6972 -0.974 1.0000

## 38 - 39 0.017431 0.0195 6972 0.892 1.0000

## 38 - 40 -0.006956 0.0186 6972 -0.373 1.0000

## 38 - 41 0.009842 0.0200 6972 0.491 1.0000

## 38 - 42 0.009380 0.0284 6972 0.330 1.0000

## 39 - 40 -0.024387 0.0163 6972 -1.496 1.0000

## 39 - 41 -0.007589 0.0179 6972 -0.425 1.0000

## 39 - 42 -0.008051 0.0270 6972 -0.298 1.0000

## 40 - 41 0.016798 0.0169 6972 0.994 1.0000

## 40 - 42 0.016336 0.0263 6972 0.621 1.0000

## 41 - 42 -0.000462 0.0273 6972 -0.017 1.0000

##

## Results are given on the log (not the response) scale.

## P value adjustment: holm method for 15 tests
