# Supplementary material for: Delivery Modality Affect Neonatal Levels of Inflammation, Stress, and Growth Factors
Source: Front Pediatr. 2021 Sep 22;9:709765. doi: 10.3389/fped.2021.709765 (PMC8492985; doi:10.3389/fped.2021.709765)
Supplement: Supplementary file 1 [file Data_Sheet_1.zip › S2_Testing_other_variables.docx]

- S2.1 Loading data and adding variables
- S2.2 Age at sampling
  - S2.2.1 Anova to test if the age at sampling explains any of the variation in the biomarkers
  - S2.2.2 Testing association with GA
    - Figure A
  - S2.2.3 Testing association with birth type
    - Figure B
- S2.3 BMI of the mother
  - S2.3.1 Linear model to test if the BMI of the mother explains any of the variation in the biomarkers
  - S2.3.2 Testing association with GA:
    - Figure C
    - Figure D
  - S2.3.3 Testing association with birth type
    - Figure E
- S2.4 Maternal age
  - S2.4.1 Linear model to test if the maternal age explains any of the variation in the biomarkers
  - S2.4.2 Testing association with GA:
    - Figure F
    - Figure G
  - S2.4.3 Testing association with Birth type
    - Figure H
- S2.5 Birth weight
- S2.6 Association between type of anaesthetics and type of c-section
  - Figure I
  - Figure J

S2 Code for testing the influence of other variables on the biomarker levels, and their association with other variables

Testing the variables maternal age, BMI of the mother, age of sampling and weight at birth. Here we want to test, if any of these have influence on the level of the biomarker, and if it has, if there then is an association between it and the variables used in the analysis for the paper. If there is such associations a correction for the variables needs to be done to makes sure the variance found in the biomarker that are explained by the variable is not actually because of another variable (confounding factor).

S2.1 Loading data and adding variables

**library**(dplyr)

**library**(rcompanion)

**library**(ggplot2)

**library**(stringr)

**library**(tidyr)

**library**(data.table)

**library**(car)

**library**(ggpubr)

**library**(lsmeans)

**library**(FSA)

**library**(Hmisc)

**library**(Rmisc)

**library**("PerformanceAnalytics")

**library**(grid)

**library**(cowplot)

**library**(DBI)

**library**(rJava)

**library**(RJDBC)

**library**(vcd)

**library**(ggmosaic)

**library**(rstatix)

*#C-sections are defined by inlabor/prelabor instead of codes. Non-specific codes are defined by "Slet".*

MFR_kejsersnit <- rbind(MFR_KEJSERSNIT2009, MFR_KEJSERSNIT2010, MFR_KEJSERSNIT2011)

MFR_kejsersnit <- MFR_kejsersnit %>%

filter(SKSKODE %**in**% c("KMCA10B", "KMCA10E", "KMCA10A","KMCA10D" )) %>%

mutate(k_type = ifelse(SKSKODE == "KMCA10B", "Pre-labor",

ifelse(SKSKODE== "KMCA10E", "In-labor", "Slet"))) %>%

arrange(FK_MFR, k_type) %>%

distinct(FK_MFR, .keep_all=TRUE) %>%

data.table

*# Added to the other dataset*

MFR2 <- MFR_kejsersnit %>%

right_join(upload_MFR, by="FK_MFR") %>%

*# select(c("KODETYPE", "SKSKODE")) %>%*

data.table

*# Renaming variables with information that are understandable instead of codes*

MFR <- MFR2 %>%

mutate(tvilling = ifelse(str_detect(FLERFOLDSGRAVIDITET, "DO300"), "Ja", "Nej")) %>%

mutate(flerfold = ifelse(str_detect(FLERFOLDSGRAVIDITET, "DO30"), "Ja", "Nej")) %>%

mutate(PPROM = ifelse(PPROM=="DO422", "Ja", "Nej")) %>%

mutate(sepsis = ifelse(str_detect(SEPSIS_BARN, "DP36"), "Ja", "Nej")) %>%

replace_na(list(tvilling="Nej")) %>%

replace_na(list(flerfold="Nej")) %>%

replace_na(list(PPROM="Nej")) %>%

replace_na(list(sepsis="Nej")) %>%

replace_na(list(k_type="Vaginal")) %>%

mutate(CRP = CRP*1e-06) %>%

mutate(gestation_uge_f = as.factor(gestation_uge)) %>%

filter(gestation_uge > 23 & gestation_uge < 43) %>%

filter(k_type != "Slet") %>%

data.table

MFR <- MFR %>%

mutate(Birth_type = k_type) %>%

mutate(Gender = KOEN_BARN) %>%

data.table

*# Filtering so we only have the children born on time*

MFR_3742 <- MFR %>%

filter(gestation_uge > 36 & gestation_uge < 43) %>%

data.table

MFR_3742$gest <- cut(MFR_3742$gestation_uge, c(36, 37, 38, 39, 42))

MFR_3742 <- MFR_3742 %>%

mutate(Gender = ifelse(KOEN_BARN=="M", "Boys", "Girls")) %>%

data.table

rm(MFR_kejsersnit, MFR_KEJSERSNIT2009, MFR_KEJSERSNIT2010, MFR_KEJSERSNIT2011, MFR2)

*# Log transformation of all biomarkers*

MFR_3742$logCRP <- log(MFR_3742$CRP)

MFR_3742$logIL18 <- log(MFR_3742$IL18)

MFR_3742$logMCP1 <- log(MFR_3742$MCP1)

MFR_3742$logHSP70 <- log(MFR_3742$HSP70)

MFR_3742$logSTNF_RI <- log(MFR_3742$STNF_RI)

MFR_3742$logEGF <- log(MFR_3742$EGF)

MFR_3742$logBDNF <- log(MFR_3742$BDNF)

MFR_3742$logNT3 <- log(MFR_3742$NT3)

MFR_3742$logS100B <- log(MFR_3742$S100B)

MFR_3742$logVEGF <- log(MFR_3742$VEGF)

MFR_3742$gestation_uge_f <- as.factor(MFR_3742$gestation_uge)

MFR_3742 <- MFR_3742 %>%

filter(Birth_type != "Slet") %>%

data.table

S2.2 Age at sampling

S2.2.1 Anova to test if the age at sampling explains any of the variation in the biomarkers

MFR_3742$AGE_AT_COLLECTION <- as.factor(MFR_3742$AGE_AT_COLLECTION)

model <- aov(logCRP ~ AGE_AT_COLLECTION, data=MFR_3742)

Anova(model, type="III")

## Anova Table (Type III tests)

##

## Response: logCRP

## Sum Sq Df F value Pr(>F)

## (Intercept) 4423.0 1 7671.987 < 2.2e-16 ***

## AGE_AT_COLLECTION 19.9 2 17.265 3.31e-08 ***

## Residuals 4187.2 7263

## ---

## Signif. codes: 0 '***' 0.001 '**' 0.01 '*' 0.05 '.' 0.1 ' ' 1

model <- aov(logMCP1 ~ AGE_AT_COLLECTION, data=MFR_3742)

Anova(model, type="III")

## Anova Table (Type III tests)

##

## Response: logMCP1

## Sum Sq Df F value Pr(>F)

## (Intercept) 106034 1 561172.89 < 2.2e-16 ***

## AGE_AT_COLLECTION 49 2 130.86 < 2.2e-16 ***

## Residuals 1372 7263

## ---

## Signif. codes: 0 '***' 0.001 '**' 0.01 '*' 0.05 '.' 0.1 ' ' 1

model <- aov(logIL18 ~ AGE_AT_COLLECTION, data=MFR_3742)

Anova(model, type="III")

## Anova Table (Type III tests)

##

## Response: logIL18

## Sum Sq Df F value Pr(>F)

## (Intercept) 36584 1 2.0101e+05 <2e-16 ***

## AGE_AT_COLLECTION 0 2 3.2720e-01 0.721

## Residuals 1322 7263

## ---

## Signif. codes: 0 '***' 0.001 '**' 0.01 '*' 0.05 '.' 0.1 ' ' 1

model <- aov(logHSP70 ~ AGE_AT_COLLECTION, data=MFR_3742)

Anova(model, type="III")

## Anova Table (Type III tests)

##

## Response: logHSP70

## Sum Sq Df F value Pr(>F)

## (Intercept) 516651 1 5.5227e+06 <2e-16 ***

## AGE_AT_COLLECTION 0 2 2.4360e-01 0.7838

## Residuals 679 7263

## ---

## Signif. codes: 0 '***' 0.001 '**' 0.01 '*' 0.05 '.' 0.1 ' ' 1

model <- aov(logSTNF_RI ~ AGE_AT_COLLECTION, data=MFR_3742)

Anova(model, type="III")

## Anova Table (Type III tests)

##

## Response: logSTNF_RI

## Sum Sq Df F value Pr(>F)

## (Intercept) 70505 1 245922.623 < 2.2e-16 ***

## AGE_AT_COLLECTION 44 2 76.563 < 2.2e-16 ***

## Residuals 2082 7263

## ---

## Signif. codes: 0 '***' 0.001 '**' 0.01 '*' 0.05 '.' 0.1 ' ' 1

model <- aov(logEGF ~ AGE_AT_COLLECTION, data=MFR_3742)

Anova(model, type="III")

## Anova Table (Type III tests)

##

## Response: logEGF

## Sum Sq Df F value Pr(>F)

## (Intercept) 29890.8 1 140407.548 < 2.2e-16 ***

## AGE_AT_COLLECTION 3.6 2 8.534 0.0001986 ***

## Residuals 1546.2 7263

## ---

## Signif. codes: 0 '***' 0.001 '**' 0.01 '*' 0.05 '.' 0.1 ' ' 1

model <- aov(logVEGF ~ AGE_AT_COLLECTION, data=MFR_3742)

Anova(model, type="III")

## Anova Table (Type III tests)

##

## Response: logVEGF

## Sum Sq Df F value Pr(>F)

## (Intercept) 46817 1 239785.17 < 2.2e-16 ***

## AGE_AT_COLLECTION 52 2 133.02 < 2.2e-16 ***

## Residuals 1418 7263

## ---

## Signif. codes: 0 '***' 0.001 '**' 0.01 '*' 0.05 '.' 0.1 ' ' 1

model <- aov(logS100B ~ AGE_AT_COLLECTION, data=MFR_3742)

Anova(model, type="III")

## Anova Table (Type III tests)

##

## Response: logS100B

## Sum Sq Df F value Pr(>F)

## (Intercept) 113801 1 3.6769e+05 < 2e-16 ***

## AGE_AT_COLLECTION 2 2 2.6858e+00 0.06823 .

## Residuals 2248 7263

## ---

## Signif. codes: 0 '***' 0.001 '**' 0.01 '*' 0.05 '.' 0.1 ' ' 1

model <- aov(logBDNF ~ AGE_AT_COLLECTION, data=MFR_3742)

Anova(model, type="III")

## Anova Table (Type III tests)

##

## Response: logBDNF

## Sum Sq Df F value Pr(>F)

## (Intercept) 117973 1 2.1332e+05 < 2.2e-16 ***

## AGE_AT_COLLECTION 6 2 5.1023e+00 0.006104 **

## Residuals 4017 7263

## ---

## Signif. codes: 0 '***' 0.001 '**' 0.01 '*' 0.05 '.' 0.1 ' ' 1

model <- aov(logNT3 ~ AGE_AT_COLLECTION, data=MFR_3742)

Anova(model, type="III")

## Anova Table (Type III tests)

##

## Response: logNT3

## Sum Sq Df F value Pr(>F)

## (Intercept) 5498.4 1 22689.267 < 2.2e-16 ***

## AGE_AT_COLLECTION 5.1 2 10.553 2.653e-05 ***

## Residuals 1760.1 7263

## ---

## Signif. codes: 0 '***' 0.001 '**' 0.01 '*' 0.05 '.' 0.1 ' ' 1

It is significant for multiple biomarkers, therefore it is tested, if has an association with GA or birth type (we do not expect it to have any association with gender)

S2.2.2 Testing association with GA

Using a *chi*^2^ test, since both are categorical

table(MFR_3742$AGE_AT_COLLECTION, MFR_3742$gestation_uge_f)

##

## 37 38 39 40 41 42

## 2 205 601 954 1156 755 210

## 3 225 454 687 963 688 219

## 4 10 25 36 41 30 7

chisq.test(table(MFR_3742$AGE_AT_COLLECTION, MFR_3742$gestation_uge_f))

##

## Pearson's Chi-squared test

##

## data: table(MFR_3742$AGE_AT_COLLECTION, MFR_3742$gestation_uge_f)

## X-squared = 28.737, df = 10, p-value = 0.001374

Figure A

MFR_3742 %>% ggplot()+

geom_mosaic(aes(fill=AGE_AT_COLLECTION, x=product(gestation_uge_f)))+

labs(x="GA (weeks)", y="Age at collection", fill="Age at collection")+

theme_minimal()


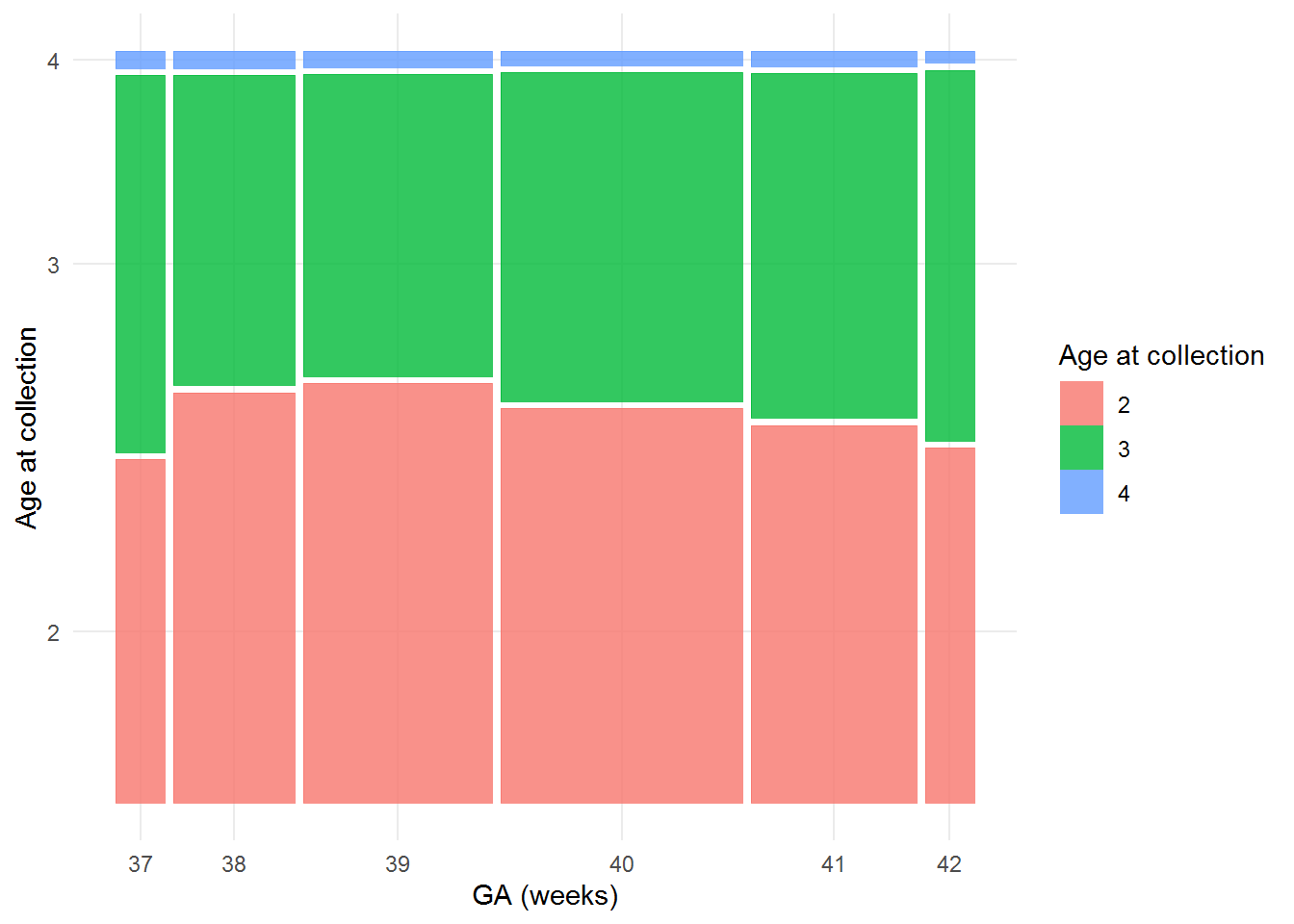


There is an association, which means we have to correct for the age at collection in our analysis to be sure it is not a confounding factor.

S2.2.3 Testing association with birth type

Again a *chi*^2^ test can be used

table(MFR_3742$AGE_AT_COLLECTION, MFR_3742$Birth_type)

##

## In-labor Pre-labor Vaginal

## 2 316 504 3061

## 3 320 203 2713

## 4 19 7 123

chisq.test(table(MFR_3742$AGE_AT_COLLECTION, MFR_3742$Birth_type))

##

## Pearson's Chi-squared test

##

## data: table(MFR_3742$AGE_AT_COLLECTION, MFR_3742$Birth_type)

## X-squared = 98.729, df = 4, p-value < 2.2e-16

Figure B

MFR_3742 %>% ggplot()+

geom_mosaic(aes(fill=AGE_AT_COLLECTION, x=product(Birth_type)))+

labs(x="Birth type", y="Age at collection", fill="Age at collection")+

theme_minimal()+

theme(axis.text.x = element_text(angle=45))+

NULL


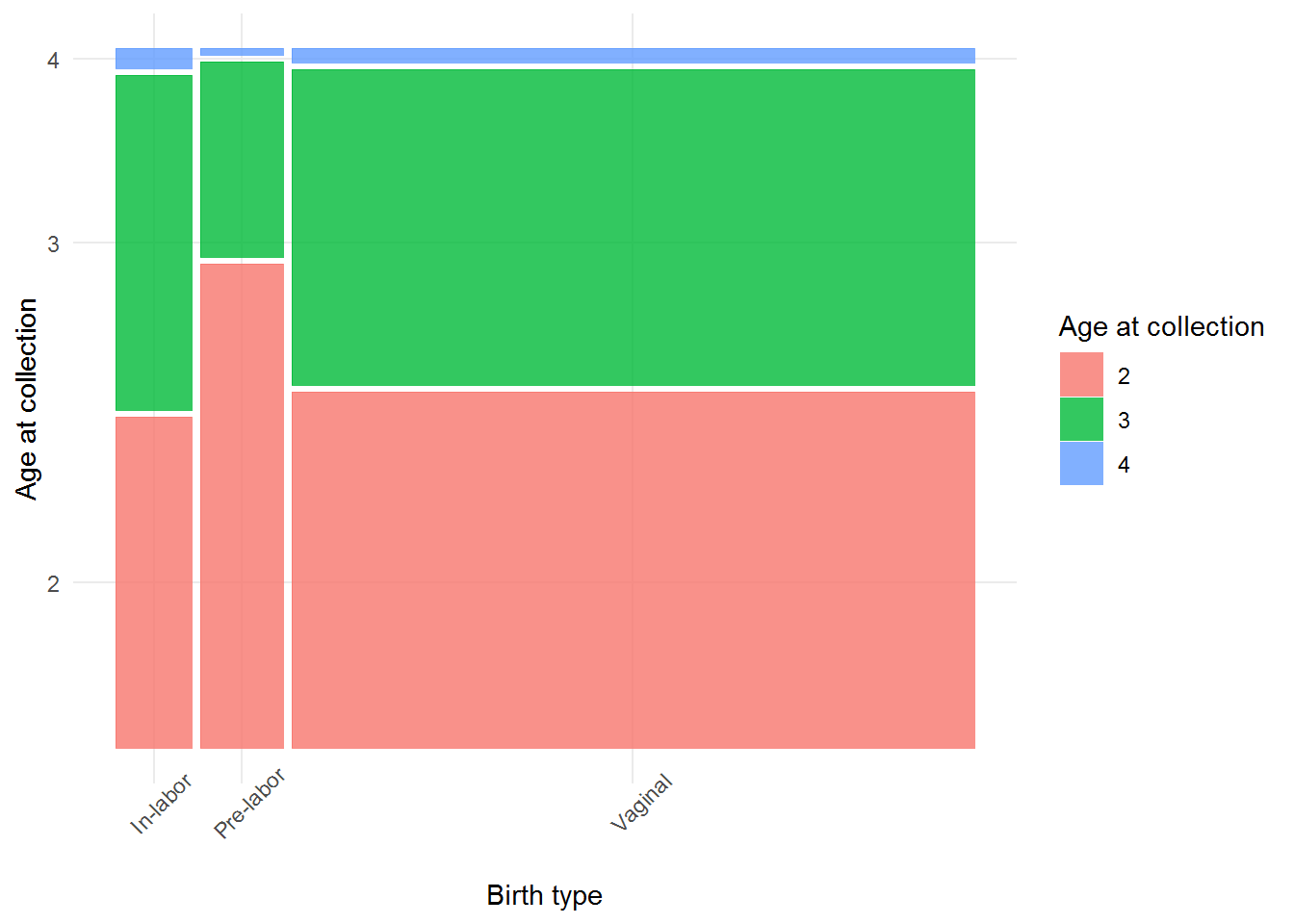


This is again siginicant (this time very significant), and it supports the suggestion that we have to correct for age at sampling.

Correction is done in the main analyses.

S2.3 BMI of the mother

First we have to remove the cases, that are clearly wrong (below 30 and above 300 kg, and below 140 and above 250 cm):

BMI <- MFR_3742 %>% filter(VAEGT_MODER > 30 & VAEGT_MODER < 300) %>% filter(HOEJDE_MODER > 140 & HOEJDE_MODER < 250)

S2.3.1 Linear model to test if the BMI of the mother explains any of the variation in the biomarkers

Here we use a linear model instead of anova, since the BMI of the mother is numerical, not categorical.

model <- lm(logCRP ~ BMI_MODER, data=MFR_3742)

summary(model)

##

## Call:

## lm(formula = logCRP ~ BMI_MODER, data = MFR_3742)

##

## Residuals:

## Min 1Q Median 3Q Max

## -7.8037 -0.4650 -0.0024 0.4487 3.4230

##

## Coefficients:

## Estimate Std. Error t value Pr(>|t|)

## (Intercept) -1.1203784 0.0300317 -37.307 <2e-16 ***

## BMI_MODER 0.0009558 0.0011688 0.818 0.414

## ---

## Signif. codes: 0 '***' 0.001 '**' 0.01 '*' 0.05 '.' 0.1 ' ' 1

##

## Residual standard error: 0.7631 on 6979 degrees of freedom

## (285 observations deleted due to missingness)

## Multiple R-squared: 9.58e-05, Adjusted R-squared: -4.747e-05

## F-statistic: 0.6686 on 1 and 6979 DF, p-value: 0.4136

model <- lm(logMCP1 ~ BMI_MODER, data=MFR_3742)

summary(model)

##

## Call:

## lm(formula = logMCP1 ~ BMI_MODER, data = MFR_3742)

##

## Residuals:

## Min 1Q Median 3Q Max

## -2.32615 -0.28044 0.00555 0.28145 1.93790

##

## Coefficients:

## Estimate Std. Error t value Pr(>|t|)

## (Intercept) 5.1657857 0.0173675 297.440 <2e-16 ***

## BMI_MODER -0.0005021 0.0006760 -0.743 0.458

## ---

## Signif. codes: 0 '***' 0.001 '**' 0.01 '*' 0.05 '.' 0.1 ' ' 1

##

## Residual standard error: 0.4413 on 6979 degrees of freedom

## (285 observations deleted due to missingness)

## Multiple R-squared: 7.905e-05, Adjusted R-squared: -6.423e-05

## F-statistic: 0.5517 on 1 and 6979 DF, p-value: 0.4576

model <- lm(logIL18 ~ BMI_MODER, data=MFR_3742)

summary(model)

##

## Call:

## lm(formula = logIL18 ~ BMI_MODER, data = MFR_3742)

##

## Residuals:

## Min 1Q Median 3Q Max

## -2.46083 -0.28061 0.00327 0.27587 1.99616

##

## Coefficients:

## Estimate Std. Error t value Pr(>|t|)

## (Intercept) 3.0456966 0.0167292 182.06 <2e-16 ***

## BMI_MODER 0.0010287 0.0006511 1.58 0.114

## ---

## Signif. codes: 0 '***' 0.001 '**' 0.01 '*' 0.05 '.' 0.1 ' ' 1

##

## Residual standard error: 0.4251 on 6979 degrees of freedom

## (285 observations deleted due to missingness)

## Multiple R-squared: 0.0003575, Adjusted R-squared: 0.0002143

## F-statistic: 2.496 on 1 and 6979 DF, p-value: 0.1142

model <- lm(logHSP70 ~ BMI_MODER, data=MFR_3742)

summary(model)

##

## Call:

## lm(formula = logHSP70 ~ BMI_MODER, data = MFR_3742)

##

## Residuals:

## Min 1Q Median 3Q Max

## -6.0325 -0.1838 0.0053 0.1875 1.7732

##

## Coefficients:

## Estimate Std. Error t value Pr(>|t|)

## (Intercept) 1.151e+01 1.202e-02 957.291 <2e-16 ***

## BMI_MODER 1.176e-03 4.679e-04 2.512 0.012 *

## ---

## Signif. codes: 0 '***' 0.001 '**' 0.01 '*' 0.05 '.' 0.1 ' ' 1

##

## Residual standard error: 0.3055 on 6979 degrees of freedom

## (285 observations deleted due to missingness)

## Multiple R-squared: 0.0009035, Adjusted R-squared: 0.0007604

## F-statistic: 6.311 on 1 and 6979 DF, p-value: 0.01202

model <- lm(logSTNF_RI ~ BMI_MODER, data=MFR_3742)

summary(model)

##

## Call:

## lm(formula = logSTNF_RI ~ BMI_MODER, data = MFR_3742)

##

## Residuals:

## Min 1Q Median 3Q Max

## -1.4251 -0.2774 0.0830 0.3575 3.3201

##

## Coefficients:

## Estimate Std. Error t value Pr(>|t|)

## (Intercept) 4.2512392 0.0212792 199.784 < 2e-16 ***

## BMI_MODER -0.0024815 0.0008282 -2.996 0.00274 **

## ---

## Signif. codes: 0 '***' 0.001 '**' 0.01 '*' 0.05 '.' 0.1 ' ' 1

##

## Residual standard error: 0.5407 on 6979 degrees of freedom

## (285 observations deleted due to missingness)

## Multiple R-squared: 0.001285, Adjusted R-squared: 0.001142

## F-statistic: 8.977 on 1 and 6979 DF, p-value: 0.002743

model <- lm(logEGF ~ BMI_MODER, data=MFR_3742)

summary(model)

##

## Call:

## lm(formula = logEGF ~ BMI_MODER, data = MFR_3742)

##

## Residuals:

## Min 1Q Median 3Q Max

## -1.8358 -0.2722 0.0153 0.2754 3.6532

##

## Coefficients:

## Estimate Std. Error t value Pr(>|t|)

## (Intercept) 2.7857430 0.0181513 153.474 <2e-16 ***

## BMI_MODER -0.0012732 0.0007065 -1.802 0.0716 .

## ---

## Signif. codes: 0 '***' 0.001 '**' 0.01 '*' 0.05 '.' 0.1 ' ' 1

##

## Residual standard error: 0.4612 on 6979 degrees of freedom

## (285 observations deleted due to missingness)

## Multiple R-squared: 0.0004652, Adjusted R-squared: 0.000322

## F-statistic: 3.248 on 1 and 6979 DF, p-value: 0.07155

model <- lm(logVEGF ~ BMI_MODER, data=MFR_3742)

summary(model)

##

## Call:

## lm(formula = logVEGF ~ BMI_MODER, data = MFR_3742)

##

## Residuals:

## Min 1Q Median 3Q Max

## -2.30855 -0.28970 0.01119 0.31037 1.76423

##

## Coefficients:

## Estimate Std. Error t value Pr(>|t|)

## (Intercept) 3.4222917 0.0177229 193.100 <2e-16 ***

## BMI_MODER -0.0011637 0.0006898 -1.687 0.0917 .

## ---

## Signif. codes: 0 '***' 0.001 '**' 0.01 '*' 0.05 '.' 0.1 ' ' 1

##

## Residual standard error: 0.4503 on 6979 degrees of freedom

## (285 observations deleted due to missingness)

## Multiple R-squared: 0.0004076, Adjusted R-squared: 0.0002644

## F-statistic: 2.846 on 1 and 6979 DF, p-value: 0.09165

model <- lm(logS100B ~ BMI_MODER, data=MFR_3742)

summary(model)

##

## Call:

## lm(formula = logS100B ~ BMI_MODER, data = MFR_3742)

##

## Residuals:

## Min 1Q Median 3Q Max

## -2.06036 -0.36660 0.00425 0.35971 2.84362

##

## Coefficients:

## Estimate Std. Error t value Pr(>|t|)

## (Intercept) 5.4106576 0.0218094 248.089 <2e-16 ***

## BMI_MODER 0.0003034 0.0008488 0.357 0.721

## ---

## Signif. codes: 0 '***' 0.001 '**' 0.01 '*' 0.05 '.' 0.1 ' ' 1

##

## Residual standard error: 0.5542 on 6979 degrees of freedom

## (285 observations deleted due to missingness)

## Multiple R-squared: 1.831e-05, Adjusted R-squared: -0.000125

## F-statistic: 0.1278 on 1 and 6979 DF, p-value: 0.7207

model <- lm(logBDNF ~ BMI_MODER, data=MFR_3742)

summary(model)

##

## Call:

## lm(formula = logBDNF ~ BMI_MODER, data = MFR_3742)

##

## Residuals:

## Min 1Q Median 3Q Max

## -1.4593 -0.4462 0.0710 0.4756 4.8499

##

## Coefficients:

## Estimate Std. Error t value Pr(>|t|)

## (Intercept) 5.577511 0.029228 190.830 < 2e-16 ***

## BMI_MODER -0.003639 0.001138 -3.199 0.00138 **

## ---

## Signif. codes: 0 '***' 0.001 '**' 0.01 '*' 0.05 '.' 0.1 ' ' 1

##

## Residual standard error: 0.7427 on 6979 degrees of freedom

## (285 observations deleted due to missingness)

## Multiple R-squared: 0.001464, Adjusted R-squared: 0.001321

## F-statistic: 10.23 on 1 and 6979 DF, p-value: 0.001385

model <- lm(logNT3 ~ BMI_MODER, data=MFR_3742)

summary(model)

##

## Call:

## lm(formula = logNT3 ~ BMI_MODER, data = MFR_3742)

##

## Residuals:

## Min 1Q Median 3Q Max

## -0.4973 -0.4484 -0.0772 0.2607 5.4188

##

## Coefficients:

## Estimate Std. Error t value Pr(>|t|)

## (Intercept) 1.1954603 0.0193322 61.838 <2e-16 ***

## BMI_MODER -0.0012446 0.0007524 -1.654 0.0982 .

## ---

## Signif. codes: 0 '***' 0.001 '**' 0.01 '*' 0.05 '.' 0.1 ' ' 1

##

## Residual standard error: 0.4912 on 6979 degrees of freedom

## (285 observations deleted due to missingness)

## Multiple R-squared: 0.0003919, Adjusted R-squared: 0.0002486

## F-statistic: 2.736 on 1 and 6979 DF, p-value: 0.09816

It is significant for multiple biomarkers, therefore it is tested, if has an association with GA or birth type (we do not expect it to have any association with gender)

S2.3.2 Testing association with GA:

model <- aov(BMI_MODER ~ gestation_uge_f, data=MFR_3742)

Anova(model, type="III")

## Anova Table (Type III tests)

##

## Response: BMI_MODER

## Sum Sq Df F value Pr(>F)

## (Intercept) 259543 1 4257.4814 < 2.2e-16 ***

## gestation_uge_f 1013 5 3.3229 0.005328 **

## Residuals 425207 6975

## ---

## Signif. codes: 0 '***' 0.001 '**' 0.01 '*' 0.05 '.' 0.1 ' ' 1

Here an association is found, and we have to correct for it

*#Adding categories:*

BMI <- MFR_3742 %>% mutate(BMI_category= case_when(BMI_MODER<18.5 ~ "Underweight",

BMI_MODER>18.4 & BMI_MODER<25 ~ "Normal",

BMI_MODER>24.9 & BMI_MODER<30 ~ "Overweight",

BMI_MODER>=30 ~ "Fat")) %>% filter(!is.na(BMI_category))

BMI$BMI_category <- factor(BMI$BMI_category, levels=c("Underweight", "Normal", "Overweight", "Fat"))

Figure C

*#Plotting:*

BMI %>% ggplot()+

geom_mosaic(aes(x=product(BMI_category), fill=gestation_uge_f))+

labs(x="BMI category", y="GA (weeks)", fill="GA (weeks)")+

theme_minimal()


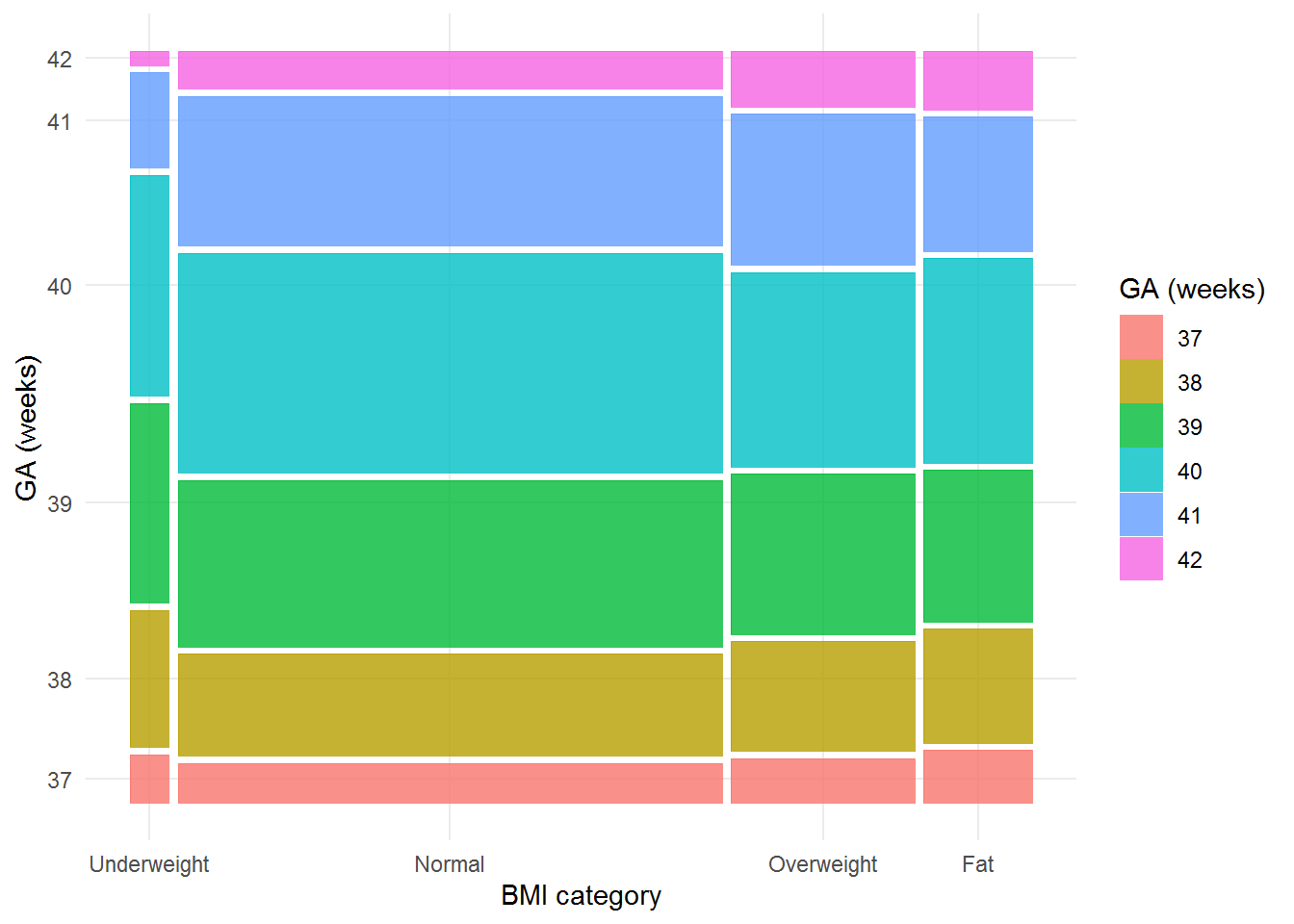


Figure D

BMI %>% ggplot()+

geom_mosaic(aes(x=product(gestation_uge_f), fill=BMI_category))+

labs(x="GA (weeks)", y="BMI category", fill="BMI category")+

theme_minimal()


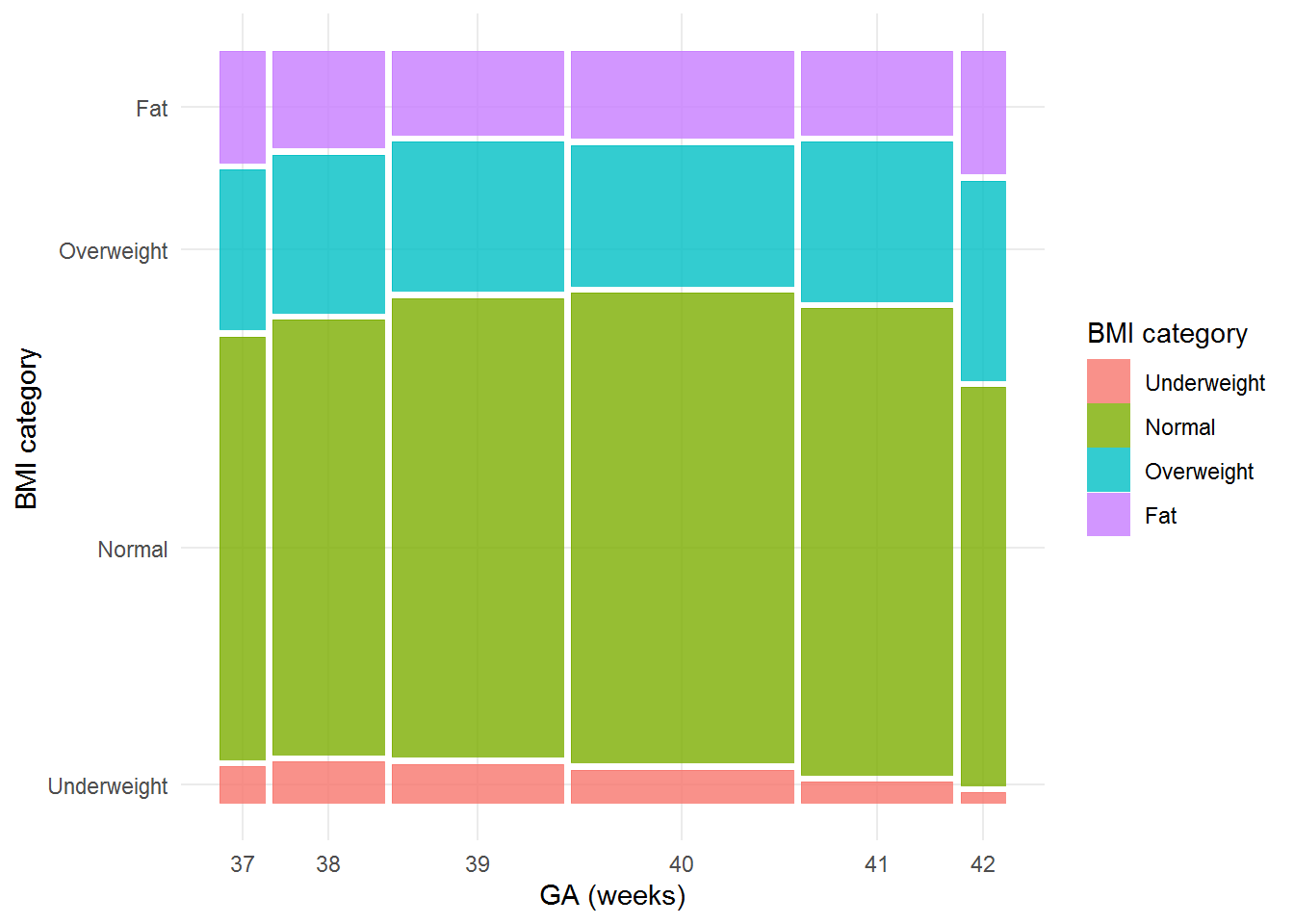


S2.3.3 Testing association with birth type

model <- aov(BMI_MODER ~ Birth_type, data=MFR_3742)

Anova(model, type="III")

## Anova Table (Type III tests)

##

## Response: BMI_MODER

## Sum Sq Df F value Pr(>F)

## (Intercept) 411389 1 6764.114 < 2.2e-16 ***

## Birth_type 1823 2 14.983 3.212e-07 ***

## Residuals 424397 6978

## ---

## Signif. codes: 0 '***' 0.001 '**' 0.01 '*' 0.05 '.' 0.1 ' ' 1

This is significant as well, and it supports the suggestion that we have to correct for the BMI of the mother.

Correction is done in the main analyses.

Figure E

*#Plotting:*

BMI %>% ggplot()+

geom_mosaic(aes(x=product(BMI_category), fill=Birth_type))+

labs(x="BMI category", y="Birth type", fill="Birth type")+

theme_minimal()


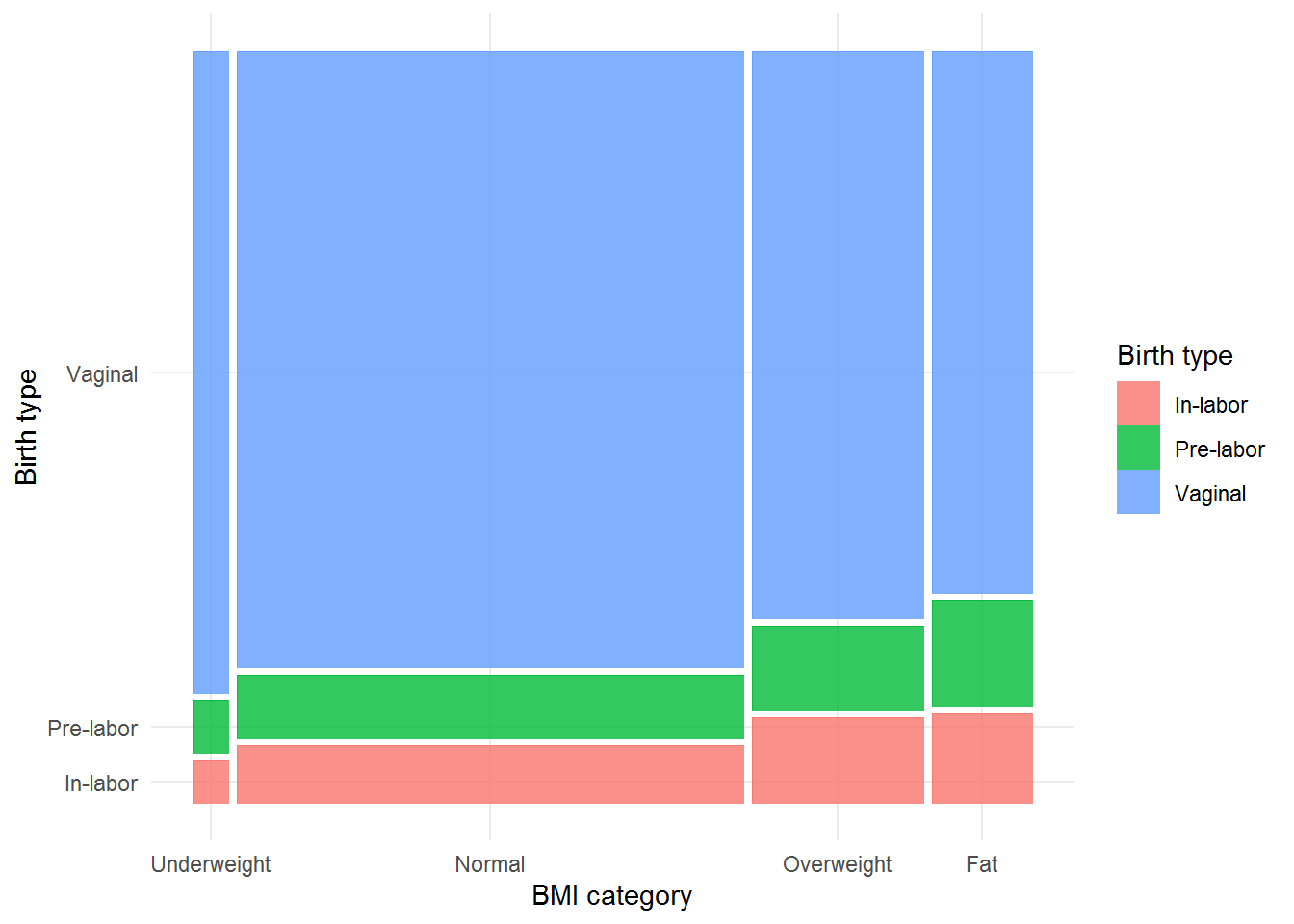


rm(BMI)

S2.4 Maternal age

S2.4.1 Linear model to test if the maternal age explains any of the variation in the biomarkers

Here we use a linear model instead of anova, since the maternal age is numerical, not categorical.

summary(lm(logCRP ~ ALDER_MODER, data=MFR_3742))

##

## Call:

## lm(formula = logCRP ~ ALDER_MODER, data = MFR_3742)

##

## Residuals:

## Min 1Q Median 3Q Max

## -7.7395 -0.4631 -0.0013 0.4494 3.4393

##

## Coefficients:

## Estimate Std. Error t value Pr(>|t|)

## (Intercept) -0.851066 0.055682 -15.285 < 2e-16 ***

## ALDER_MODER -0.008160 0.001803 -4.526 6.11e-06 ***

## ---

## Signif. codes: 0 '***' 0.001 '**' 0.01 '*' 0.05 '.' 0.1 ' ' 1

##

## Residual standard error: 0.76 on 7264 degrees of freedom

## Multiple R-squared: 0.002812, Adjusted R-squared: 0.002674

## F-statistic: 20.48 on 1 and 7264 DF, p-value: 6.115e-06

summary(lm(logMCP1 ~ ALDER_MODER, data=MFR_3742))

##

## Call:

## lm(formula = logMCP1 ~ ALDER_MODER, data = MFR_3742)

##

## Residuals:

## Min 1Q Median 3Q Max

## -2.37655 -0.28228 0.00477 0.28040 1.93641

##

## Coefficients:

## Estimate Std. Error t value Pr(>|t|)

## (Intercept) 5.228076 0.032403 161.347 <2e-16 ***

## ALDER_MODER -0.002486 0.001049 -2.369 0.0179 *

## ---

## Signif. codes: 0 '***' 0.001 '**' 0.01 '*' 0.05 '.' 0.1 ' ' 1

##

## Residual standard error: 0.4422 on 7264 degrees of freedom

## Multiple R-squared: 0.0007721, Adjusted R-squared: 0.0006345

## F-statistic: 5.613 on 1 and 7264 DF, p-value: 0.01786

summary(lm(logIL18 ~ ALDER_MODER, data=MFR_3742))

##

## Call:

## lm(formula = logIL18 ~ ALDER_MODER, data = MFR_3742)

##

## Residuals:

## Min 1Q Median 3Q Max

## -2.46856 -0.28200 0.00284 0.27514 1.99055

##

## Coefficients:

## Estimate Std. Error t value Pr(>|t|)

## (Intercept) 3.071e+00 3.126e-02 98.25 <2e-16 ***

## ALDER_MODER 7.132e-05 1.012e-03 0.07 0.944

## ---

## Signif. codes: 0 '***' 0.001 '**' 0.01 '*' 0.05 '.' 0.1 ' ' 1

##

## Residual standard error: 0.4266 on 7264 degrees of freedom

## Multiple R-squared: 6.834e-07, Adjusted R-squared: -0.000137

## F-statistic: 0.004964 on 1 and 7264 DF, p-value: 0.9438

summary(lm(logHSP70 ~ ALDER_MODER, data=MFR_3742))

##

## Call:

## lm(formula = logHSP70 ~ ALDER_MODER, data = MFR_3742)

##

## Residuals:

## Min 1Q Median 3Q Max

## -6.0276 -0.1829 0.0035 0.1890 1.7732

##

## Coefficients:

## Estimate Std. Error t value Pr(>|t|)

## (Intercept) 11.5622578 0.0224074 516.001 <2e-16 ***

## ALDER_MODER -0.0007644 0.0007256 -1.053 0.292

## ---

## Signif. codes: 0 '***' 0.001 '**' 0.01 '*' 0.05 '.' 0.1 ' ' 1

##

## Residual standard error: 0.3058 on 7264 degrees of freedom

## Multiple R-squared: 0.0001528, Adjusted R-squared: 1.511e-05

## F-statistic: 1.11 on 1 and 7264 DF, p-value: 0.2922

summary(lm(logSTNF_RI ~ ALDER_MODER, data=MFR_3742))

##

## Call:

## lm(formula = logSTNF_RI ~ ALDER_MODER, data = MFR_3742)

##

## Residuals:

## Min 1Q Median 3Q Max

## -1.4128 -0.2724 0.0824 0.3572 3.3249

##

## Coefficients:

## Estimate Std. Error t value Pr(>|t|)

## (Intercept) 4.300101 0.039618 108.539 < 2e-16 ***

## ALDER_MODER -0.003601 0.001283 -2.807 0.00501 **

## ---

## Signif. codes: 0 '***' 0.001 '**' 0.01 '*' 0.05 '.' 0.1 ' ' 1

##

## Residual standard error: 0.5407 on 7264 degrees of freedom

## Multiple R-squared: 0.001084, Adjusted R-squared: 0.000946

## F-statistic: 7.879 on 1 and 7264 DF, p-value: 0.005014

summary(lm(logEGF ~ ALDER_MODER, data=MFR_3742))

##

## Call:

## lm(formula = logEGF ~ ALDER_MODER, data = MFR_3742)

##

## Residuals:

## Min 1Q Median 3Q Max

## -1.8358 -0.2754 0.0164 0.2758 3.6580

##

## Coefficients:

## Estimate Std. Error t value Pr(>|t|)

## (Intercept) 2.802705 0.033839 82.826 <2e-16 ***

## ALDER_MODER -0.001547 0.001096 -1.412 0.158

## ---

## Signif. codes: 0 '***' 0.001 '**' 0.01 '*' 0.05 '.' 0.1 ' ' 1

##

## Residual standard error: 0.4618 on 7264 degrees of freedom

## Multiple R-squared: 0.0002743, Adjusted R-squared: 0.0001367

## F-statistic: 1.993 on 1 and 7264 DF, p-value: 0.158

summary(lm(logVEGF ~ ALDER_MODER, data=MFR_3742))

##

## Call:

## lm(formula = logVEGF ~ ALDER_MODER, data = MFR_3742)

##

## Residuals:

## Min 1Q Median 3Q Max

## -2.30318 -0.28868 0.00966 0.30840 1.76393

##

## Coefficients:

## Estimate Std. Error t value Pr(>|t|)

## (Intercept) 3.4206017 0.0329587 103.784 <2e-16 ***

## ALDER_MODER -0.0008483 0.0010673 -0.795 0.427

## ---

## Signif. codes: 0 '***' 0.001 '**' 0.01 '*' 0.05 '.' 0.1 ' ' 1

##

## Residual standard error: 0.4498 on 7264 degrees of freedom

## Multiple R-squared: 8.696e-05, Adjusted R-squared: -5.069e-05

## F-statistic: 0.6317 on 1 and 7264 DF, p-value: 0.4267

summary(lm(logS100B ~ ALDER_MODER, data=MFR_3742))

##

## Call:

## lm(formula = logS100B ~ ALDER_MODER, data = MFR_3742)

##

## Residuals:

## Min 1Q Median 3Q Max

## -2.07042 -0.36798 0.00352 0.36091 2.84647

##

## Coefficients:

## Estimate Std. Error t value Pr(>|t|)

## (Intercept) 5.4479190 0.0407723 133.618 <2e-16 ***

## ALDER_MODER -0.0009258 0.0013203 -0.701 0.483

## ---

## Signif. codes: 0 '***' 0.001 '**' 0.01 '*' 0.05 '.' 0.1 ' ' 1

##

## Residual standard error: 0.5565 on 7264 degrees of freedom

## Multiple R-squared: 6.768e-05, Adjusted R-squared: -6.998e-05

## F-statistic: 0.4916 on 1 and 7264 DF, p-value: 0.4832

summary(lm(logBDNF ~ ALDER_MODER, data=MFR_3742))

##

## Call:

## lm(formula = logBDNF ~ ALDER_MODER, data = MFR_3742)

##

## Residuals:

## Min 1Q Median 3Q Max

## -1.4366 -0.4451 0.0709 0.4764 4.8343

##

## Coefficients:

## Estimate Std. Error t value Pr(>|t|)

## (Intercept) 5.639616 0.054492 103.494 < 2e-16 ***

## ALDER_MODER -0.004985 0.001765 -2.825 0.00474 **

## ---

## Signif. codes: 0 '***' 0.001 '**' 0.01 '*' 0.05 '.' 0.1 ' ' 1

##

## Residual standard error: 0.7437 on 7264 degrees of freedom

## Multiple R-squared: 0.001098, Adjusted R-squared: 0.0009601

## F-statistic: 7.982 on 1 and 7264 DF, p-value: 0.004738

summary(lm(logNT3 ~ ALDER_MODER, data=MFR_3742))

##

## Call:

## lm(formula = logNT3 ~ ALDER_MODER, data = MFR_3742)

##

## Residuals:

## Min 1Q Median 3Q Max

## -0.5010 -0.4452 -0.0753 0.2615 5.4276

##

## Coefficients:

## Estimate Std. Error t value Pr(>|t|)

## (Intercept) 1.236395 0.036108 34.241 <2e-16 ***

## ALDER_MODER -0.002326 0.001169 -1.989 0.0467 *

## ---

## Signif. codes: 0 '***' 0.001 '**' 0.01 '*' 0.05 '.' 0.1 ' ' 1

##

## Residual standard error: 0.4928 on 7264 degrees of freedom

## Multiple R-squared: 0.0005443, Adjusted R-squared: 0.0004067

## F-statistic: 3.956 on 1 and 7264 DF, p-value: 0.04674

The maternal age is significant for multiple biomarkers, and it is tested if there is an association between it and GA and birth type respectively.

S2.4.2 Testing association with GA:

Anova(aov(ALDER_MODER~gestation_uge_f, data=MFR_3742), type="III")

## Anova Table (Type III tests)

##

## Response: ALDER_MODER

## Sum Sq Df F value Pr(>F)

## (Intercept) 416540 1 17055.9857 < 2e-16 ***

## gestation_uge_f 337 5 2.7616 0.01695 *

## Residuals 177303 7260

## ---

## Signif. codes: 0 '***' 0.001 '**' 0.01 '*' 0.05 '.' 0.1 ' ' 1

This is significant, and we have to correct for the maternal age in the analysis

*#grouping*

age <- MFR_3742 %>% mutate(age_group = case_when(ALDER_MODER <20 ~ "<20",

ALDER_MODER>19 & ALDER_MODER<30 ~"20-29",

ALDER_MODER>29 & ALDER_MODER<40~"30-39",

ALDER_MODER>39 ~"40+"))

Figure F

*#Plotting*

age %>% ggplot()+

geom_mosaic(aes(product(age_group), fill=gestation_uge_f))+

labs(x="Age group", y="GA (weeks)", fill="GA (weeks)")+

theme_minimal()


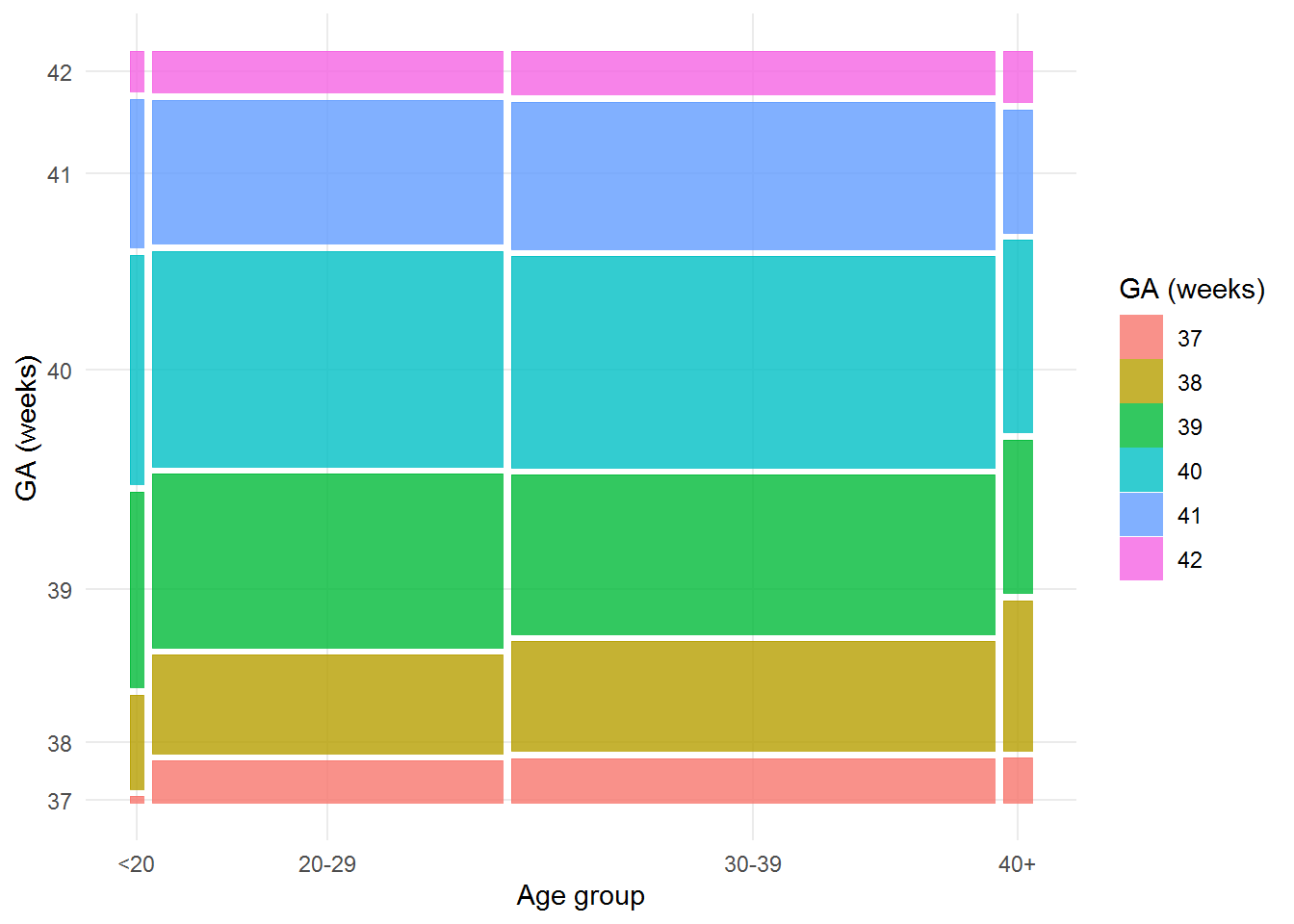


Figure G

age %>% ggplot()+

geom_mosaic(aes(product(gestation_uge_f), fill=age_group))+

labs(x="GA (weeks)", y="Age group", fill="Age group")+

theme_minimal()


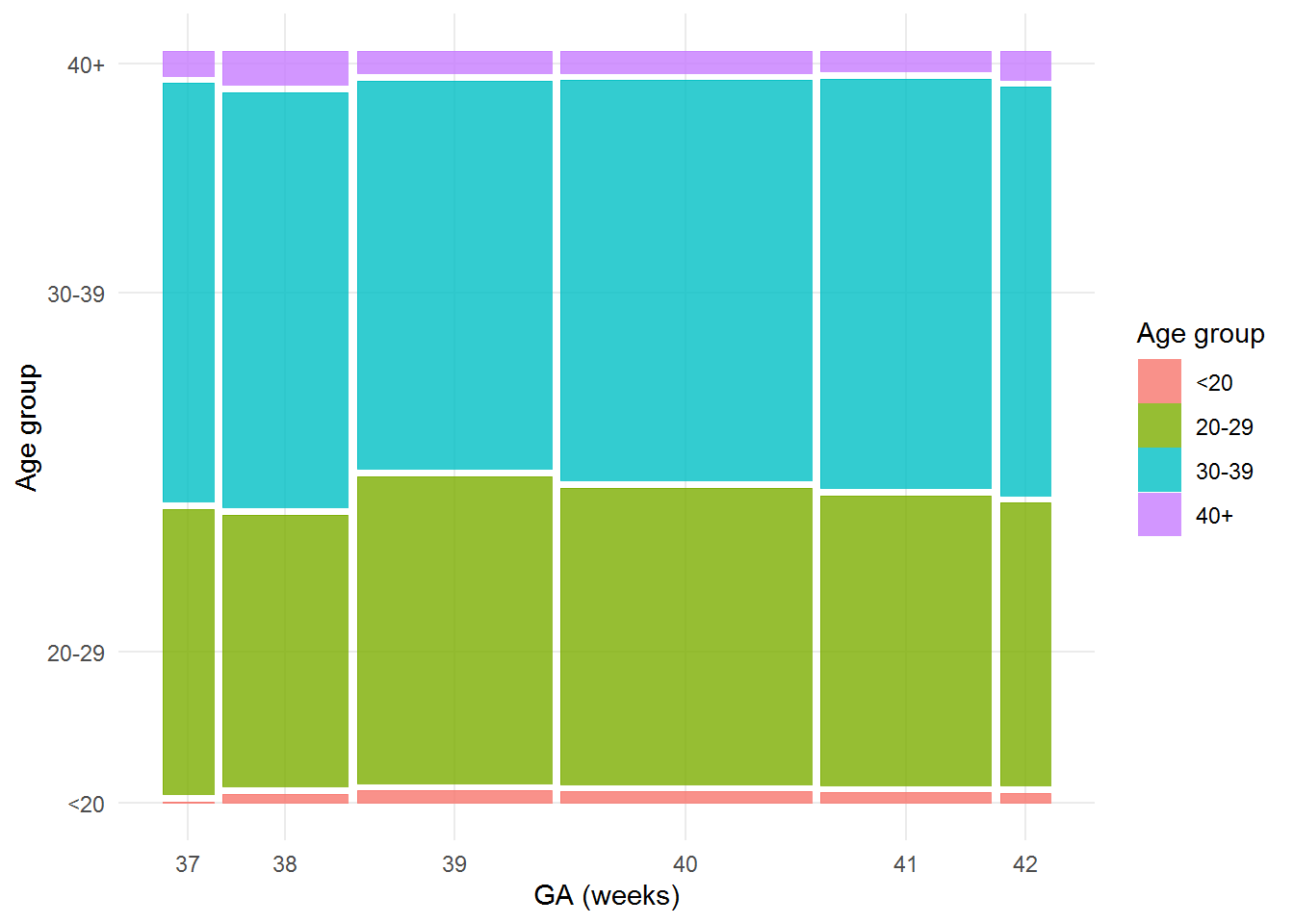


S2.4.3 Testing association with Birth type

Anova(aov(ALDER_MODER~Birth_type, data=MFR_3742), type="III")

## Anova Table (Type III tests)

##

## Response: ALDER_MODER

## Sum Sq Df F value Pr(>F)

## (Intercept) 605021 1 25096.167 < 2.2e-16 ***

## Birth_type 2543 2 52.745 < 2.2e-16 ***

## Residuals 175097 7263

## ---

## Signif. codes: 0 '***' 0.001 '**' 0.01 '*' 0.05 '.' 0.1 ' ' 1

This is significant as well, and it supports the suggestion that we have to correct for the maternal age.

Correction is done in the main analysis.

Figure H

*#Plotting*

age %>% ggplot()+

geom_mosaic(aes(product(age_group), fill=Birth_type))+

labs(x="Age group", y="Birth type", fill="Birth type")+

theme_minimal()


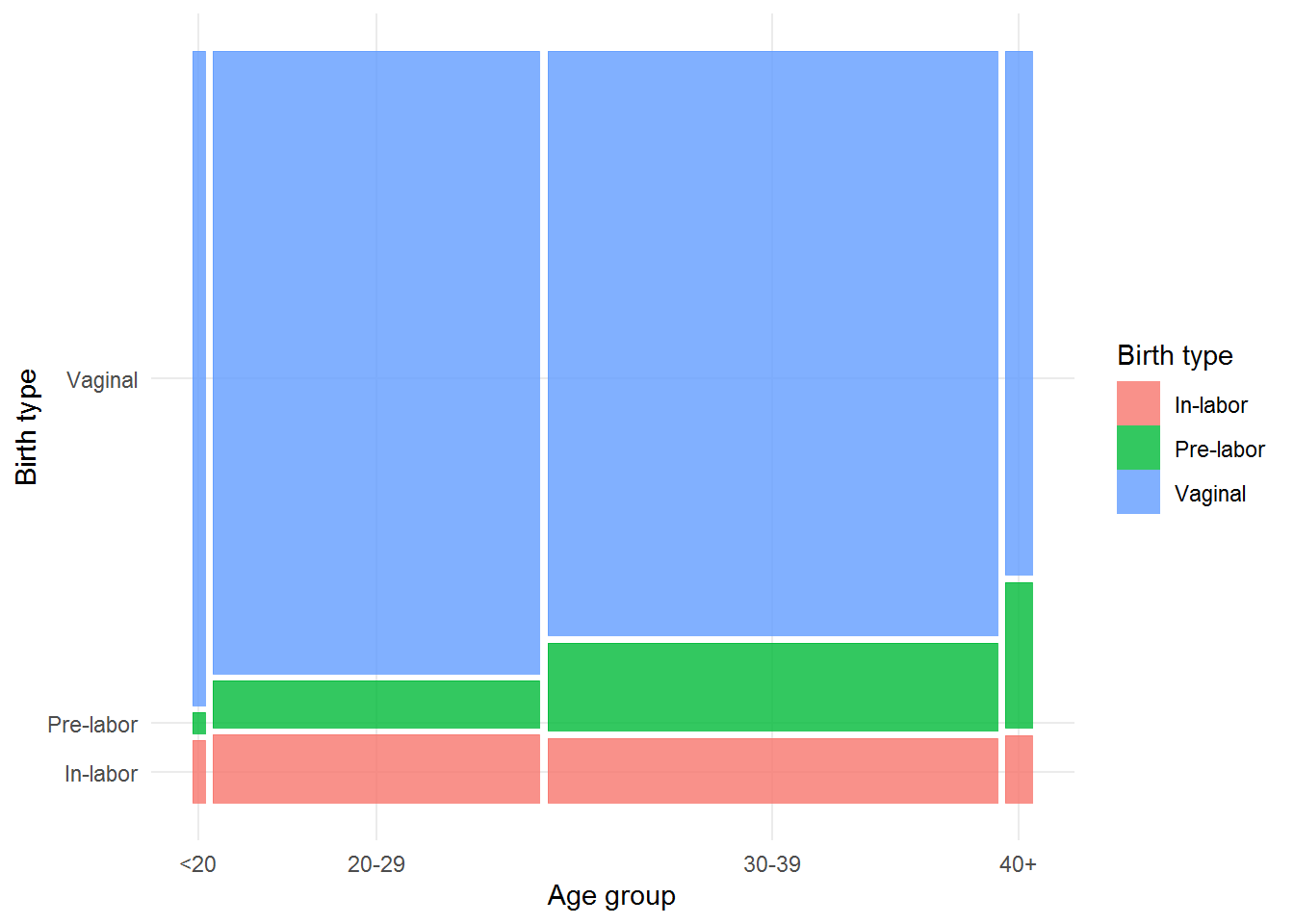


rm(age)

S2.5 Birth weight

The birth weight is expected to correlate much with the gestational age

cor(MFR_3742$gestation_uge, MFR_3742$vaegt, use="complete.obs")

## [1] 0.4280677

This is a very high correlation, and we do not want to correct for this, since it will remove at lot of the effect from GA, and we expect the effect we find to be caused by GA, not the birth weight. Further GA is the explaining variable, some of the variation in the birth weight is caused by GA - not the other way around.

S2.6 Association between type of anaesthetics and type of c-section

Getting anaesthetics information, renaming and removing vaginal birth. If general anaesthetics is registered, it is defined as that, since it is the one we are mostly interested in.

MFR_anaestesi <- as.data.table(rbind(MFR_ANAESTESI_TIL_OPERATION2009, MFR_ANAESTESI_TIL_OPERATION2010, MFR_ANAESTESI_TIL_OPERATION2011))

MFR_anaestesi <- MFR_anaestesi %>%

mutate(anaestesi = ifelse(str_detect(MFR_anaestesi$SKSKODE, "NAAC")==TRUE, "Generel anæstesi",

ifelse(str_detect(MFR_anaestesi$SKSKODE, "NAAD")==TRUE, "Regional anæstesi", "Ingen"))) %>%

select(-c(KODETYPE, SKSKODE))%>% distinct(FK_MFR, anaestesi) %>% arrange(FK_MFR, anaestesi)

MFR_anaestesi <- MFR_anaestesi %>% distinct(FK_MFR, .keep_all = T)

MFR3 <- MFR_anaestesi %>%

right_join(MFR_3742, by="FK_MFR")

MFR3 <- MFR3 %>%

filter(k_type != "Vaginal") %>%

select(FK_MFR, k_type, anaestesi)

table(MFR3$anaestesi, MFR3$k_type, exclude=NULL)

##

## In-labor Pre-labor

## Generel anæstesi 57 21

## Regional anæstesi 250 345

## <NA> 348 348

There is a lot of NA’s, this is likely an error, since no CS are made without anaesthetics. There are two possibilities with these. They could be removed, or they could be changed to regional anesthetics, since it is more likely to forget to note the type of anaesthetics, if it is regional than if it is general.

Testing first possibility:

*# Removing NAs*

MFR_test <- na.omit(MFR3)

chisq.test(table(MFR_test$anaestesi, MFR_test$k_type))

##

## Pearson's Chi-squared test with Yates' continuity correction

##

## data: table(MFR_test$anaestesi, MFR_test$k_type)

## X-squared = 25.58, df = 1, p-value = 4.245e-07

Figure I

MFR_test %>%

mutate(anaestesi=ifelse(anaestesi=="Generel anæstesi", "General anaesthetics", "Regional anaesthetics")) %>%

ggplot()+

geom_mosaic(aes(product(k_type), fill=anaestesi))+

labs(x="Birth type", y="", fill="")+

theme_minimal()


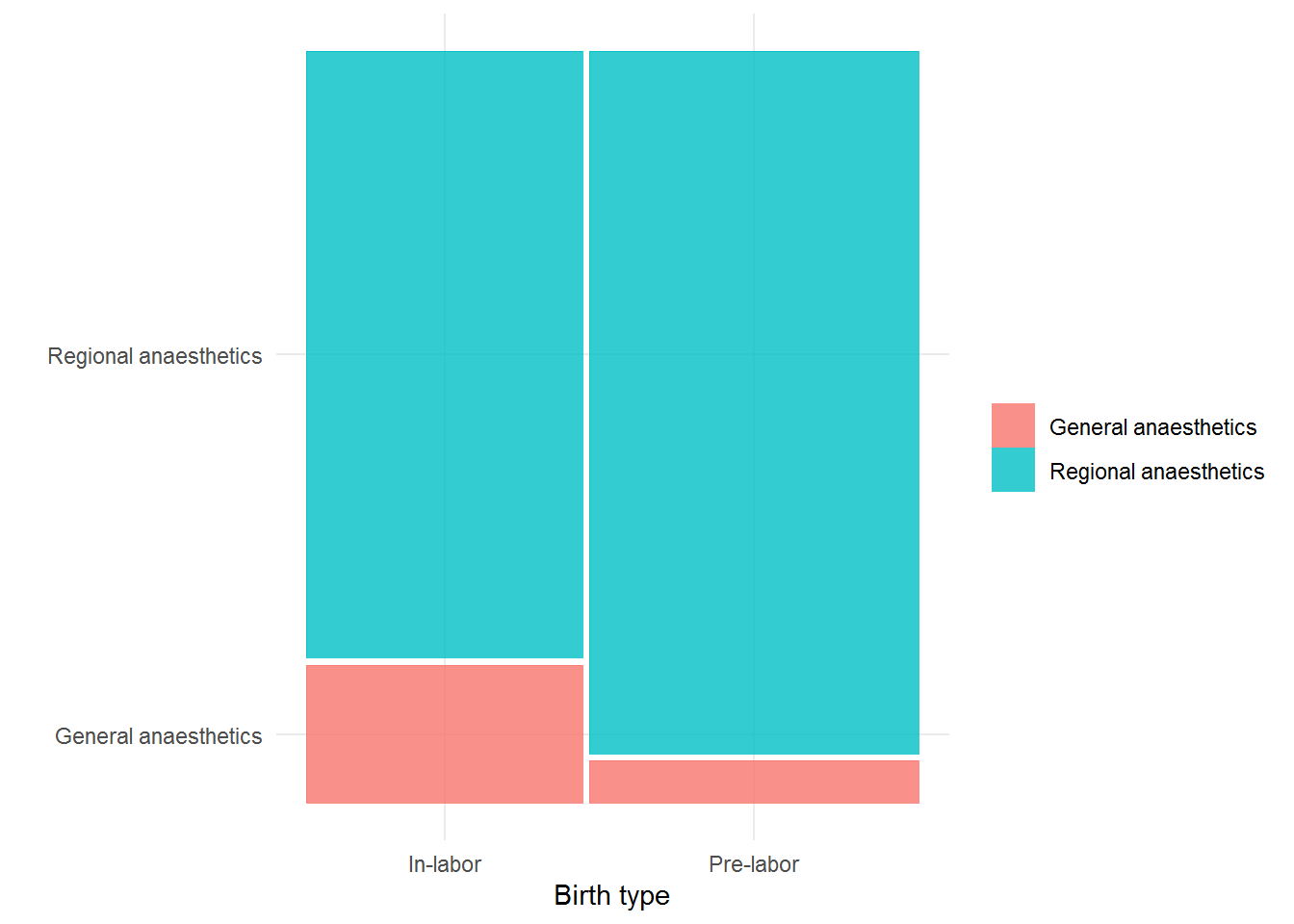


Here it is very clear, that in-labor c-sections have a higher chance of having general anesthesia.

Second possibility:

MFR_test <- MFR3 %>% mutate(anaestesi = ifelse(!is.na(anaestesi), anaestesi, "Regional anæstesi"))

chisq.test(table(MFR_test$anaestesi, MFR_test$k_type))

##

## Pearson's Chi-squared test with Yates' continuity correction

##

## data: table(MFR_test$anaestesi, MFR_test$k_type)

## X-squared = 20.044, df = 1, p-value = 7.568e-06

Figure J

MFR_test %>%

mutate(anaestesi=ifelse(anaestesi=="Generel anæstesi", "General anaesthetics", "Regional anaesthetics")) %>%

ggplot()+

geom_mosaic(aes(product(k_type), fill=anaestesi))+

labs(x="Birth type", y="", fill="")+

theme_minimal()


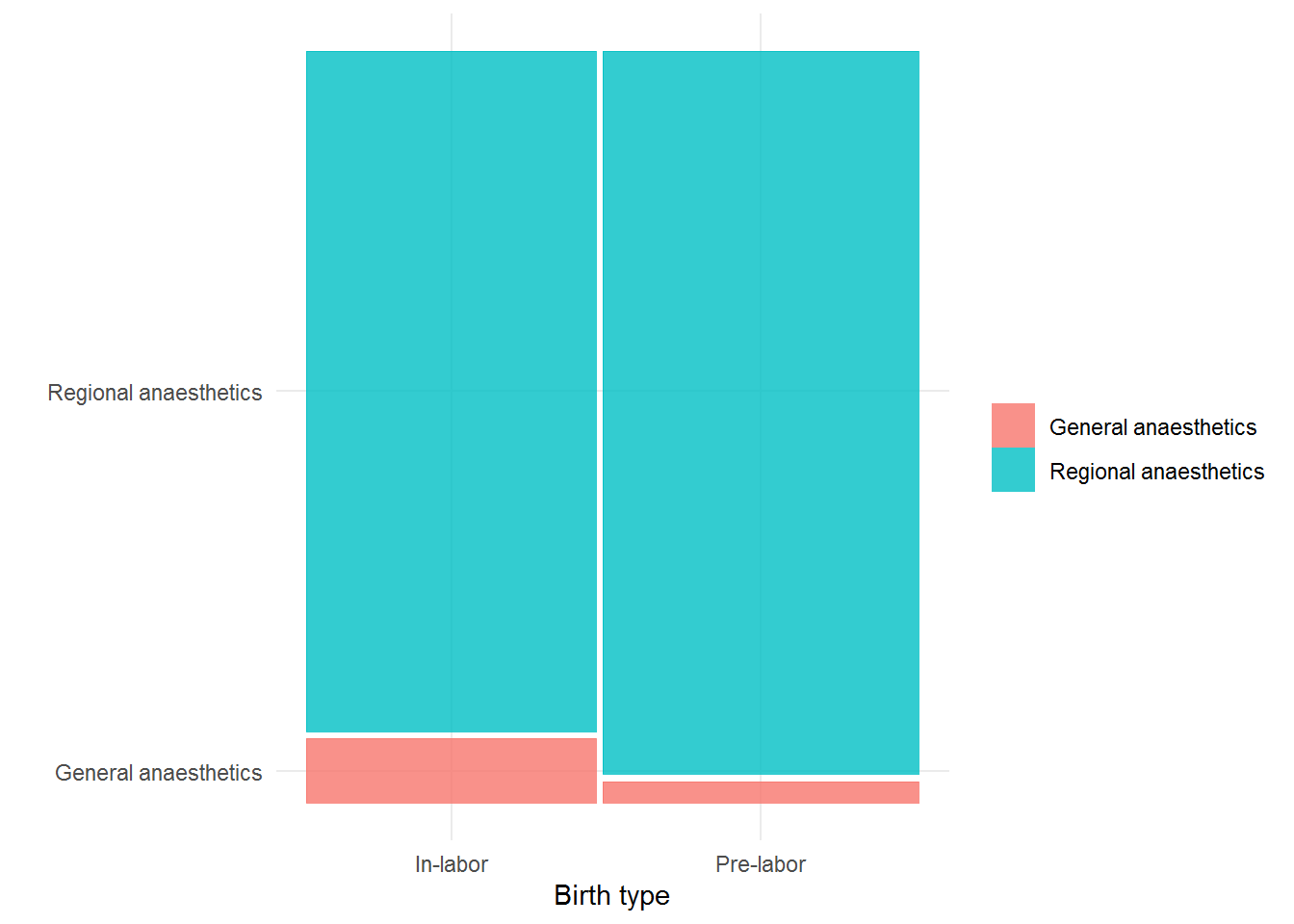


It is still very clear, that there is an association between CS type and type of anaesthetics.
