# Supplementary material for: Delivery Modality Affect Neonatal Levels of Inflammation, Stress, and Growth Factors
Source: Front Pediatr. 2021 Sep 22;9:709765. doi: 10.3389/fped.2021.709765 (PMC8492985; doi:10.3389/fped.2021.709765)
Supplement: Supplementary file 1 [file Data_Sheet_1.zip › S3_Analysis_without_general_anaesthetics.docx]

- S3.1 Loading packages and data
  - S3.1.1 CS is redefines in an understandable way and added to the other dataset
    - Renaming variables with information that are understandable instead of codes
  - S3.1.2 Filtering so we only have the children born on time
  - S3.1.3 Adding anaesthetics variable and removing general anaesthetics
  - S3.1.4 Biomakers is log transformed to make them fit to a normal distribution
  - S3.1.5 Functions are defined
- S3.2 Testing the GA for all biomarkers
- S3.3 Testing birth type
- S3.4 Testing gender

S3 Code for the analysis without general anesthetics

The variables maternal age, BMI_MODER and age at sampling are all explaining some variation for some of the biomarkers, therefore all analysis of variance are made with ANCOVA (analysis of co-variance) controlling for these variables. This is done to make sure the effects seen are because of the variables tested, not one of the other variables (ruling out confounders). Birth weight are not included, since is very correlated with GA, but we expect the effects seen to be because of GA, not weight. Here the analysis is made without general anesthetics to rule out the found effects being from the anesthetics instead of the birthing type.

S3.1 Loading packages and data

S3.1.1 CS is redefines in an understandable way and added to the other dataset

MFR_kejsersnit <- rbind(MFR_KEJSERSNIT2009, MFR_KEJSERSNIT2010, MFR_KEJSERSNIT2011)

MFR_kejsersnit <- MFR_kejsersnit %>%

filter(SKSKODE %**in**% c("KMCA10B", "KMCA10E", "KMCA10A","KMCA10D" )) %>%

mutate(Birth_type = ifelse(SKSKODE == "KMCA10B", "Pre-labor",

ifelse(SKSKODE== "KMCA10E", "In-labor", "Slet"))) %>%

arrange(FK_MFR, Birth_type) %>%

distinct(FK_MFR, .keep_all=TRUE)

MFR2 <- MFR_kejsersnit %>%

right_join(upload_MFR, by="FK_MFR")

Renaming variables with information that are understandable instead of codes

MFR <- MFR2 %>%

mutate(tvilling = ifelse(str_detect(FLERFOLDSGRAVIDITET, "DO300"), "Ja", "Nej")) %>%

mutate(flerfold = ifelse(str_detect(FLERFOLDSGRAVIDITET, "DO30"), "Ja", "Nej")) %>%

mutate(PPROM = ifelse(PPROM=="DO422", "Ja", "Nej")) %>%

mutate(sepsis = ifelse(str_detect(SEPSIS_BARN, "DP36"), "Ja", "Nej")) %>%

replace_na(list(tvilling="Nej")) %>%

replace_na(list(flerfold="Nej")) %>%

replace_na(list(PPROM="Nej")) %>%

replace_na(list(sepsis="Nej")) %>%

replace_na(list(Birth_type="Vaginal")) %>%

mutate(CRP = CRP*1e-06) %>%

mutate(gestation_uge_f = as.factor(gestation_uge)) %>%

filter(gestation_uge > 23 & gestation_uge < 43) %>%

filter(Birth_type != "Slet") %>%

data.table

S3.1.2 Filtering so we only have the children born on time

MFR_3742 <- MFR %>%

filter(gestation_uge > 36 & gestation_uge < 43) %>%

data.table

MFR_3742 <- MFR_3742 %>%

mutate(Gender = ifelse(KOEN_BARN=="M", "Boys", "Girls")) %>%

data.table

rm(MFR_kejsersnit, MFR_KEJSERSNIT2009, MFR_KEJSERSNIT2010, MFR_KEJSERSNIT2011, MFR2, upload_MFR)

S3.1.3 Adding anaesthetics variable and removing general anaesthetics

All individuals with a registration of general anesthetics are removed, also if they have a registration of another anesthetic as well.

MFR_anaestesi <- as.data.table(rbind(MFR_ANAESTESI_TIL_OPERATION2009, MFR_ANAESTESI_TIL_OPERATION2010, MFR_ANAESTESI_TIL_OPERATION2011))

rm(MFR_ANAESTESI_TIL_OPERATION2009, MFR_ANAESTESI_TIL_OPERATION2010, MFR_ANAESTESI_TIL_OPERATION2011)

MFR_anaestesi <- MFR_anaestesi %>%

mutate(anaestesi = ifelse(str_detect(MFR_anaestesi$SKSKODE, "NAAC")==TRUE, "Generel anaestesi",

ifelse(str_detect(MFR_anaestesi$SKSKODE, "NAAD")==TRUE, "Regional anaestesi", "Ingen"))) %>%

select(-c(KODETYPE, SKSKODE)) %>% distinct(FK_MFR, anaestesi) %>% arrange(FK_MFR, anaestesi)

MFR_anaestesi <- MFR_anaestesi %>% distinct(FK_MFR, .keep_all = T)

MFR3 <- MFR_anaestesi %>%

right_join(MFR_3742, by="FK_MFR")

MFR3 %>% summarize(count(anaestesi))

## count(anaestesi).x count(anaestesi).freq

## 1 Generel anaestesi 139

## 2 Regional anaestesi 687

## 3 <NA> 6440

*### So many NAs found, this is probably partly because there is no data for vaginal birth*

*## Removing the individuals with general anesthesia, since there is a difference between what kind of anesthesia is used for*

*# planned and acute cs. Only a few individuals gets general anesthesia.*

MFR1 <- MFR3 %>% filter(anaestesi != "Generel anaestesi" | is.na(anaestesi))

MFR_3742 <- MFR1

rm(MFR1, MFR3)

S3.1.4 Biomakers is log transformed to make them fit to a normal distribution

MFR_3742$logCRP <- log(MFR_3742$CRP)

MFR_3742$logIL18 <- log(MFR_3742$IL18)

MFR_3742$logMCP1 <- log(MFR_3742$MCP1)

MFR_3742$logHSP70 <- log(MFR_3742$HSP70)

MFR_3742$logSTNF_RI <- log(MFR_3742$STNF_RI)

MFR_3742$logEGF <- log(MFR_3742$EGF)

MFR_3742$logBDNF <- log(MFR_3742$BDNF)

MFR_3742$logNT3 <- log(MFR_3742$NT3)

MFR_3742$logS100B <- log(MFR_3742$S100B)

MFR_3742$logVEGF <- log(MFR_3742$VEGF)

MFR_3742$gestation_uge_f <- as.factor(MFR_3742$gestation_uge)

MFR_3740 <- MFR_3742 %>%

mutate(gest = ifelse(gestation_uge == 37, "37",

ifelse(gestation_uge == 38, "38",

ifelse(gestation_uge == 39, "39", "40+"))))

S3.1.5 Functions are defined

*# Testing the effect of the GA on the biomarkers*

bio_gest <- **function**(data, bio){

fit <- aov(log(bio) ~ AGE_AT_COLLECTION + ALDER_MODER + BMI_MODER + gestation_uge_f, data=data)

sf <- Anova(fit, type="III")

sf_lm<- summary.lm(fit)

test <- summary(pairs(emmeans(fit, ~gestation_uge_f)), adjust="holm")

**return**(list(sf, sf_lm$r.squared, test))

}

*#Testing the effect of birth type on the biomarkers*

gest_type <- **function**(data=MFR_3740, bio){

MFR_3740_2 <- data %>%

filter(Birth_type != "Slet") %>%

data.table

m_under <- MFR_3740_2 %>%

filter(Birth_type != "In-labor") %>%

data.table

m_planlagt <- MFR_3740_2 %>%

filter(Birth_type != "Pre-labor") %>%

data.table

m_vaginalt <- MFR_3740_2 %>%

filter(Birth_type != "Vaginal") %>%

data.table

vag_in <- Anova(aov(m_planlagt[[bio]] ~ AGE_AT_COLLECTION + ALDER_MODER + BMI_MODER + gest + Birth_type, data=m_planlagt), type="III")

vag_pre <- Anova(aov(m_under[[bio]] ~ AGE_AT_COLLECTION + ALDER_MODER + BMI_MODER + gest + Birth_type, data=m_under), type="III")

in_pre <- Anova(aov(m_vaginalt[[bio]] ~ AGE_AT_COLLECTION + ALDER_MODER + BMI_MODER + gest + Birth_type, data=m_vaginalt), type="III")

fit <- aov(MFR_3740_2[[bio]] ~ AGE_AT_COLLECTION + ALDER_MODER + BMI_MODER + Birth_type + gest + Birth_type:gest, data=MFR_3740_2)

test <- summary(pairs(emmeans(fit, ~ Birth_type|gest)),by=NULL, adjust="holm")

**return**(list("Vaginal-inlabor"=vag_in, "Vaginal-prelabor"=vag_pre,"Inlabor-prelabor"=in_pre,"Pairwise test"=test))

}

*#Testing the effect of gender on the biomarkers*

koen_type <- **function**(data=MFR_3740, bio){

MFR_3740_2 <- data %>%

filter(Birth_type != "Slet") %>%

data.table

boys <- MFR_3740_2 %>%

filter(Gender == "Boys") %>%

data.table

girls <- MFR_3740_2 %>%

filter(Gender == "Girls") %>%

data.table

b<-Anova(aov(boys[[bio]] ~ AGE_AT_COLLECTION + ALDER_MODER + BMI_MODER + Birth_type, data=boys), type="III")

g<-Anova(aov(girls[[bio]] ~ AGE_AT_COLLECTION + ALDER_MODER + BMI_MODER + Birth_type, data=girls), type="III")

fit <- aov(MFR_3740_2[[bio]] ~ AGE_AT_COLLECTION + ALDER_MODER + BMI_MODER + Birth_type + KOEN_BARN + Birth_type:KOEN_BARN, data=MFR_3740_2)

test <- summary(pairs(emmeans(fit, ~ KOEN_BARN|Birth_type)), by=NULL, adjust="holm")

**return**(list("Boys"=b, "Girls"=g,"Pairwise test"=test))

}

S3.2 Testing the GA for all biomarkers

Figure 3 in the paper.

bio_gest(data=MFR_3742, bio=MFR_3742$CRP)

## [[1]]

## Anova Table (Type III tests)

##

## Response: log(bio)

## Sum Sq Df F value Pr(>F)

## (Intercept) 84.9 1 154.8944 < 2.2e-16 ***

## AGE_AT_COLLECTION 10.9 1 19.8763 8.394e-06 ***

## ALDER_MODER 9.7 1 17.7624 2.535e-05 ***

## BMI_MODER 0.2 1 0.3342 0.5632

## gestation_uge_f 210.2 5 76.7359 < 2.2e-16 ***

## Residuals 3746.5 6839

## ---

## Signif. codes: 0 '***' 0.001 '**' 0.01 '*' 0.05 '.' 0.1 ' ' 1

##

## [[2]]

## [1] 0.05833902

##

## [[3]]

## contrast estimate SE df t.ratio p.value

## 37 - 38 -0.1051 0.0435 6839 -2.419 0.0509

## 37 - 39 -0.2759 0.0412 6839 -6.691 <.0001

## 37 - 40 -0.4773 0.0402 6839 -11.861 <.0001

## 37 - 41 -0.5139 0.0418 6839 -12.304 <.0001

## 37 - 42 -0.5771 0.0518 6839 -11.141 <.0001

## 38 - 39 -0.1708 0.0298 6839 -5.740 <.0001

## 38 - 40 -0.3722 0.0284 6839 -13.105 <.0001

## 38 - 41 -0.4088 0.0305 6839 -13.385 <.0001

## 38 - 42 -0.4720 0.0433 6839 -10.902 <.0001

## 39 - 40 -0.2014 0.0248 6839 -8.117 <.0001

## 39 - 41 -0.2380 0.0272 6839 -8.737 <.0001

## 39 - 42 -0.3012 0.0411 6839 -7.336 <.0001

## 40 - 41 -0.0366 0.0257 6839 -1.423 0.2572

## 40 - 42 -0.0998 0.0401 6839 -2.492 0.0509

## 41 - 42 -0.0632 0.0416 6839 -1.520 0.2572

##

## Results are given on the log (not the response) scale.

## P value adjustment: holm method for 15 tests

bio_gest(data=MFR_3742, bio=MFR_3742$MCP1)

## [[1]]

## Anova Table (Type III tests)

##

## Response: log(bio)

## Sum Sq Df F value Pr(>F)

## (Intercept) 2337.27 1 12668.0620 < 2e-16 ***

## AGE_AT_COLLECTION 46.16 1 250.1782 < 2e-16 ***

## ALDER_MODER 0.94 1 5.0745 0.02431 *

## BMI_MODER 0.17 1 0.9246 0.33632

## gestation_uge_f 25.11 5 27.2207 < 2e-16 ***

## Residuals 1261.80 6839

## ---

## Signif. codes: 0 '***' 0.001 '**' 0.01 '*' 0.05 '.' 0.1 ' ' 1

##

## [[2]]

## [1] 0.05335318

##

## [[3]]

## contrast estimate SE df t.ratio p.value

## 37 - 38 0.0104 0.0252 6839 0.413 0.9482

## 37 - 39 -0.0530 0.0239 6839 -2.214 0.1344

## 37 - 40 -0.1296 0.0234 6839 -5.550 <.0001

## 37 - 41 -0.1424 0.0242 6839 -5.875 <.0001

## 37 - 42 -0.1666 0.0301 6839 -5.542 <.0001

## 38 - 39 -0.0634 0.0173 6839 -3.671 0.0015

## 38 - 40 -0.1400 0.0165 6839 -8.497 <.0001

## 38 - 41 -0.1528 0.0177 6839 -8.622 <.0001

## 38 - 42 -0.1770 0.0251 6839 -7.045 <.0001

## 39 - 40 -0.0766 0.0144 6839 -5.323 <.0001

## 39 - 41 -0.0894 0.0158 6839 -5.657 <.0001

## 39 - 42 -0.1136 0.0238 6839 -4.769 <.0001

## 40 - 41 -0.0128 0.0149 6839 -0.856 0.9482

## 40 - 42 -0.0370 0.0232 6839 -1.591 0.4466

## 41 - 42 -0.0242 0.0241 6839 -1.003 0.9482

##

## Results are given on the log (not the response) scale.

## P value adjustment: holm method for 15 tests

bio_gest(data=MFR_3742, bio=MFR_3742$IL18)

## [[1]]

## Anova Table (Type III tests)

##

## Response: log(bio)

## Sum Sq Df F value Pr(>F)

## (Intercept) 690.19 1 3838.7856 < 2.2e-16 ***

## AGE_AT_COLLECTION 0.01 1 0.0361 0.8494

## ALDER_MODER 0.01 1 0.0648 0.7991

## BMI_MODER 0.39 1 2.1817 0.1397

## gestation_uge_f 6.73 5 7.4845 5.146e-07 ***

## Residuals 1229.60 6839

## ---

## Signif. codes: 0 '***' 0.001 '**' 0.01 '*' 0.05 '.' 0.1 ' ' 1

##

## [[2]]

## [1] 0.005890378

##

## [[3]]

## contrast estimate SE df t.ratio p.value

## 37 - 38 -0.00907 0.0249 6839 -0.364 1.0000

## 37 - 39 -0.01723 0.0236 6839 -0.729 1.0000

## 37 - 40 -0.05753 0.0231 6839 -2.495 0.0756

## 37 - 41 -0.06460 0.0239 6839 -2.700 0.0487

## 37 - 42 -0.12390 0.0297 6839 -4.175 0.0004

## 38 - 39 -0.00816 0.0170 6839 -0.479 1.0000

## 38 - 40 -0.04846 0.0163 6839 -2.978 0.0291

## 38 - 41 -0.05553 0.0175 6839 -3.174 0.0181

## 38 - 42 -0.11483 0.0248 6839 -4.629 0.0001

## 39 - 40 -0.04030 0.0142 6839 -2.835 0.0367

## 39 - 41 -0.04737 0.0156 6839 -3.036 0.0265

## 39 - 42 -0.10666 0.0235 6839 -4.535 0.0001

## 40 - 41 -0.00707 0.0147 6839 -0.480 1.0000

## 40 - 42 -0.06637 0.0229 6839 -2.892 0.0345

## 41 - 42 -0.05929 0.0238 6839 -2.489 0.0756

##

## Results are given on the log (not the response) scale.

## P value adjustment: holm method for 15 tests

bio_gest(data=MFR_3742, bio=MFR_3742$HSP70)

## [[1]]

## Anova Table (Type III tests)

##

## Response: log(bio)

## Sum Sq Df F value Pr(>F)

## (Intercept) 10210.4 1 1.0987e+05 < 2e-16 ***

## AGE_AT_COLLECTION 0.0 1 3.6930e-01 0.54340

## ALDER_MODER 0.1 1 1.5292e+00 0.21628

## BMI_MODER 0.5 1 5.6669e+00 0.01732 *

## gestation_uge_f 1.0 5 2.1793e+00 0.05360 .

## Residuals 635.6 6839

## ---

## Signif. codes: 0 '***' 0.001 '**' 0.01 '*' 0.05 '.' 0.1 ' ' 1

##

## [[2]]

## [1] 0.002686715

##

## [[3]]

## contrast estimate SE df t.ratio p.value

## 37 - 38 0.00052 0.0179 6839 0.029 1.0000

## 37 - 39 0.02790 0.0170 6839 1.643 1.0000

## 37 - 40 0.03187 0.0166 6839 1.922 0.7097

## 37 - 41 0.01958 0.0172 6839 1.138 1.0000

## 37 - 42 0.00603 0.0213 6839 0.283 1.0000

## 38 - 39 0.02738 0.0123 6839 2.234 0.3571

## 38 - 40 0.03135 0.0117 6839 2.680 0.1108

## 38 - 41 0.01906 0.0126 6839 1.515 1.0000

## 38 - 42 0.00551 0.0178 6839 0.309 1.0000

## 39 - 40 0.00396 0.0102 6839 0.388 1.0000

## 39 - 41 -0.00832 0.0112 6839 -0.742 1.0000

## 39 - 42 -0.02187 0.0169 6839 -1.294 1.0000

## 40 - 41 -0.01229 0.0106 6839 -1.159 1.0000

## 40 - 42 -0.02584 0.0165 6839 -1.566 1.0000

## 41 - 42 -0.01355 0.0171 6839 -0.791 1.0000

##

## Results are given on the log (not the response) scale.

## P value adjustment: holm method for 15 tests

bio_gest(data=MFR_3742, bio=MFR_3742$STNF_RI)

## [[1]]

## Anova Table (Type III tests)

##

## Response: log(bio)

## Sum Sq Df F value Pr(>F)

## (Intercept) 1726.56 1 6040.7580 < 2.2e-16 ***

## AGE_AT_COLLECTION 41.07 1 143.7074 < 2.2e-16 ***

## ALDER_MODER 2.34 1 8.1937 0.004216 **

## BMI_MODER 2.59 1 9.0547 0.002630 **

## gestation_uge_f 3.64 5 2.5502 0.025922 *

## Residuals 1954.71 6839

## ---

## Signif. codes: 0 '***' 0.001 '**' 0.01 '*' 0.05 '.' 0.1 ' ' 1

##

## [[2]]

## [1] 0.02466056

##

## [[3]]

## contrast estimate SE df t.ratio p.value

## 37 - 38 -0.01005 0.0314 6839 -0.320 1.0000

## 37 - 39 0.04772 0.0298 6839 1.602 0.9824

## 37 - 40 0.03254 0.0291 6839 1.119 1.0000

## 37 - 41 0.05276 0.0302 6839 1.749 0.8738

## 37 - 42 0.06564 0.0374 6839 1.754 0.8738

## 38 - 39 0.05777 0.0215 6839 2.688 0.1010

## 38 - 40 0.04258 0.0205 6839 2.076 0.4554

## 38 - 41 0.06281 0.0221 6839 2.847 0.0664

## 38 - 42 0.07568 0.0313 6839 2.420 0.2021

## 39 - 40 -0.01519 0.0179 6839 -0.847 1.0000

## 39 - 41 0.00504 0.0197 6839 0.256 1.0000

## 39 - 42 0.01791 0.0297 6839 0.604 1.0000

## 40 - 41 0.02023 0.0186 6839 1.088 1.0000

## 40 - 42 0.03310 0.0289 6839 1.144 1.0000

## 41 - 42 0.01287 0.0300 6839 0.429 1.0000

##

## Results are given on the log (not the response) scale.

## P value adjustment: holm method for 15 tests

bio_gest(data=MFR_3742, bio=MFR_3742$EGF)

## [[1]]

## Anova Table (Type III tests)

##

## Response: log(bio)

## Sum Sq Df F value Pr(>F)

## (Intercept) 662.90 1 3120.9713 < 2.2e-16 ***

## AGE_AT_COLLECTION 2.58 1 12.1311 0.000499 ***

## ALDER_MODER 0.28 1 1.3367 0.247660

## BMI_MODER 0.48 1 2.2534 0.133369

## gestation_uge_f 3.33 5 3.1392 0.007815 **

## Residuals 1452.61 6839

## ---

## Signif. codes: 0 '***' 0.001 '**' 0.01 '*' 0.05 '.' 0.1 ' ' 1

##

## [[2]]

## [1] 0.00462401

##

## [[3]]

## contrast estimate SE df t.ratio p.value

## 37 - 38 0.0122 0.0271 6839 0.449 1.0000

## 37 - 39 0.0398 0.0257 6839 1.550 0.9691

## 37 - 40 0.0278 0.0251 6839 1.108 1.0000

## 37 - 41 0.0654 0.0260 6839 2.515 0.1431

## 37 - 42 0.0823 0.0323 6839 2.553 0.1391

## 38 - 39 0.0277 0.0185 6839 1.492 0.9691

## 38 - 40 0.0156 0.0177 6839 0.882 1.0000

## 38 - 41 0.0533 0.0190 6839 2.800 0.0768

## 38 - 42 0.0702 0.0270 6839 2.603 0.1295

## 39 - 40 -0.0120 0.0154 6839 -0.780 1.0000

## 39 - 41 0.0256 0.0170 6839 1.510 0.9691

## 39 - 42 0.0425 0.0256 6839 1.664 0.8655

## 40 - 41 0.0377 0.0160 6839 2.350 0.2071

## 40 - 42 0.0546 0.0249 6839 2.189 0.2866

## 41 - 42 0.0169 0.0259 6839 0.654 1.0000

##

## Results are given on the log (not the response) scale.

## P value adjustment: holm method for 15 tests

bio_gest(data=MFR_3742, bio=MFR_3742$VEGF)

## [[1]]

## Anova Table (Type III tests)

##

## Response: log(bio)

## Sum Sq Df F value Pr(>F)

## (Intercept) 1143.88 1 5881.0994 < 2.2e-16 ***

## AGE_AT_COLLECTION 48.91 1 251.4839 < 2.2e-16 ***

## ALDER_MODER 0.13 1 0.6434 0.422493

## BMI_MODER 0.41 1 2.1038 0.146980

## gestation_uge_f 3.16 5 3.2532 0.006165 **

## Residuals 1330.19 6839

## ---

## Signif. codes: 0 '***' 0.001 '**' 0.01 '*' 0.05 '.' 0.1 ' ' 1

##

## [[2]]

## [1] 0.03832058

##

## [[3]]

## contrast estimate SE df t.ratio p.value

## 37 - 38 0.002719 0.0259 6839 0.105 1.0000

## 37 - 39 0.035446 0.0246 6839 1.443 0.8952

## 37 - 40 0.036815 0.0240 6839 1.535 0.8734

## 37 - 41 0.064878 0.0249 6839 2.607 0.1283

## 37 - 42 0.065375 0.0309 6839 2.118 0.4105

## 38 - 39 0.032726 0.0177 6839 1.846 0.6499

## 38 - 40 0.034096 0.0169 6839 2.015 0.4837

## 38 - 41 0.062159 0.0182 6839 3.415 0.0096

## 38 - 42 0.062655 0.0258 6839 2.429 0.1974

## 39 - 40 0.001369 0.0148 6839 0.093 1.0000

## 39 - 41 0.029433 0.0162 6839 1.813 0.6499

## 39 - 42 0.029929 0.0245 6839 1.223 1.0000

## 40 - 41 0.028063 0.0153 6839 1.830 0.6499

## 40 - 42 0.028559 0.0239 6839 1.197 1.0000

## 41 - 42 0.000496 0.0248 6839 0.020 1.0000

##

## Results are given on the log (not the response) scale.

## P value adjustment: holm method for 15 tests

bio_gest(data=MFR_3742, bio=MFR_3742$S100B)

## [[1]]

## Anova Table (Type III tests)

##

## Response: log(bio)

## Sum Sq Df F value Pr(>F)

## (Intercept) 2233.99 1 7291.6730 < 2.2e-16 ***

## AGE_AT_COLLECTION 0.45 1 1.4669 0.225877

## ALDER_MODER 0.00 1 0.0157 0.900152

## BMI_MODER 0.20 1 0.6511 0.419734

## gestation_uge_f 4.85 5 3.1669 0.007378 **

## Residuals 2095.30 6839

## ---

## Signif. codes: 0 '***' 0.001 '**' 0.01 '*' 0.05 '.' 0.1 ' ' 1

##

## [[2]]

## [1] 0.002572578

##

## [[3]]

## contrast estimate SE df t.ratio p.value

## 37 - 38 0.00495 0.0325 6839 0.152 1.0000

## 37 - 39 0.03298 0.0308 6839 1.069 1.0000

## 37 - 40 0.04480 0.0301 6839 1.489 0.9563

## 37 - 41 0.07865 0.0312 6839 2.518 0.1656

## 37 - 42 0.08429 0.0387 6839 2.176 0.3256

## 38 - 39 0.02802 0.0223 6839 1.259 1.0000

## 38 - 40 0.03985 0.0212 6839 1.876 0.6066

## 38 - 41 0.07370 0.0228 6839 3.227 0.0189

## 38 - 42 0.07934 0.0324 6839 2.450 0.1859

## 39 - 40 0.01183 0.0186 6839 0.638 1.0000

## 39 - 41 0.04568 0.0204 6839 2.242 0.2998

## 39 - 42 0.05131 0.0307 6839 1.671 0.7577

## 40 - 41 0.03385 0.0192 6839 1.758 0.7086

## 40 - 42 0.03949 0.0300 6839 1.318 1.0000

## 41 - 42 0.00564 0.0311 6839 0.181 1.0000

##

## Results are given on the log (not the response) scale.

## P value adjustment: holm method for 15 tests

bio_gest(data=MFR_3742, bio=MFR_3742$BDNF)

## [[1]]

## Anova Table (Type III tests)

##

## Response: log(bio)

## Sum Sq Df F value Pr(>F)

## (Intercept) 2559.7 1 4635.7039 < 2.2e-16 ***

## AGE_AT_COLLECTION 4.8 1 8.7315 0.003138 **

## ALDER_MODER 3.7 1 6.7738 0.009271 **

## BMI_MODER 4.8 1 8.6150 0.003345 **

## gestation_uge_f 5.1 5 1.8363 0.102192

## Residuals 3776.3 6839

## ---

## Signif. codes: 0 '***' 0.001 '**' 0.01 '*' 0.05 '.' 0.1 ' ' 1

##

## [[2]]

## [1] 0.004993677

##

## [[3]]

## contrast estimate SE df t.ratio p.value

## 37 - 38 -0.06242 0.0436 6839 -1.430 1.0000

## 37 - 39 -0.03710 0.0414 6839 -0.896 1.0000

## 37 - 40 -0.07166 0.0404 6839 -1.774 0.9903

## 37 - 41 -0.03944 0.0419 6839 -0.941 1.0000

## 37 - 42 0.03072 0.0520 6839 0.591 1.0000

## 38 - 39 0.02532 0.0299 6839 0.848 1.0000

## 38 - 40 -0.00924 0.0285 6839 -0.324 1.0000

## 38 - 41 0.02298 0.0307 6839 0.749 1.0000

## 38 - 42 0.09315 0.0435 6839 2.143 0.4503

## 39 - 40 -0.03456 0.0249 6839 -1.387 1.0000

## 39 - 41 -0.00234 0.0273 6839 -0.086 1.0000

## 39 - 42 0.06782 0.0412 6839 1.645 1.0000

## 40 - 41 0.03222 0.0258 6839 1.247 1.0000

## 40 - 42 0.10238 0.0402 6839 2.546 0.1638

## 41 - 42 0.07016 0.0417 6839 1.681 1.0000

##

## Results are given on the log (not the response) scale.

## P value adjustment: holm method for 15 tests

bio_gest(data=MFR_3742, bio=MFR_3742$NT3)

## [[1]]

## Anova Table (Type III tests)

##

## Response: log(bio)

## Sum Sq Df F value Pr(>F)

## (Intercept) 138.00 1 570.0325 < 2.2e-16 ***

## AGE_AT_COLLECTION 4.28 1 17.6913 2.631e-05 ***

## ALDER_MODER 0.82 1 3.3827 0.06593 .

## BMI_MODER 0.56 1 2.3014 0.12930

## gestation_uge_f 1.16 5 0.9595 0.44117

## Residuals 1655.65 6839

## ---

## Signif. codes: 0 '***' 0.001 '**' 0.01 '*' 0.05 '.' 0.1 ' ' 1

##

## [[2]]

## [1] 0.004112314

##

## [[3]]

## contrast estimate SE df t.ratio p.value

## 37 - 38 -0.04018 0.0289 6839 -1.390 1.0000

## 37 - 39 -0.01898 0.0274 6839 -0.692 1.0000

## 37 - 40 -0.04543 0.0268 6839 -1.698 1.0000

## 37 - 41 -0.02898 0.0278 6839 -1.044 1.0000

## 37 - 42 -0.02297 0.0344 6839 -0.667 1.0000

## 38 - 39 0.02120 0.0198 6839 1.072 1.0000

## 38 - 40 -0.00525 0.0189 6839 -0.278 1.0000

## 38 - 41 0.01120 0.0203 6839 0.552 1.0000

## 38 - 42 0.01721 0.0288 6839 0.598 1.0000

## 39 - 40 -0.02645 0.0165 6839 -1.604 1.0000

## 39 - 41 -0.01000 0.0181 6839 -0.552 1.0000

## 39 - 42 -0.00399 0.0273 6839 -0.146 1.0000

## 40 - 41 0.01645 0.0171 6839 0.962 1.0000

## 40 - 42 0.02246 0.0266 6839 0.844 1.0000

## 41 - 42 0.00601 0.0276 6839 0.217 1.0000

##

## Results are given on the log (not the response) scale.

## P value adjustment: holm method for 15 tests

S3.3 Testing birth type

Figure 1 i paper

cleaning up the data and splitting it up

Testing Here an ancova is made for each biomaker for each of the pairs of birthtypes. This is further corrected for GA

gest_type(bio="logCRP")

## $`Vaginal-inlabor`

## Anova Table (Type III tests)

##

## Response: m_planlagt[[bio]]

## Sum Sq Df F value Pr(>F)

## (Intercept) 75.4 1 142.1598 < 2.2e-16 ***

## AGE_AT_COLLECTION 16.4 1 30.9219 2.799e-08 ***

## ALDER_MODER 5.5 1 10.3646 0.0012912 **

## BMI_MODER 0.9 1 1.7139 0.1905241

## gest 108.9 3 68.4344 < 2.2e-16 ***

## Birth_type 7.6 1 14.3886 0.0001501 ***

## Residuals 3275.2 6172

## ---

## Signif. codes: 0 '***' 0.001 '**' 0.01 '*' 0.05 '.' 0.1 ' ' 1

##

## $`Vaginal-prelabor`

## Anova Table (Type III tests)

##

## Response: m_under[[bio]]

## Sum Sq Df F value Pr(>F)

## (Intercept) 110.6 1 205.6231 < 2.2e-16 ***

## AGE_AT_COLLECTION 17.7 1 32.9575 9.861e-09 ***

## ALDER_MODER 4.7 1 8.7415 0.003122 **

## BMI_MODER 0.8 1 1.4118 0.234801

## gest 98.0 3 60.7341 < 2.2e-16 ***

## Birth_type 64.5 1 119.7892 < 2.2e-16 ***

## Residuals 3367.9 6259

## ---

## Signif. codes: 0 '***' 0.001 '**' 0.01 '*' 0.05 '.' 0.1 ' ' 1

##

## $`Inlabor-prelabor`

## Anova Table (Type III tests)

##

## Response: m_vaginalt[[bio]]

## Sum Sq Df F value Pr(>F)

## (Intercept) 31.17 1 53.8752 3.852e-13 ***

## AGE_AT_COLLECTION 0.23 1 0.3972 0.5286737

## ALDER_MODER 0.01 1 0.0117 0.9137508

## BMI_MODER 0.99 1 1.7054 0.1918235

## gest 24.03 3 13.8457 6.956e-09 ***

## Birth_type 8.54 1 14.7560 0.0001285 ***

## Residuals 718.00 1241

## ---

## Signif. codes: 0 '***' 0.001 '**' 0.01 '*' 0.05 '.' 0.1 ' ' 1

##

## $`Pairwise test`

## contrast gest estimate SE df t.ratio p.value

## (In-labor) - (Pre-labor) 37 0.0239 0.1427 6833 0.167 0.8670

## (In-labor) - Vaginal 37 -0.2959 0.1285 6833 -2.302 0.1068

## (Pre-labor) - Vaginal 37 -0.3198 0.0883 6833 -3.622 0.0029

## (In-labor) - (Pre-labor) 38 0.2304 0.0915 6833 2.518 0.0829

## (In-labor) - Vaginal 38 -0.1477 0.0856 6833 -1.725 0.2535

## (Pre-labor) - Vaginal 38 -0.3781 0.0523 6833 -7.232 <.0001

## (In-labor) - (Pre-labor) 39 0.2493 0.0843 6833 2.956 0.0250

## (In-labor) - Vaginal 39 -0.1050 0.0731 6833 -1.436 0.3019

## (Pre-labor) - Vaginal 39 -0.3543 0.0515 6833 -6.880 <.0001

## (In-labor) - (Pre-labor) 40+ 0.2615 0.1227 6833 2.131 0.1323

## (In-labor) - Vaginal 40+ -0.1019 0.0411 6833 -2.477 0.0829

## (Pre-labor) - Vaginal 40+ -0.3634 0.1169 6833 -3.109 0.0170

##

## P value adjustment: holm method for 12 tests

gest_type(bio="logMCP1")

## $`Vaginal-inlabor`

## Anova Table (Type III tests)

##

## Response: m_planlagt[[bio]]

## Sum Sq Df F value Pr(>F)

## (Intercept) 1888.18 1 10196.8533 < 2.2e-16 ***

## AGE_AT_COLLECTION 50.95 1 275.1376 < 2.2e-16 ***

## ALDER_MODER 0.45 1 2.4242 0.1195

## BMI_MODER 0.02 1 0.0818 0.7749

## gest 10.12 3 18.2097 9.251e-12 ***

## Birth_type 0.08 1 0.4278 0.5131

## Residuals 1142.89 6172

## ---

## Signif. codes: 0 '***' 0.001 '**' 0.01 '*' 0.05 '.' 0.1 ' ' 1

##

## $`Vaginal-prelabor`

## Anova Table (Type III tests)

##

## Response: m_under[[bio]]

## Sum Sq Df F value Pr(>F)

## (Intercept) 1863.05 1 10207.4963 < 2.2e-16 ***

## AGE_AT_COLLECTION 49.13 1 269.1927 < 2.2e-16 ***

## ALDER_MODER 0.37 1 2.0441 0.1528

## BMI_MODER 0.06 1 0.3102 0.5776

## gest 10.43 3 19.0544 2.689e-12 ***

## Birth_type 13.71 1 75.0965 < 2.2e-16 ***

## Residuals 1142.38 6259

## ---

## Signif. codes: 0 '***' 0.001 '**' 0.01 '*' 0.05 '.' 0.1 ' ' 1

##

## $`Inlabor-prelabor`

## Anova Table (Type III tests)

##

## Response: m_vaginalt[[bio]]

## Sum Sq Df F value Pr(>F)

## (Intercept) 342.11 1 2032.1128 < 2.2e-16 ***

## AGE_AT_COLLECTION 3.39 1 20.1163 7.962e-06 ***

## ALDER_MODER 0.24 1 1.4304 0.23193

## BMI_MODER 0.02 1 0.0904 0.76377

## gest 1.20 3 2.3730 0.06873 .

## Birth_type 4.77 1 28.3513 1.200e-07 ***

## Residuals 208.92 1241

## ---

## Signif. codes: 0 '***' 0.001 '**' 0.01 '*' 0.05 '.' 0.1 ' ' 1

##

## $`Pairwise test`

## contrast gest estimate SE df t.ratio p.value

## (In-labor) - (Pre-labor) 37 0.1033 0.0831 6833 1.243 1.0000

## (In-labor) - Vaginal 37 -0.0416 0.0748 6833 -0.556 1.0000

## (Pre-labor) - Vaginal 37 -0.1449 0.0514 6833 -2.819 0.0386

## (In-labor) - (Pre-labor) 38 0.1791 0.0533 6833 3.361 0.0070

## (In-labor) - Vaginal 38 0.0118 0.0498 6833 0.237 1.0000

## (Pre-labor) - Vaginal 38 -0.1673 0.0304 6833 -5.496 <.0001

## (In-labor) - (Pre-labor) 39 0.2078 0.0491 6833 4.232 0.0002

## (In-labor) - Vaginal 39 0.0278 0.0426 6833 0.653 1.0000

## (Pre-labor) - Vaginal 39 -0.1800 0.0300 6833 -6.004 <.0001

## (In-labor) - (Pre-labor) 40+ 0.0944 0.0714 6833 1.322 1.0000

## (In-labor) - Vaginal 40+ -0.0279 0.0240 6833 -1.164 1.0000

## (Pre-labor) - Vaginal 40+ -0.1223 0.0681 6833 -1.797 0.5067

##

## P value adjustment: holm method for 12 tests

gest_type(bio="logIL18")

## $`Vaginal-inlabor`

## Anova Table (Type III tests)

##

## Response: m_planlagt[[bio]]

## Sum Sq Df F value Pr(>F)

## (Intercept) 554.34 1 3083.4011 < 2.2e-16 ***

## AGE_AT_COLLECTION 0.06 1 0.3398 0.5599586

## ALDER_MODER 0.01 1 0.0302 0.8620984

## BMI_MODER 0.54 1 2.9905 0.0838017 .

## gest 3.09 3 5.7245 0.0006579 ***

## Birth_type 0.04 1 0.2122 0.6450486

## Residuals 1109.61 6172

## ---

## Signif. codes: 0 '***' 0.001 '**' 0.01 '*' 0.05 '.' 0.1 ' ' 1

##

## $`Vaginal-prelabor`

## Anova Table (Type III tests)

##

## Response: m_under[[bio]]

## Sum Sq Df F value Pr(>F)

## (Intercept) 547.97 1 3090.9129 < 2.2e-16 ***

## AGE_AT_COLLECTION 0.00 1 0.0006 0.981252

## ALDER_MODER 0.04 1 0.2283 0.632804

## BMI_MODER 0.51 1 2.9018 0.088531 .

## gest 2.79 3 5.2427 0.001300 **

## Birth_type 1.40 1 7.8987 0.004962 **

## Residuals 1109.62 6259

## ---

## Signif. codes: 0 '***' 0.001 '**' 0.01 '*' 0.05 '.' 0.1 ' ' 1

##

## $`Inlabor-prelabor`

## Anova Table (Type III tests)

##

## Response: m_vaginalt[[bio]]

## Sum Sq Df F value Pr(>F)

## (Intercept) 111.279 1 576.3150 <2e-16 ***

## AGE_AT_COLLECTION 0.228 1 1.1818 0.2772

## ALDER_MODER 0.011 1 0.0579 0.8099

## BMI_MODER 0.072 1 0.3746 0.5406

## gest 0.568 3 0.9807 0.4010

## Birth_type 0.206 1 1.0683 0.3015

## Residuals 239.621 1241

## ---

## Signif. codes: 0 '***' 0.001 '**' 0.01 '*' 0.05 '.' 0.1 ' ' 1

##

## $`Pairwise test`

## contrast gest estimate SE df t.ratio p.value

## (In-labor) - (Pre-labor) 37 0.08109 0.0825 6833 0.983 1.0000

## (In-labor) - Vaginal 37 0.07608 0.0743 6833 1.024 1.0000

## (Pre-labor) - Vaginal 37 -0.00501 0.0510 6833 -0.098 1.0000

## (In-labor) - (Pre-labor) 38 -0.00324 0.0529 6833 -0.061 1.0000

## (In-labor) - Vaginal 38 -0.05035 0.0495 6833 -1.018 1.0000

## (Pre-labor) - Vaginal 38 -0.04710 0.0302 6833 -1.559 1.0000

## (In-labor) - (Pre-labor) 39 0.06175 0.0487 6833 1.267 1.0000

## (In-labor) - Vaginal 39 -0.01194 0.0422 6833 -0.283 1.0000

## (Pre-labor) - Vaginal 39 -0.07369 0.0298 6833 -2.476 0.1597

## (In-labor) - (Pre-labor) 40+ 0.04368 0.0709 6833 0.616 1.0000

## (In-labor) - Vaginal 40+ -0.00679 0.0238 6833 -0.286 1.0000

## (Pre-labor) - Vaginal 40+ -0.05047 0.0676 6833 -0.747 1.0000

##

## P value adjustment: holm method for 12 tests

gest_type(bio="logHSP70")

## $`Vaginal-inlabor`

## Anova Table (Type III tests)

##

## Response: m_planlagt[[bio]]

## Sum Sq Df F value Pr(>F)

## (Intercept) 8189.2 1 87876.4290 < 2.2e-16 ***

## AGE_AT_COLLECTION 0.1 1 1.5743 0.2096347

## ALDER_MODER 0.1 1 0.5946 0.4406748

## BMI_MODER 0.7 1 7.0132 0.0081118 **

## gest 1.8 3 6.6002 0.0001894 ***

## Birth_type 0.1 1 0.9078 0.3407407

## Residuals 575.2 6172

## ---

## Signif. codes: 0 '***' 0.001 '**' 0.01 '*' 0.05 '.' 0.1 ' ' 1

##

## $`Vaginal-prelabor`

## Anova Table (Type III tests)

##

## Response: m_under[[bio]]

## Sum Sq Df F value Pr(>F)

## (Intercept) 8400.3 1 90952.6749 < 2.2e-16 ***

## AGE_AT_COLLECTION 0.1 1 1.6148 0.203860

## ALDER_MODER 0.0 1 0.1760 0.674879

## BMI_MODER 0.6 1 6.8400 0.008935 **

## gest 2.3 3 8.4022 1.431e-05 ***

## Birth_type 3.9 1 42.6430 7.086e-11 ***

## Residuals 578.1 6259

## ---

## Signif. codes: 0 '***' 0.001 '**' 0.01 '*' 0.05 '.' 0.1 ' ' 1

##

## $`Inlabor-prelabor`

## Anova Table (Type III tests)

##

## Response: m_vaginalt[[bio]]

## Sum Sq Df F value Pr(>F)

## (Intercept) 1650.60 1 18620.5819 < 2.2e-16 ***

## AGE_AT_COLLECTION 0.03 1 0.3540 0.5520

## ALDER_MODER 0.04 1 0.4138 0.5201

## BMI_MODER 0.13 1 1.5064 0.2199

## gest 0.08 3 0.3083 0.8194

## Birth_type 1.48 1 16.7348 4.576e-05 ***

## Residuals 110.01 1241

## ---

## Signif. codes: 0 '***' 0.001 '**' 0.01 '*' 0.05 '.' 0.1 ' ' 1

##

## $`Pairwise test`

## contrast gest estimate SE df t.ratio p.value

## (In-labor) - (Pre-labor) 37 -0.0120 0.0591 6833 -0.203 1.0000

## (In-labor) - Vaginal 37 -0.0741 0.0532 6833 -1.392 1.0000

## (Pre-labor) - Vaginal 37 -0.0621 0.0366 6833 -1.698 0.7160

## (In-labor) - (Pre-labor) 38 0.0762 0.0379 6833 2.010 0.4003

## (In-labor) - Vaginal 38 -0.0276 0.0354 6833 -0.777 1.0000

## (Pre-labor) - Vaginal 38 -0.1037 0.0217 6833 -4.791 <.0001

## (In-labor) - (Pre-labor) 39 0.1363 0.0349 6833 3.903 0.0010

## (In-labor) - Vaginal 39 0.0434 0.0303 6833 1.433 1.0000

## (Pre-labor) - Vaginal 39 -0.0929 0.0213 6833 -4.357 0.0001

## (In-labor) - (Pre-labor) 40+ 0.0649 0.0508 6833 1.277 1.0000

## (In-labor) - Vaginal 40+ 0.0212 0.0170 6833 1.242 1.0000

## (Pre-labor) - Vaginal 40+ -0.0437 0.0484 6833 -0.903 1.0000

##

## P value adjustment: holm method for 12 tests

gest_type(bio="logSTNF_RI")

## $`Vaginal-inlabor`

## Anova Table (Type III tests)

##

## Response: m_planlagt[[bio]]

## Sum Sq Df F value Pr(>F)

## (Intercept) 1411.61 1 4900.5087 < 2.2e-16 ***

## AGE_AT_COLLECTION 38.96 1 135.2676 < 2.2e-16 ***

## ALDER_MODER 2.27 1 7.8705 0.0050406 **

## BMI_MODER 3.44 1 11.9569 0.0005481 ***

## gest 1.62 3 1.8778 0.1309885

## Birth_type 1.07 1 3.7129 0.0540383 .

## Residuals 1777.87 6172

## ---

## Signif. codes: 0 '***' 0.001 '**' 0.01 '*' 0.05 '.' 0.1 ' ' 1

##

## $`Vaginal-prelabor`

## Anova Table (Type III tests)

##

## Response: m_under[[bio]]

## Sum Sq Df F value Pr(>F)

## (Intercept) 1451.36 1 5112.0165 < 2.2e-16 ***

## AGE_AT_COLLECTION 41.14 1 144.9044 < 2.2e-16 ***

## ALDER_MODER 2.32 1 8.1760 0.004259 **

## BMI_MODER 2.64 1 9.2980 0.002304 **

## gest 2.67 3 3.1368 0.024374 *

## Birth_type 0.00 1 0.0077 0.930195

## Residuals 1777.01 6259

## ---

## Signif. codes: 0 '***' 0.001 '**' 0.01 '*' 0.05 '.' 0.1 ' ' 1

##

## $`Inlabor-prelabor`

## Anova Table (Type III tests)

##

## Response: m_vaginalt[[bio]]

## Sum Sq Df F value Pr(>F)

## (Intercept) 257.40 1 909.5693 < 2.2e-16 ***

## AGE_AT_COLLECTION 2.76 1 9.7421 0.001843 **

## ALDER_MODER 0.14 1 0.4808 0.488196

## BMI_MODER 0.02 1 0.0777 0.780497

## gest 2.03 3 2.3954 0.066723 .

## Birth_type 0.09 1 0.3097 0.577943

## Residuals 351.19 1241

## ---

## Signif. codes: 0 '***' 0.001 '**' 0.01 '*' 0.05 '.' 0.1 ' ' 1

##

## $`Pairwise test`

## contrast gest estimate SE df t.ratio p.value

## (In-labor) - (Pre-labor) 37 0.01312 0.1040 6833 0.126 1.0000

## (In-labor) - Vaginal 37 0.06316 0.0936 6833 0.675 1.0000

## (Pre-labor) - Vaginal 37 0.05004 0.0643 6833 0.778 1.0000

## (In-labor) - (Pre-labor) 38 0.01716 0.0667 6833 0.257 1.0000

## (In-labor) - Vaginal 38 0.03791 0.0624 6833 0.608 1.0000

## (Pre-labor) - Vaginal 38 0.02075 0.0381 6833 0.545 1.0000

## (In-labor) - (Pre-labor) 39 0.04711 0.0614 6833 0.767 1.0000

## (In-labor) - Vaginal 39 0.00282 0.0533 6833 0.053 1.0000

## (Pre-labor) - Vaginal 39 -0.04429 0.0375 6833 -1.181 1.0000

## (In-labor) - (Pre-labor) 40+ 0.03049 0.0894 6833 0.341 1.0000

## (In-labor) - Vaginal 40+ 0.05742 0.0300 6833 1.915 0.6656

## (Pre-labor) - Vaginal 40+ 0.02692 0.0852 6833 0.316 1.0000

##

## P value adjustment: holm method for 12 tests

gest_type(bio="logEGF")

## $`Vaginal-inlabor`

## Anova Table (Type III tests)

##

## Response: m_planlagt[[bio]]

## Sum Sq Df F value Pr(>F)

## (Intercept) 542.88 1 2509.9240 < 2.2e-16 ***

## AGE_AT_COLLECTION 2.43 1 11.2538 0.0007994 ***

## ALDER_MODER 0.66 1 3.0466 0.0809557 .

## BMI_MODER 0.66 1 3.0654 0.0800234 .

## gest 0.49 3 0.7561 0.5186505

## Birth_type 0.27 1 1.2462 0.2643239

## Residuals 1334.97 6172

## ---

## Signif. codes: 0 '***' 0.001 '**' 0.01 '*' 0.05 '.' 0.1 ' ' 1

##

## $`Vaginal-prelabor`

## Anova Table (Type III tests)

##

## Response: m_under[[bio]]

## Sum Sq Df F value Pr(>F)

## (Intercept) 568.25 1 2684.1332 < 2.2e-16 ***

## AGE_AT_COLLECTION 2.22 1 10.4834 0.001211 **

## ALDER_MODER 0.41 1 1.9519 0.162434

## BMI_MODER 0.61 1 2.8993 0.088665 .

## gest 0.58 3 0.9064 0.437036

## Birth_type 0.87 1 4.0994 0.042941 *

## Residuals 1325.07 6259

## ---

## Signif. codes: 0 '***' 0.001 '**' 0.01 '*' 0.05 '.' 0.1 ' ' 1

##

## $`Inlabor-prelabor`

## Anova Table (Type III tests)

##

## Response: m_vaginalt[[bio]]

## Sum Sq Df F value Pr(>F)

## (Intercept) 100.718 1 508.5052 <2e-16 ***

## AGE_AT_COLLECTION 0.112 1 0.5652 0.4523

## ALDER_MODER 0.057 1 0.2858 0.5930

## BMI_MODER 0.016 1 0.0800 0.7773

## gest 1.196 3 2.0126 0.1104

## Birth_type 0.002 1 0.0100 0.9205

## Residuals 245.801 1241

## ---

## Signif. codes: 0 '***' 0.001 '**' 0.01 '*' 0.05 '.' 0.1 ' ' 1

##

## $`Pairwise test`

## contrast gest estimate SE df t.ratio p.value

## (In-labor) - (Pre-labor) 37 -0.03738 0.0897 6833 -0.417 1.0000

## (In-labor) - Vaginal 37 0.03522 0.0807 6833 0.436 1.0000

## (Pre-labor) - Vaginal 37 0.07260 0.0555 6833 1.309 1.0000

## (In-labor) - (Pre-labor) 38 0.03019 0.0575 6833 0.525 1.0000

## (In-labor) - Vaginal 38 0.09523 0.0538 6833 1.771 0.8429

## (Pre-labor) - Vaginal 38 0.06504 0.0328 6833 1.980 0.5725

## (In-labor) - (Pre-labor) 39 0.02298 0.0530 6833 0.434 1.0000

## (In-labor) - Vaginal 39 0.02694 0.0459 6833 0.587 1.0000

## (Pre-labor) - Vaginal 39 0.00396 0.0323 6833 0.122 1.0000

## (In-labor) - (Pre-labor) 40+ -0.06568 0.0771 6833 -0.852 1.0000

## (In-labor) - Vaginal 40+ 0.00290 0.0258 6833 0.112 1.0000

## (Pre-labor) - Vaginal 40+ 0.06857 0.0734 6833 0.934 1.0000

##

## P value adjustment: holm method for 12 tests

gest_type(bio="logVEGF")

## $`Vaginal-inlabor`

## Anova Table (Type III tests)

##

## Response: m_planlagt[[bio]]

## Sum Sq Df F value Pr(>F)

## (Intercept) 931.35 1 4768.4852 < 2e-16 ***

## AGE_AT_COLLECTION 40.16 1 205.6040 < 2e-16 ***

## ALDER_MODER 0.38 1 1.9702 0.16048

## BMI_MODER 0.88 1 4.5184 0.03357 *

## gest 0.51 3 0.8717 0.45489

## Birth_type 0.89 1 4.5403 0.03315 *

## Residuals 1205.48 6172

## ---

## Signif. codes: 0 '***' 0.001 '**' 0.01 '*' 0.05 '.' 0.1 ' ' 1

##

## $`Vaginal-prelabor`

## Anova Table (Type III tests)

##

## Response: m_under[[bio]]

## Sum Sq Df F value Pr(>F)

## (Intercept) 998.00 1 5163.3942 < 2.2e-16 ***

## AGE_AT_COLLECTION 44.55 1 230.5044 < 2.2e-16 ***

## ALDER_MODER 0.47 1 2.4238 0.11956

## BMI_MODER 0.54 1 2.8150 0.09344 .

## gest 0.74 3 1.2778 0.28011

## Birth_type 2.99 1 15.4502 8.562e-05 ***

## Residuals 1209.76 6259

## ---

## Signif. codes: 0 '***' 0.001 '**' 0.01 '*' 0.05 '.' 0.1 ' ' 1

##

## $`Inlabor-prelabor`

## Anova Table (Type III tests)

##

## Response: m_vaginalt[[bio]]

## Sum Sq Df F value Pr(>F)

## (Intercept) 173.917 1 906.4322 < 2.2e-16 ***

## AGE_AT_COLLECTION 7.303 1 38.0624 9.258e-10 ***

## ALDER_MODER 0.091 1 0.4728 0.4918

## BMI_MODER 0.011 1 0.0556 0.8136

## gest 0.877 3 1.5228 0.2068

## Birth_type 0.289 1 1.5053 0.2201

## Residuals 238.111 1241

## ---

## Signif. codes: 0 '***' 0.001 '**' 0.01 '*' 0.05 '.' 0.1 ' ' 1

##

## $`Pairwise test`

## contrast gest estimate SE df t.ratio p.value

## (In-labor) - (Pre-labor) 37 -0.03025 0.0857 6833 -0.353 1.0000

## (In-labor) - Vaginal 37 0.03966 0.0772 6833 0.514 1.0000

## (Pre-labor) - Vaginal 37 0.06992 0.0530 6833 1.319 1.0000

## (In-labor) - (Pre-labor) 38 -0.07040 0.0549 6833 -1.281 1.0000

## (In-labor) - Vaginal 38 0.04752 0.0514 6833 0.925 1.0000

## (Pre-labor) - Vaginal 38 0.11792 0.0314 6833 3.757 0.0021

## (In-labor) - (Pre-labor) 39 -0.00243 0.0506 6833 -0.048 1.0000

## (In-labor) - Vaginal 39 0.02786 0.0439 6833 0.635 1.0000

## (Pre-labor) - Vaginal 39 0.03030 0.0309 6833 0.980 1.0000

## (In-labor) - (Pre-labor) 40+ -0.09356 0.0736 6833 -1.270 1.0000

## (In-labor) - Vaginal 40+ 0.04364 0.0247 6833 1.767 0.7733

## (Pre-labor) - Vaginal 40+ 0.13720 0.0702 6833 1.955 0.5569

##

## P value adjustment: holm method for 12 tests

gest_type(bio="logS100B")

## $`Vaginal-inlabor`

## Anova Table (Type III tests)

##

## Response: m_planlagt[[bio]]

## Sum Sq Df F value Pr(>F)

## (Intercept) 1820.39 1 5998.7401 < 2e-16 ***

## AGE_AT_COLLECTION 0.37 1 1.2203 0.26935

## ALDER_MODER 0.25 1 0.8307 0.36209

## BMI_MODER 0.08 1 0.2797 0.59694

## gest 1.76 3 1.9290 0.12256

## Birth_type 0.93 1 3.0601 0.08029 .

## Residuals 1872.97 6172

## ---

## Signif. codes: 0 '***' 0.001 '**' 0.01 '*' 0.05 '.' 0.1 ' ' 1

##

## $`Vaginal-prelabor`

## Anova Table (Type III tests)

##

## Response: m_under[[bio]]

## Sum Sq Df F value Pr(>F)

## (Intercept) 1903.13 1 6233.3674 < 2e-16 ***

## AGE_AT_COLLECTION 0.15 1 0.4777 0.48948

## ALDER_MODER 0.02 1 0.0580 0.80975

## BMI_MODER 0.08 1 0.2735 0.60102

## gest 1.80 3 1.9666 0.11671

## Birth_type 1.39 1 4.5640 0.03269 *

## Residuals 1910.96 6259

## ---

## Signif. codes: 0 '***' 0.001 '**' 0.01 '*' 0.05 '.' 0.1 ' ' 1

##

## $`Inlabor-prelabor`

## Anova Table (Type III tests)

##

## Response: m_vaginalt[[bio]]

## Sum Sq Df F value Pr(>F)

## (Intercept) 336.60 1 1039.4265 < 2e-16 ***

## AGE_AT_COLLECTION 2.04 1 6.3059 0.01216 *

## ALDER_MODER 0.18 1 0.5544 0.45665

## BMI_MODER 0.05 1 0.1608 0.68846

## gest 0.94 3 0.9661 0.40795

## Birth_type 0.37 1 1.1467 0.28446

## Residuals 401.87 1241

## ---

## Signif. codes: 0 '***' 0.001 '**' 0.01 '*' 0.05 '.' 0.1 ' ' 1

##

## $`Pairwise test`

## contrast gest estimate SE df t.ratio p.value

## (In-labor) - (Pre-labor) 37 -0.04253 0.1076 6833 -0.395 1.0000

## (In-labor) - Vaginal 37 0.07918 0.0969 6833 0.817 1.0000

## (Pre-labor) - Vaginal 37 0.12171 0.0666 6833 1.828 0.7432

## (In-labor) - (Pre-labor) 38 -0.02317 0.0690 6833 -0.336 1.0000

## (In-labor) - Vaginal 38 0.03347 0.0646 6833 0.518 1.0000

## (Pre-labor) - Vaginal 38 0.05664 0.0394 6833 1.437 1.0000

## (In-labor) - (Pre-labor) 39 -0.00248 0.0636 6833 -0.039 1.0000

## (In-labor) - Vaginal 39 0.00744 0.0551 6833 0.135 1.0000

## (Pre-labor) - Vaginal 39 0.00992 0.0388 6833 0.255 1.0000

## (In-labor) - (Pre-labor) 40+ -0.11612 0.0925 6833 -1.255 1.0000

## (In-labor) - Vaginal 40+ 0.05089 0.0310 6833 1.640 1.0000

## (Pre-labor) - Vaginal 40+ 0.16701 0.0882 6833 1.894 0.6985

##

## P value adjustment: holm method for 12 tests

gest_type(bio="logBDNF")

## $`Vaginal-inlabor`

## Anova Table (Type III tests)

##

## Response: m_planlagt[[bio]]

## Sum Sq Df F value Pr(>F)

## (Intercept) 2047.6 1 3678.3995 < 2.2e-16 ***

## AGE_AT_COLLECTION 4.8 1 8.5689 0.003432 **

## ALDER_MODER 3.4 1 6.0900 0.013622 *

## BMI_MODER 5.4 1 9.7349 0.001816 **

## gest 1.5 3 0.8830 0.449025

## Birth_type 0.0 1 0.0138 0.906422

## Residuals 3435.7 6172

## ---

## Signif. codes: 0 '***' 0.001 '**' 0.01 '*' 0.05 '.' 0.1 ' ' 1

##

## $`Vaginal-prelabor`

## Anova Table (Type III tests)

##

## Response: m_under[[bio]]

## Sum Sq Df F value Pr(>F)

## (Intercept) 2184.6 1 3993.0905 < 2.2e-16 ***

## AGE_AT_COLLECTION 4.5 1 8.2311 0.004132 **

## ALDER_MODER 4.6 1 8.4322 0.003699 **

## BMI_MODER 4.7 1 8.6273 0.003324 **

## gest 0.8 3 0.4989 0.683056

## Birth_type 0.9 1 1.6154 0.203778

## Residuals 3424.2 6259

## ---

## Signif. codes: 0 '***' 0.001 '**' 0.01 '*' 0.05 '.' 0.1 ' ' 1

##

## $`Inlabor-prelabor`

## Anova Table (Type III tests)

##

## Response: m_vaginalt[[bio]]

## Sum Sq Df F value Pr(>F)

## (Intercept) 394.49 1 701.9739 <2e-16 ***

## AGE_AT_COLLECTION 0.08 1 0.1469 0.7016

## ALDER_MODER 0.43 1 0.7652 0.3819

## BMI_MODER 0.46 1 0.8227 0.3646

## gest 1.25 3 0.7406 0.5279

## Birth_type 0.85 1 1.5170 0.2183

## Residuals 697.41 1241

## ---

## Signif. codes: 0 '***' 0.001 '**' 0.01 '*' 0.05 '.' 0.1 ' ' 1

##

## $`Pairwise test`

## contrast gest estimate SE df t.ratio p.value

## (In-labor) - (Pre-labor) 37 -0.1652 0.1446 6833 -1.142 1.0000

## (In-labor) - Vaginal 37 -0.1066 0.1302 6833 -0.819 1.0000

## (Pre-labor) - Vaginal 37 0.0586 0.0894 6833 0.655 1.0000

## (In-labor) - (Pre-labor) 38 0.0472 0.0927 6833 0.510 1.0000

## (In-labor) - Vaginal 38 0.0709 0.0867 6833 0.817 1.0000

## (Pre-labor) - Vaginal 38 0.0236 0.0530 6833 0.446 1.0000

## (In-labor) - (Pre-labor) 39 -0.0683 0.0854 6833 -0.800 1.0000

## (In-labor) - Vaginal 39 -0.0400 0.0741 6833 -0.541 1.0000

## (Pre-labor) - Vaginal 39 0.0283 0.0522 6833 0.542 1.0000

## (In-labor) - (Pre-labor) 40+ -0.1627 0.1243 6833 -1.309 1.0000

## (In-labor) - Vaginal 40+ 0.0130 0.0417 6833 0.313 1.0000

## (Pre-labor) - Vaginal 40+ 0.1758 0.1184 6833 1.484 1.0000

##

## P value adjustment: holm method for 12 tests

gest_type(bio="logNT3")

## $`Vaginal-inlabor`

## Anova Table (Type III tests)

##

## Response: m_planlagt[[bio]]

## Sum Sq Df F value Pr(>F)

## (Intercept) 117.71 1 478.1425 < 2e-16 ***

## AGE_AT_COLLECTION 5.15 1 20.9128 4.9e-06 ***

## ALDER_MODER 1.00 1 4.0715 0.04366 *

## BMI_MODER 0.50 1 2.0311 0.15416

## gest 0.69 3 0.9313 0.42459

## Birth_type 0.44 1 1.7676 0.18373

## Residuals 1519.47 6172

## ---

## Signif. codes: 0 '***' 0.001 '**' 0.01 '*' 0.05 '.' 0.1 ' ' 1

##

## $`Vaginal-prelabor`

## Anova Table (Type III tests)

##

## Response: m_under[[bio]]

## Sum Sq Df F value Pr(>F)

## (Intercept) 121.73 1 503.3664 < 2e-16 ***

## AGE_AT_COLLECTION 5.02 1 20.7619 5.3e-06 ***

## ALDER_MODER 0.92 1 3.8226 0.05061 .

## BMI_MODER 0.61 1 2.5050 0.11354

## gest 0.60 3 0.8257 0.47949

## Birth_type 0.03 1 0.1192 0.72990

## Residuals 1513.66 6259

## ---

## Signif. codes: 0 '***' 0.001 '**' 0.01 '*' 0.05 '.' 0.1 ' ' 1

##

## $`Inlabor-prelabor`

## Anova Table (Type III tests)

##

## Response: m_vaginalt[[bio]]

## Sum Sq Df F value Pr(>F)

## (Intercept) 14.887 1 67.1052 6.343e-16 ***

## AGE_AT_COLLECTION 0.226 1 1.0196 0.3128

## ALDER_MODER 0.008 1 0.0373 0.8470

## BMI_MODER 0.125 1 0.5636 0.4529

## gest 0.904 3 1.3585 0.2540

## Birth_type 0.000 1 0.0003 0.9873

## Residuals 275.309 1241

## ---

## Signif. codes: 0 '***' 0.001 '**' 0.01 '*' 0.05 '.' 0.1 ' ' 1

##

## $`Pairwise test`

## contrast gest estimate SE df t.ratio p.value

## (In-labor) - (Pre-labor) 37 -0.0986 0.0957 6833 -1.031 1.0000

## (In-labor) - Vaginal 37 -0.0627 0.0862 6833 -0.728 1.0000

## (Pre-labor) - Vaginal 37 0.0359 0.0592 6833 0.607 1.0000

## (In-labor) - (Pre-labor) 38 0.0174 0.0614 6833 0.283 1.0000

## (In-labor) - Vaginal 38 0.0522 0.0574 6833 0.909 1.0000

## (Pre-labor) - Vaginal 38 0.0348 0.0351 6833 0.993 1.0000

## (In-labor) - (Pre-labor) 39 0.0660 0.0565 6833 1.167 1.0000

## (In-labor) - Vaginal 39 0.0304 0.0490 6833 0.621 1.0000

## (Pre-labor) - Vaginal 39 -0.0356 0.0345 6833 -1.030 1.0000

## (In-labor) - (Pre-labor) 40+ -0.0264 0.0823 6833 -0.321 1.0000

## (In-labor) - Vaginal 40+ 0.0320 0.0276 6833 1.159 1.0000

## (Pre-labor) - Vaginal 40+ 0.0584 0.0784 6833 0.745 1.0000

##

## P value adjustment: holm method for 12 tests

S3.4 Testing gender

Figure 2 in the paper

Splitting the dataset in boys and girls to get an overall p-value for each of the genders

Testing

koen_type(bio="logCRP")

## $Boys

## Anova Table (Type III tests)

##

## Response: boys[[bio]]

## Sum Sq Df F value Pr(>F)

## (Intercept) 27.99 1 50.7986 1.225e-12 ***

## AGE_AT_COLLECTION 9.95 1 18.0664 2.186e-05 ***

## ALDER_MODER 0.84 1 1.5180 0.2180

## BMI_MODER 0.52 1 0.9527 0.3291

## Birth_type 66.38 2 60.2340 < 2.2e-16 ***

## Residuals 2038.66 3700

## ---

## Signif. codes: 0 '***' 0.001 '**' 0.01 '*' 0.05 '.' 0.1 ' ' 1

##

## $Girls

## Anova Table (Type III tests)

##

## Response: girls[[bio]]

## Sum Sq Df F value Pr(>F)

## (Intercept) 28.98 1 53.0660 4.061e-13 ***

## AGE_AT_COLLECTION 9.09 1 16.6379 4.636e-05 ***

## ALDER_MODER 3.45 1 6.3170 0.01201 *

## BMI_MODER 1.60 1 2.9355 0.08675 .

## Birth_type 96.34 2 88.2081 < 2.2e-16 ***

## Residuals 1712.48 3136

## ---

## Signif. codes: 0 '***' 0.001 '**' 0.01 '*' 0.05 '.' 0.1 ' ' 1

##

## $`Pairwise test`

## contrast Birth_type estimate SE df t.ratio p.value

## K - M In-labor -0.190 0.0625 6839 -3.033 0.0024

## K - M Pre-labor -0.272 0.0575 6839 -4.739 <.0001

## K - M Vaginal -0.142 0.0199 6839 -7.132 <.0001

##

## P value adjustment: holm method for 3 tests

koen_type(bio="logMCP1")

## $Boys

## Anova Table (Type III tests)

##

## Response: boys[[bio]]

## Sum Sq Df F value Pr(>F)

## (Intercept) 1388.94 1 7406.4208 <2e-16 ***

## AGE_AT_COLLECTION 24.96 1 133.0840 <2e-16 ***

## ALDER_MODER 0.02 1 0.1234 0.7254

## BMI_MODER 0.04 1 0.2324 0.6298

## Birth_type 15.97 2 42.5848 <2e-16 ***

## Residuals 693.87 3700

## ---

## Signif. codes: 0 '***' 0.001 '**' 0.01 '*' 0.05 '.' 0.1 ' ' 1

##

## $Girls

## Anova Table (Type III tests)

##

## Response: girls[[bio]]

## Sum Sq Df F value Pr(>F)

## (Intercept) 1157.89 1 6535.6612 < 2.2e-16 ***

## AGE_AT_COLLECTION 27.23 1 153.6903 < 2.2e-16 ***

## ALDER_MODER 0.56 1 3.1517 0.07595 .

## BMI_MODER 0.03 1 0.1610 0.68829

## Birth_type 12.34 2 34.8189 1.106e-15 ***

## Residuals 555.59 3136

## ---

## Signif. codes: 0 '***' 0.001 '**' 0.01 '*' 0.05 '.' 0.1 ' ' 1

##

## $`Pairwise test`

## contrast Birth_type estimate SE df t.ratio p.value

## K - M In-labor -0.0433 0.0361 6839 -1.200 0.2302

## K - M Pre-labor -0.0588 0.0332 6839 -1.772 0.1529

## K - M Vaginal -0.0749 0.0115 6839 -6.532 <.0001

##

## P value adjustment: holm method for 3 tests

koen_type(bio="logIL18")

## $Boys

## Anova Table (Type III tests)

##

## Response: boys[[bio]]

## Sum Sq Df F value Pr(>F)

## (Intercept) 414.14 1 2276.9402 < 2.2e-16 ***

## AGE_AT_COLLECTION 0.02 1 0.1151 0.734410

## ALDER_MODER 0.00 1 0.0221 0.881877

## BMI_MODER 0.62 1 3.4262 0.064249 .

## Birth_type 1.86 2 5.1138 0.006056 **

## Residuals 672.97 3700

## ---

## Signif. codes: 0 '***' 0.001 '**' 0.01 '*' 0.05 '.' 0.1 ' ' 1

##

## $Girls

## Anova Table (Type III tests)

##

## Response: girls[[bio]]

## Sum Sq Df F value Pr(>F)

## (Intercept) 334.64 1 1876.1914 < 2e-16 ***

## AGE_AT_COLLECTION 0.06 1 0.3242 0.56913

## ALDER_MODER 0.05 1 0.2773 0.59852

## BMI_MODER 0.10 1 0.5795 0.44655

## Birth_type 1.62 2 4.5363 0.01078 *

## Residuals 559.34 3136

## ---

## Signif. codes: 0 '***' 0.001 '**' 0.01 '*' 0.05 '.' 0.1 ' ' 1

##

## $`Pairwise test`

## contrast Birth_type estimate SE df t.ratio p.value

## K - M In-labor -0.01841 0.0358 6839 -0.514 1.0000

## K - M Pre-labor -0.00959 0.0329 6839 -0.291 1.0000

## K - M Vaginal -0.01407 0.0114 6839 -1.236 0.6494

##

## P value adjustment: holm method for 3 tests

koen_type(bio="logHSP70")

## $Boys

## Anova Table (Type III tests)

##

## Response: boys[[bio]]

## Sum Sq Df F value Pr(>F)

## (Intercept) 5992.0 1 66267.5461 < 2e-16 ***

## AGE_AT_COLLECTION 0.3 1 2.9686 0.08498 .

## ALDER_MODER 0.1 1 0.5979 0.43944

## BMI_MODER 0.4 1 4.1572 0.04153 *

## Birth_type 0.8 2 4.5286 0.01086 *

## Residuals 334.6 3700

## ---

## Signif. codes: 0 '***' 0.001 '**' 0.01 '*' 0.05 '.' 0.1 ' ' 1

##

## $Girls

## Anova Table (Type III tests)

##

## Response: girls[[bio]]

## Sum Sq Df F value Pr(>F)

## (Intercept) 4876.0 1 51261.9555 < 2.2e-16 ***

## AGE_AT_COLLECTION 0.0 1 0.0346 0.85255

## ALDER_MODER 0.3 1 2.8129 0.09361 .

## BMI_MODER 0.3 1 2.8487 0.09155 .

## Birth_type 2.3 2 11.9295 6.898e-06 ***

## Residuals 298.3 3136

## ---

## Signif. codes: 0 '***' 0.001 '**' 0.01 '*' 0.05 '.' 0.1 ' ' 1

##

## $`Pairwise test`

## contrast Birth_type estimate SE df t.ratio p.value

## K - M In-labor 0.0163 0.02568 6839 0.633 0.5268

## K - M Pre-labor -0.0347 0.02361 6839 -1.472 0.2824

## K - M Vaginal 0.0140 0.00816 6839 1.721 0.2557

##

## P value adjustment: holm method for 3 tests

koen_type(bio="logSTNF_RI")

## $Boys

## Anova Table (Type III tests)

##

## Response: boys[[bio]]

## Sum Sq Df F value Pr(>F)

## (Intercept) 959.06 1 3441.5015 < 2.2e-16 ***

## AGE_AT_COLLECTION 18.50 1 66.3796 5.036e-16 ***

## ALDER_MODER 0.27 1 0.9571 0.3280

## BMI_MODER 0.39 1 1.4002 0.2368

## Birth_type 1.20 2 2.1520 0.1164

## Residuals 1031.10 3700

## ---

## Signif. codes: 0 '***' 0.001 '**' 0.01 '*' 0.05 '.' 0.1 ' ' 1

##

## $Girls

## Anova Table (Type III tests)

##

## Response: girls[[bio]]

## Sum Sq Df F value Pr(>F)

## (Intercept) 896.14 1 3055.5714 < 2.2e-16 ***

## AGE_AT_COLLECTION 22.24 1 75.8482 < 2.2e-16 ***

## ALDER_MODER 3.01 1 10.2592 0.0013737 **

## BMI_MODER 3.66 1 12.4701 0.0004195 ***

## Birth_type 0.83 2 1.4076 0.2448827

## Residuals 919.72 3136

## ---

## Signif. codes: 0 '***' 0.001 '**' 0.01 '*' 0.05 '.' 0.1 ' ' 1

##

## $`Pairwise test`

## contrast Birth_type estimate SE df t.ratio p.value

## K - M In-labor 0.0575 0.0451 6839 1.275 0.4046

## K - M Pre-labor -0.0212 0.0415 6839 -0.512 0.6090

## K - M Vaginal 0.0490 0.0143 6839 3.422 0.0019

##

## P value adjustment: holm method for 3 tests

koen_type(bio="logEGF")

## $Boys

## Anova Table (Type III tests)

##

## Response: boys[[bio]]

## Sum Sq Df F value Pr(>F)

## (Intercept) 360.10 1 1672.9722 < 2e-16 ***

## AGE_AT_COLLECTION 1.08 1 5.0291 0.02498 *

## ALDER_MODER 0.00 1 0.0007 0.97922

## BMI_MODER 0.08 1 0.3674 0.54444

## Birth_type 0.57 2 1.3188 0.26757

## Residuals 796.41 3700

## ---

## Signif. codes: 0 '***' 0.001 '**' 0.01 '*' 0.05 '.' 0.1 ' ' 1

##

## $Girls

## Anova Table (Type III tests)

##

## Response: girls[[bio]]

## Sum Sq Df F value Pr(>F)

## (Intercept) 343.98 1 1656.5422 < 2e-16 ***

## AGE_AT_COLLECTION 1.17 1 5.6557 0.01746 *

## ALDER_MODER 0.99 1 4.7595 0.02921 *

## BMI_MODER 0.87 1 4.1788 0.04102 *

## Birth_type 1.23 2 2.9530 0.05233 .

## Residuals 651.19 3136

## ---

## Signif. codes: 0 '***' 0.001 '**' 0.01 '*' 0.05 '.' 0.1 ' ' 1

##

## $`Pairwise test`

## contrast Birth_type estimate SE df t.ratio p.value

## K - M In-labor 0.0833 0.0388 6839 2.145 0.0640

## K - M Pre-labor 0.0624 0.0357 6839 1.749 0.0804

## K - M Vaginal 0.0552 0.0123 6839 4.475 <.0001

##

## P value adjustment: holm method for 3 tests

koen_type(bio="logVEGF")

## $Boys

## Anova Table (Type III tests)

##

## Response: boys[[bio]]

## Sum Sq Df F value Pr(>F)

## (Intercept) 634.71 1 3313.7083 < 2.2e-16 ***

## AGE_AT_COLLECTION 21.85 1 114.0846 < 2.2e-16 ***

## ALDER_MODER 0.00 1 0.0151 0.902206

## BMI_MODER 0.03 1 0.1446 0.703809

## Birth_type 2.17 2 5.6750 0.003461 **

## Residuals 708.70 3700

## ---

## Signif. codes: 0 '***' 0.001 '**' 0.01 '*' 0.05 '.' 0.1 ' ' 1

##

## $Girls

## Anova Table (Type III tests)

##

## Response: girls[[bio]]

## Sum Sq Df F value Pr(>F)

## (Intercept) 593.20 1 3060.1258 < 2.2e-16 ***

## AGE_AT_COLLECTION 24.34 1 125.5494 < 2.2e-16 ***

## ALDER_MODER 0.65 1 3.3352 0.067906 .

## BMI_MODER 1.13 1 5.8232 0.015873 *

## Birth_type 3.25 2 8.3740 0.000236 ***

## Residuals 607.91 3136

## ---

## Signif. codes: 0 '***' 0.001 '**' 0.01 '*' 0.05 '.' 0.1 ' ' 1

##

## $`Pairwise test`

## contrast Birth_type estimate SE df t.ratio p.value

## K - M In-labor 0.0874 0.0370 6839 2.360 0.0183

## K - M Pre-labor 0.0948 0.0340 6839 2.784 0.0108

## K - M Vaginal 0.0750 0.0118 6839 6.371 <.0001

##

## P value adjustment: holm method for 3 tests

koen_type(bio="logS100B")

## $Boys

## Anova Table (Type III tests)

##

## Response: boys[[bio]]

## Sum Sq Df F value Pr(>F)

## (Intercept) 1292.28 1 4100.2609 < 2.2e-16 ***

## AGE_AT_COLLECTION 0.21 1 0.6570 0.417675

## ALDER_MODER 0.07 1 0.2302 0.631381

## BMI_MODER 0.13 1 0.4153 0.519353

## Birth_type 3.26 2 5.1738 0.005704 **

## Residuals 1166.13 3700

## ---

## Signif. codes: 0 '***' 0.001 '**' 0.01 '*' 0.05 '.' 0.1 ' ' 1

##

## $Girls

## Anova Table (Type III tests)

##

## Response: girls[[bio]]

## Sum Sq Df F value Pr(>F)

## (Intercept) 1093.50 1 3693.2305 <2e-16 ***

## AGE_AT_COLLECTION 0.51 1 1.7084 0.1913

## ALDER_MODER 0.49 1 1.6496 0.1991

## BMI_MODER 0.00 1 0.0025 0.9604

## Birth_type 1.18 2 1.9959 0.1361

## Residuals 928.51 3136

## ---

## Signif. codes: 0 '***' 0.001 '**' 0.01 '*' 0.05 '.' 0.1 ' ' 1

##

## $`Pairwise test`

## contrast Birth_type estimate SE df t.ratio p.value

## K - M In-labor 0.0304 0.0467 6839 0.650 0.7181

## K - M Pre-labor -0.0408 0.0429 6839 -0.950 0.7181

## K - M Vaginal 0.0175 0.0148 6839 1.177 0.7181

##

## P value adjustment: holm method for 3 tests

koen_type(bio="logBDNF")

## $Boys

## Anova Table (Type III tests)

##

## Response: boys[[bio]]

## Sum Sq Df F value Pr(>F)

## (Intercept) 1462.61 1 2602.3943 < 2e-16 ***

## AGE_AT_COLLECTION 1.34 1 2.3862 0.12250

## ALDER_MODER 2.00 1 3.5557 0.05942 .

## BMI_MODER 2.38 1 4.2290 0.03981 *

## Birth_type 1.22 2 1.0856 0.33780

## Residuals 2079.49 3700

## ---

## Signif. codes: 0 '***' 0.001 '**' 0.01 '*' 0.05 '.' 0.1 ' ' 1

##

## $Girls

## Anova Table (Type III tests)

##

## Response: girls[[bio]]

## Sum Sq Df F value Pr(>F)

## (Intercept) 1335.21 1 2490.4112 < 2.2e-16 ***

## AGE_AT_COLLECTION 3.76 1 7.0141 0.008127 **

## ALDER_MODER 2.36 1 4.3929 0.036170 *

## BMI_MODER 3.13 1 5.8431 0.015695 *

## Birth_type 2.35 2 2.1961 0.111402

## Residuals 1681.34 3136

## ---

## Signif. codes: 0 '***' 0.001 '**' 0.01 '*' 0.05 '.' 0.1 ' ' 1

##

## $`Pairwise test`

## contrast Birth_type estimate SE df t.ratio p.value

## K - M In-labor 0.2329 0.0626 6839 3.721 0.0004

## K - M Pre-labor 0.1026 0.0575 6839 1.784 0.0744

## K - M Vaginal 0.0842 0.0199 6839 4.234 0.0001

##

## P value adjustment: holm method for 3 tests

koen_type(bio="logNT3")

## $Boys

## Anova Table (Type III tests)

##

## Response: boys[[bio]]

## Sum Sq Df F value Pr(>F)

## (Intercept) 77.66 1 341.0588 < 2.2e-16 ***

## AGE_AT_COLLECTION 1.61 1 7.0834 0.007814 **

## ALDER_MODER 0.14 1 0.6243 0.429494

## BMI_MODER 0.08 1 0.3721 0.541896

## Birth_type 0.16 2 0.3567 0.700030

## Residuals 842.47 3700

## ---

## Signif. codes: 0 '***' 0.001 '**' 0.01 '*' 0.05 '.' 0.1 ' ' 1

##

## $Girls

## Anova Table (Type III tests)

##

## Response: girls[[bio]]

## Sum Sq Df F value Pr(>F)

## (Intercept) 84.86 1 327.6341 < 2.2e-16 ***

## AGE_AT_COLLECTION 2.85 1 11.0073 0.0009179 ***

## ALDER_MODER 0.92 1 3.5499 0.0596407 .

## BMI_MODER 0.80 1 3.0859 0.0790730 .

## Birth_type 0.88 2 1.6992 0.1829982

## Residuals 812.25 3136

## ---

## Signif. codes: 0 '***' 0.001 '**' 0.01 '*' 0.05 '.' 0.1 ' ' 1

##

## $`Pairwise test`

## contrast Birth_type estimate SE df t.ratio p.value

## K - M In-labor 0.0616 0.0415 6839 1.484 0.4136

## K - M Pre-labor -0.0269 0.0382 6839 -0.705 0.4806

## K - M Vaginal 0.0173 0.0132 6839 1.309 0.4136

##

## P value adjustment: holm method for 3 tests
