# Supplementary material for: Delivery Modality Affect Neonatal Levels of Inflammation, Stress, and Growth Factors
Source: Front Pediatr. 2021 Sep 22;9:709765. doi: 10.3389/fped.2021.709765 (PMC8492985; doi:10.3389/fped.2021.709765)
Supplement: Supplementary file 1 [file Data_Sheet_1.zip › S4_Analysis_without_PROM_AROM.docx]

- S4.1 Loading packages and data
  - S4.1.1 CS is redefines in an understandable way and added to the other dataset
    - Renaming variables with information that are understandable instead of codes
  - S4.1.2 Removing PROM and amniotomy
  - S4.1.3 Filtering so we only have the children born on time
  - S4.1.4 Biomakers is log transformed
  - S4.1.5 Functions are defined
- S4.2 Testing the GA for all biomarkers
- S4.3 Testing birth type
- S4.4 Testing gender

S4 Code for the analysis without PROM and amniotomy during childbirth

The variables maternal age, BMI_MODER and age at sampling are all explaining some variation for some of the biomarkers, therefore all analysis of variance are made with ANCOVA (analysis of co-variance) controlling for these variables. This is done to make sure the effects seen are because of the variables tested, not one of the other variables (ruling out confounders). Birth weight are not included, since is very correlated with GA, but we expect the effects seen to be because of GA, not weight. Here the analysis is made without pre-labour rupture of the membrane (PROM) and without amniotomy during childbirth (AROM) to rule out any effects from this.

S4.1 Loading packages and data

S4.1.1 CS is redefines in an understandable way and added to the other dataset

MFR_kejsersnit <- rbind(MFR_KEJSERSNIT2009, MFR_KEJSERSNIT2010, MFR_KEJSERSNIT2011)

MFR_kejsersnit <- MFR_kejsersnit %>%

filter(SKSKODE %**in**% c("KMCA10B", "KMCA10E", "KMCA10A","KMCA10D" )) %>%

mutate(Birth_type = ifelse(SKSKODE == "KMCA10B", "Pre-labor",

ifelse(SKSKODE== "KMCA10E", "In-labor", "Slet"))) %>%

arrange(FK_MFR, Birth_type) %>%

distinct(FK_MFR, .keep_all=TRUE)

MFR2 <- MFR_kejsersnit %>%

right_join(upload_MFR, by="FK_MFR")

Renaming variables with information that are understandable instead of codes

MFR <- MFR2 %>%

mutate(tvilling = ifelse(str_detect(FLERFOLDSGRAVIDITET, "DO300"), "Ja", "Nej")) %>%

mutate(flerfold = ifelse(str_detect(FLERFOLDSGRAVIDITET, "DO30"), "Ja", "Nej")) %>%

mutate(PPROM = ifelse(PPROM=="DO422", "Ja", "Nej")) %>%

mutate(sepsis = ifelse(str_detect(SEPSIS_BARN, "DP36"), "Ja", "Nej")) %>%

replace_na(list(tvilling="Nej")) %>%

replace_na(list(flerfold="Nej")) %>%

replace_na(list(PPROM="Nej")) %>%

replace_na(list(sepsis="Nej")) %>%

replace_na(list(Birth_type="Vaginal")) %>%

mutate(CRP = CRP*1e-06) %>%

mutate(gestation_uge_f = as.factor(gestation_uge)) %>%

filter(gestation_uge > 23 & gestation_uge < 43) %>%

filter(Birth_type != "Slet") %>%

data.table

S4.1.2 Removing PROM and amniotomy

MFR <- MFR %>%

filter(is.na(PROM) & is.na(AMNITOMI_UNDER_FOEDSEL_HSP)) %>%

data.table

S4.1.3 Filtering so we only have the children born on time

MFR_3742 <- MFR %>%

filter(gestation_uge > 36 & gestation_uge < 43) %>%

data.table

MFR_3742 <- MFR_3742 %>%

mutate(Gender = ifelse(KOEN_BARN=="M", "Boys", "Girls")) %>%

data.table

rm(MFR_kejsersnit, MFR_KEJSERSNIT2009, MFR_KEJSERSNIT2010, MFR_KEJSERSNIT2011, MFR2, upload_MFR)

S4.1.4 Biomakers is log transformed

MFR_3742$logCRP <- log(MFR_3742$CRP)

MFR_3742$logIL18 <- log(MFR_3742$IL18)

MFR_3742$logMCP1 <- log(MFR_3742$MCP1)

MFR_3742$logHSP70 <- log(MFR_3742$HSP70)

MFR_3742$logSTNF_RI <- log(MFR_3742$STNF_RI)

MFR_3742$logEGF <- log(MFR_3742$EGF)

MFR_3742$logBDNF <- log(MFR_3742$BDNF)

MFR_3742$logNT3 <- log(MFR_3742$NT3)

MFR_3742$logS100B <- log(MFR_3742$S100B)

MFR_3742$logVEGF <- log(MFR_3742$VEGF)

MFR_3742$gestation_uge_f <- as.factor(MFR_3742$gestation_uge)

MFR_3740 <- MFR_3742 %>%

mutate(gest = ifelse(gestation_uge == 37, "37",

ifelse(gestation_uge == 38, "38",

ifelse(gestation_uge == 39, "39", "40+"))))

MFR_3742 %>% group_by(Gender, Birth_type, PROM, AMNITOMI_UNDER_FOEDSEL_HSP) %>% dplyr::summarize(n())

## `summarise()` regrouping output by 'Gender', 'Birth_type', 'PROM' (override with `.groups` argument)

## # A tibble: 6 x 5

## # Groups: Gender, Birth_type, PROM [6]

## Gender Birth_type PROM AMNITOMI_UNDER_FOEDSEL_HSP `n()`

## <chr> <chr> <chr> <chr> <int>

## 1 Boys In-labor <NA> <NA> 270

## 2 Boys Pre-labor <NA> <NA> 376

## 3 Boys Vaginal <NA> <NA> 2406

## 4 Girls In-labor <NA> <NA> 181

## 5 Girls Pre-labor <NA> <NA> 331

## 6 Girls Vaginal <NA> <NA> 2168

S4.1.5 Functions are defined

*# Testing the effect of the GA on the biomarkers*

bio_gest <- **function**(data, bio){

fit <- aov(log(bio) ~ AGE_AT_COLLECTION + ALDER_MODER + BMI_MODER + gestation_uge_f, data=data)

sf <- Anova(fit, type="III")

sf_lm<- summary.lm(fit)

test <- summary(pairs(emmeans(fit, ~gestation_uge_f)), adjust="holm")

**return**(list(sf, sf_lm$r.squared, test))

}

*#Testing the effect of birth type on the biomarkers*

gest_type <- **function**(data=MFR_3740, bio){

MFR_3740_2 <- data %>%

filter(Birth_type != "Slet") %>%

data.table

m_under <- MFR_3740_2 %>%

filter(Birth_type != "In-labor") %>%

data.table

m_planlagt <- MFR_3740_2 %>%

filter(Birth_type != "Pre-labor") %>%

data.table

m_vaginalt <- MFR_3740_2 %>%

filter(Birth_type != "Vaginal") %>%

data.table

vag_in <- Anova(aov(m_planlagt[[bio]] ~ AGE_AT_COLLECTION + ALDER_MODER + BMI_MODER + gest + Birth_type, data=m_planlagt), type="III")

vag_pre <- Anova(aov(m_under[[bio]] ~ AGE_AT_COLLECTION + ALDER_MODER + BMI_MODER + gest + Birth_type, data=m_under), type="III")

in_pre <- Anova(aov(m_vaginalt[[bio]] ~ AGE_AT_COLLECTION + ALDER_MODER + BMI_MODER + gest + Birth_type, data=m_vaginalt), type="III")

fit <- aov(MFR_3740_2[[bio]] ~ AGE_AT_COLLECTION + ALDER_MODER + BMI_MODER + Birth_type + gest + Birth_type:gest, data=MFR_3740_2)

test <- summary(pairs(emmeans(fit, ~ Birth_type|gest)),by=NULL, adjust="holm")

**return**(list("Vaginal-inlabor"=vag_in, "Vaginal-prelabor"=vag_pre,"Inlabor-prelabor"=in_pre,"Pairwise test"=test))

}

*#Testing the effect of gender on the biomarkers*

koen_type <- **function**(data=MFR_3740, bio){

MFR_3740_2 <- data %>%

filter(Birth_type != "Slet") %>%

data.table

boys <- MFR_3740_2 %>%

filter(Gender == "Boys") %>%

data.table

girls <- MFR_3740_2 %>%

filter(Gender == "Girls") %>%

data.table

b<-Anova(aov(boys[[bio]] ~ AGE_AT_COLLECTION + ALDER_MODER + BMI_MODER + Birth_type, data=boys), type="III")

g<-Anova(aov(girls[[bio]] ~ AGE_AT_COLLECTION + ALDER_MODER + BMI_MODER + Birth_type, data=girls), type="III")

fit <- aov(MFR_3740_2[[bio]] ~ AGE_AT_COLLECTION + ALDER_MODER + BMI_MODER + Birth_type + KOEN_BARN + Birth_type:KOEN_BARN, data=MFR_3740_2)

test <- summary(pairs(emmeans(fit, ~ KOEN_BARN|Birth_type)), by=NULL, adjust="holm")

**return**(list("Boys"=b, "Girls"=g,"Pairwise test"=test))

}

S4.2 Testing the GA for all biomarkers

Figure 3 in the paper.

bio_gest(data=MFR_3742, bio=MFR_3742$CRP)

## [[1]]

## Anova Table (Type III tests)

##

## Response: log(bio)

## Sum Sq Df F value Pr(>F)

## (Intercept) 66.16 1 121.9798 < 2.2e-16 ***

## AGE_AT_COLLECTION 10.41 1 19.2001 1.199e-05 ***

## ALDER_MODER 7.40 1 13.6396 0.0002236 ***

## BMI_MODER 0.02 1 0.0388 0.8438918

## gestation_uge_f 179.48 5 66.1757 < 2.2e-16 ***

## Residuals 2975.73 5486

## ---

## Signif. codes: 0 '***' 0.001 '**' 0.01 '*' 0.05 '.' 0.1 ' ' 1

##

## [[2]]

## [1] 0.06239423

##

## [[3]]

## contrast estimate SE df t.ratio p.value

## 37 - 38 -0.1080 0.0475 5486 -2.275 0.0688

## 37 - 39 -0.2907 0.0453 5486 -6.414 <.0001

## 37 - 40 -0.4921 0.0444 5486 -11.087 <.0001

## 37 - 41 -0.5213 0.0463 5486 -11.271 <.0001

## 37 - 42 -0.6038 0.0574 5486 -10.526 <.0001

## 38 - 39 -0.1828 0.0323 5486 -5.658 <.0001

## 38 - 40 -0.3841 0.0310 5486 -12.389 <.0001

## 38 - 41 -0.4134 0.0336 5486 -12.287 <.0001

## 38 - 42 -0.4958 0.0478 5486 -10.368 <.0001

## 39 - 40 -0.2014 0.0275 5486 -7.311 <.0001

## 39 - 41 -0.2306 0.0305 5486 -7.563 <.0001

## 39 - 42 -0.3130 0.0457 5486 -6.852 <.0001

## 40 - 41 -0.0292 0.0291 5486 -1.004 0.3154

## 40 - 42 -0.1117 0.0447 5486 -2.496 0.0504

## 41 - 42 -0.0825 0.0466 5486 -1.770 0.1537

##

## Results are given on the log (not the response) scale.

## P value adjustment: holm method for 15 tests

bio_gest(data=MFR_3742, bio=MFR_3742$MCP1)

## [[1]]

## Anova Table (Type III tests)

##

## Response: log(bio)

## Sum Sq Df F value Pr(>F)

## (Intercept) 1877.31 1 10194.9853 < 2e-16 ***

## AGE_AT_COLLECTION 36.02 1 195.6209 < 2e-16 ***

## ALDER_MODER 0.86 1 4.6527 0.03105 *

## BMI_MODER 0.26 1 1.4273 0.23226

## gestation_uge_f 22.94 5 24.9150 < 2e-16 ***

## Residuals 1010.19 5486

## ---

## Signif. codes: 0 '***' 0.001 '**' 0.01 '*' 0.05 '.' 0.1 ' ' 1

##

## [[2]]

## [1] 0.0550196

##

## [[3]]

## contrast estimate SE df t.ratio p.value

## 37 - 38 0.0283 0.0277 5486 1.022 0.8938

## 37 - 39 -0.0435 0.0264 5486 -1.646 0.4995

## 37 - 40 -0.1225 0.0259 5486 -4.736 <.0001

## 37 - 41 -0.1401 0.0269 5486 -5.200 <.0001

## 37 - 42 -0.1614 0.0334 5486 -4.828 <.0001

## 38 - 39 -0.0717 0.0188 5486 -3.811 0.0008

## 38 - 40 -0.1507 0.0181 5486 -8.344 <.0001

## 38 - 41 -0.1684 0.0196 5486 -8.591 <.0001

## 38 - 42 -0.1896 0.0279 5486 -6.805 <.0001

## 39 - 40 -0.0790 0.0160 5486 -4.924 <.0001

## 39 - 41 -0.0967 0.0178 5486 -5.442 <.0001

## 39 - 42 -0.1179 0.0266 5486 -4.428 0.0001

## 40 - 41 -0.0177 0.0170 5486 -1.041 0.8938

## 40 - 42 -0.0389 0.0261 5486 -1.491 0.5441

## 41 - 42 -0.0212 0.0271 5486 -0.781 0.8938

##

## Results are given on the log (not the response) scale.

## P value adjustment: holm method for 15 tests

bio_gest(data=MFR_3742, bio=MFR_3742$IL18)

## [[1]]

## Anova Table (Type III tests)

##

## Response: log(bio)

## Sum Sq Df F value Pr(>F)

## (Intercept) 532.72 1 2979.3580 < 2.2e-16 ***

## AGE_AT_COLLECTION 0.03 1 0.1890 0.66373

## ALDER_MODER 0.07 1 0.3931 0.53070

## BMI_MODER 0.69 1 3.8801 0.04891 *

## gestation_uge_f 5.67 5 6.3368 7.124e-06 ***

## Residuals 980.92 5486

## ---

## Signif. codes: 0 '***' 0.001 '**' 0.01 '*' 0.05 '.' 0.1 ' ' 1

##

## [[2]]

## [1] 0.006742082

##

## [[3]]

## contrast estimate SE df t.ratio p.value

## 37 - 38 -0.02185 0.0273 5486 -0.802 1.0000

## 37 - 39 -0.01716 0.0260 5486 -0.659 1.0000

## 37 - 40 -0.06065 0.0255 5486 -2.380 0.1215

## 37 - 41 -0.07180 0.0266 5486 -2.704 0.0657

## 37 - 42 -0.13062 0.0329 5486 -3.966 0.0010

## 38 - 39 0.00469 0.0185 5486 0.253 1.0000

## 38 - 40 -0.03880 0.0178 5486 -2.179 0.1677

## 38 - 41 -0.04995 0.0193 5486 -2.586 0.0779

## 38 - 42 -0.10877 0.0275 5486 -3.962 0.0010

## 39 - 40 -0.04349 0.0158 5486 -2.750 0.0657

## 39 - 41 -0.05464 0.0175 5486 -3.121 0.0217

## 39 - 42 -0.11346 0.0262 5486 -4.325 0.0002

## 40 - 41 -0.01115 0.0167 5486 -0.667 1.0000

## 40 - 42 -0.06997 0.0257 5486 -2.724 0.0657

## 41 - 42 -0.05882 0.0268 5486 -2.199 0.1677

##

## Results are given on the log (not the response) scale.

## P value adjustment: holm method for 15 tests

bio_gest(data=MFR_3742, bio=MFR_3742$HSP70)

## [[1]]

## Anova Table (Type III tests)

##

## Response: log(bio)

## Sum Sq Df F value Pr(>F)

## (Intercept) 8146.8 1 93951.6679 < 2e-16 ***

## AGE_AT_COLLECTION 0.0 1 0.0285 0.86601

## ALDER_MODER 0.0 1 0.3095 0.57798

## BMI_MODER 0.3 1 3.7471 0.05295 .

## gestation_uge_f 1.0 5 2.2100 0.05056 .

## Residuals 475.7 5486

## ---

## Signif. codes: 0 '***' 0.001 '**' 0.01 '*' 0.05 '.' 0.1 ' ' 1

##

## [[2]]

## [1] 0.002813677

##

## [[3]]

## contrast estimate SE df t.ratio p.value

## 37 - 38 0.00450 0.0190 5486 0.237 1.0000

## 37 - 39 0.03747 0.0181 5486 2.067 0.5037

## 37 - 40 0.03335 0.0177 5486 1.879 0.7234

## 37 - 41 0.02296 0.0185 5486 1.241 1.0000

## 37 - 42 0.01011 0.0229 5486 0.441 1.0000

## 38 - 39 0.03297 0.0129 5486 2.553 0.1606

## 38 - 40 0.02885 0.0124 5486 2.327 0.2799

## 38 - 41 0.01846 0.0135 5486 1.372 1.0000

## 38 - 42 0.00561 0.0191 5486 0.293 1.0000

## 39 - 40 -0.00412 0.0110 5486 -0.374 1.0000

## 39 - 41 -0.01451 0.0122 5486 -1.190 1.0000

## 39 - 42 -0.02736 0.0183 5486 -1.498 1.0000

## 40 - 41 -0.01039 0.0116 5486 -0.893 1.0000

## 40 - 42 -0.02324 0.0179 5486 -1.299 1.0000

## 41 - 42 -0.01285 0.0186 5486 -0.690 1.0000

##

## Results are given on the log (not the response) scale.

## P value adjustment: holm method for 15 tests

bio_gest(data=MFR_3742, bio=MFR_3742$STNF_RI)

## [[1]]

## Anova Table (Type III tests)

##

## Response: log(bio)

## Sum Sq Df F value Pr(>F)

## (Intercept) 1420.74 1 4980.1963 < 2.2e-16 ***

## AGE_AT_COLLECTION 33.40 1 117.0612 < 2.2e-16 ***

## ALDER_MODER 3.29 1 11.5382 0.0006867 ***

## BMI_MODER 3.61 1 12.6629 0.0003761 ***

## gestation_uge_f 3.99 5 2.7947 0.0158699 *

## Residuals 1565.04 5486

## ---

## Signif. codes: 0 '***' 0.001 '**' 0.01 '*' 0.05 '.' 0.1 ' ' 1

##

## [[2]]

## [1] 0.02706895

##

## [[3]]

## contrast estimate SE df t.ratio p.value

## 37 - 38 -0.00809 0.0344 5486 -0.235 1.0000

## 37 - 39 0.06181 0.0329 5486 1.880 0.6532

## 37 - 40 0.04320 0.0322 5486 1.342 1.0000

## 37 - 41 0.06325 0.0335 5486 1.886 0.6532

## 37 - 42 0.06827 0.0416 5486 1.641 0.9072

## 38 - 39 0.06990 0.0234 5486 2.984 0.0428

## 38 - 40 0.05129 0.0225 5486 2.281 0.2935

## 38 - 41 0.07135 0.0244 5486 2.924 0.0485

## 38 - 42 0.07637 0.0347 5486 2.202 0.3324

## 39 - 40 -0.01861 0.0200 5486 -0.932 1.0000

## 39 - 41 0.00144 0.0221 5486 0.065 1.0000

## 39 - 42 0.00646 0.0331 5486 0.195 1.0000

## 40 - 41 0.02006 0.0211 5486 0.950 1.0000

## 40 - 42 0.02507 0.0325 5486 0.773 1.0000

## 41 - 42 0.00502 0.0338 5486 0.149 1.0000

##

## Results are given on the log (not the response) scale.

## P value adjustment: holm method for 15 tests

bio_gest(data=MFR_3742, bio=MFR_3742$EGF)

## [[1]]

## Anova Table (Type III tests)

##

## Response: log(bio)

## Sum Sq Df F value Pr(>F)

## (Intercept) 543.69 1 2591.8182 < 2.2e-16 ***

## AGE_AT_COLLECTION 2.36 1 11.2517 0.0008009 ***

## ALDER_MODER 0.78 1 3.7105 0.0541243 .

## BMI_MODER 0.87 1 4.1463 0.0417723 *

## gestation_uge_f 2.67 5 2.5488 0.0260186 *

## Residuals 1150.81 5486

## ---

## Signif. codes: 0 '***' 0.001 '**' 0.01 '*' 0.05 '.' 0.1 ' ' 1

##

## [[2]]

## [1] 0.005785295

##

## [[3]]

## contrast estimate SE df t.ratio p.value

## 37 - 38 -0.00735 0.0295 5486 -0.249 1.0000

## 37 - 39 0.03319 0.0282 5486 1.177 1.0000

## 37 - 40 0.01450 0.0276 5486 0.525 1.0000

## 37 - 41 0.04135 0.0288 5486 1.437 1.0000

## 37 - 42 0.08078 0.0357 5486 2.265 0.2829

## 38 - 39 0.04053 0.0201 5486 2.018 0.4800

## 38 - 40 0.02184 0.0193 5486 1.133 1.0000

## 38 - 41 0.04869 0.0209 5486 2.327 0.2598

## 38 - 42 0.08813 0.0297 5486 2.964 0.0458

## 39 - 40 -0.01869 0.0171 5486 -1.091 1.0000

## 39 - 41 0.00816 0.0190 5486 0.430 1.0000

## 39 - 42 0.04760 0.0284 5486 1.675 0.9397

## 40 - 41 0.02685 0.0181 5486 1.483 1.0000

## 40 - 42 0.06629 0.0278 5486 2.382 0.2414

## 41 - 42 0.03944 0.0290 5486 1.361 1.0000

##

## Results are given on the log (not the response) scale.

## P value adjustment: holm method for 15 tests

bio_gest(data=MFR_3742, bio=MFR_3742$VEGF)

## [[1]]

## Anova Table (Type III tests)

##

## Response: log(bio)

## Sum Sq Df F value Pr(>F)

## (Intercept) 942.50 1 4891.8764 < 2.2e-16 ***

## AGE_AT_COLLECTION 42.70 1 221.6015 < 2.2e-16 ***

## ALDER_MODER 0.39 1 2.0200 0.155292

## BMI_MODER 0.73 1 3.8133 0.050898 .

## gestation_uge_f 3.23 5 3.3540 0.005001 **

## Residuals 1056.97 5486

## ---

## Signif. codes: 0 '***' 0.001 '**' 0.01 '*' 0.05 '.' 0.1 ' ' 1

##

## [[2]]

## [1] 0.04278488

##

## [[3]]

## contrast estimate SE df t.ratio p.value

## 37 - 38 -0.00627 0.0283 5486 -0.222 1.0000

## 37 - 39 0.03994 0.0270 5486 1.479 1.0000

## 37 - 40 0.04578 0.0265 5486 1.731 0.7519

## 37 - 41 0.05946 0.0276 5486 2.157 0.3105

## 37 - 42 0.07523 0.0342 5486 2.201 0.3058

## 38 - 39 0.04621 0.0192 5486 2.401 0.1968

## 38 - 40 0.05205 0.0185 5486 2.817 0.0632

## 38 - 41 0.06573 0.0201 5486 3.278 0.0158

## 38 - 42 0.08150 0.0285 5486 2.860 0.0596

## 39 - 40 0.00584 0.0164 5486 0.356 1.0000

## 39 - 41 0.01952 0.0182 5486 1.074 1.0000

## 39 - 42 0.03529 0.0272 5486 1.296 1.0000

## 40 - 41 0.01368 0.0174 5486 0.788 1.0000

## 40 - 42 0.02945 0.0267 5486 1.104 1.0000

## 41 - 42 0.01577 0.0278 5486 0.568 1.0000

##

## Results are given on the log (not the response) scale.

## P value adjustment: holm method for 15 tests

bio_gest(data=MFR_3742, bio=MFR_3742$S100B)

## [[1]]

## Anova Table (Type III tests)

##

## Response: log(bio)

## Sum Sq Df F value Pr(>F)

## (Intercept) 1803.95 1 5914.6497 < 2e-16 ***

## AGE_AT_COLLECTION 0.64 1 2.0840 0.14891

## ALDER_MODER 0.08 1 0.2600 0.61016

## BMI_MODER 0.02 1 0.0718 0.78879

## gestation_uge_f 4.10 5 2.6856 0.01978 *

## Residuals 1673.21 5486

## ---

## Signif. codes: 0 '***' 0.001 '**' 0.01 '*' 0.05 '.' 0.1 ' ' 1

##

## [[2]]

## [1] 0.002824222

##

## [[3]]

## contrast estimate SE df t.ratio p.value

## 37 - 38 0.0154 0.0356 5486 0.434 1.0000

## 37 - 39 0.0387 0.0340 5486 1.137 1.0000

## 37 - 40 0.0591 0.0333 5486 1.777 0.6553

## 37 - 41 0.0807 0.0347 5486 2.326 0.2407

## 37 - 42 0.1032 0.0430 5486 2.400 0.2137

## 38 - 39 0.0232 0.0242 5486 0.959 1.0000

## 38 - 40 0.0437 0.0232 5486 1.880 0.6553

## 38 - 41 0.0652 0.0252 5486 2.586 0.1460

## 38 - 42 0.0878 0.0359 5486 2.448 0.2014

## 39 - 40 0.0205 0.0207 5486 0.992 1.0000

## 39 - 41 0.0420 0.0229 5486 1.837 0.6553

## 39 - 42 0.0646 0.0343 5486 1.884 0.6553

## 40 - 41 0.0215 0.0218 5486 0.986 1.0000

## 40 - 42 0.0441 0.0336 5486 1.314 1.0000

## 41 - 42 0.0225 0.0349 5486 0.645 1.0000

##

## Results are given on the log (not the response) scale.

## P value adjustment: holm method for 15 tests

bio_gest(data=MFR_3742, bio=MFR_3742$BDNF)

## [[1]]

## Anova Table (Type III tests)

##

## Response: log(bio)

## Sum Sq Df F value Pr(>F)

## (Intercept) 2114.95 1 3881.7585 < 2.2e-16 ***

## AGE_AT_COLLECTION 4.03 1 7.4032 0.0065315 **

## ALDER_MODER 7.07 1 12.9832 0.0003171 ***

## BMI_MODER 6.34 1 11.6337 0.0006523 ***

## gestation_uge_f 5.38 5 1.9755 0.0789598 .

## Residuals 2989.01 5486

## ---

## Signif. codes: 0 '***' 0.001 '**' 0.01 '*' 0.05 '.' 0.1 ' ' 1

##

## [[2]]

## [1] 0.007756866

##

## [[3]]

## contrast estimate SE df t.ratio p.value

## 37 - 38 -0.0709 0.0476 5486 -1.490 1.0000

## 37 - 39 -0.0490 0.0454 5486 -1.079 1.0000

## 37 - 40 -0.0810 0.0445 5486 -1.820 0.7572

## 37 - 41 -0.0592 0.0464 5486 -1.277 1.0000

## 37 - 42 0.0412 0.0575 5486 0.716 1.0000

## 38 - 39 0.0219 0.0324 5486 0.675 1.0000

## 38 - 40 -0.0101 0.0311 5486 -0.325 1.0000

## 38 - 41 0.0117 0.0337 5486 0.346 1.0000

## 38 - 42 0.1120 0.0479 5486 2.337 0.2723

## 39 - 40 -0.0320 0.0276 5486 -1.158 1.0000

## 39 - 41 -0.0102 0.0306 5486 -0.334 1.0000

## 39 - 42 0.0902 0.0458 5486 1.969 0.5881

## 40 - 41 0.0217 0.0292 5486 0.745 1.0000

## 40 - 42 0.1221 0.0448 5486 2.723 0.0973

## 41 - 42 0.1004 0.0467 5486 2.149 0.4114

##

## Results are given on the log (not the response) scale.

## P value adjustment: holm method for 15 tests

bio_gest(data=MFR_3742, bio=MFR_3742$NT3)

## [[1]]

## Anova Table (Type III tests)

##

## Response: log(bio)

## Sum Sq Df F value Pr(>F)

## (Intercept) 115.80 1 486.9786 < 2.2e-16 ***

## AGE_AT_COLLECTION 3.29 1 13.8401 0.000201 ***

## ALDER_MODER 1.50 1 6.3201 0.011966 *

## BMI_MODER 0.86 1 3.6367 0.056570 .

## gestation_uge_f 1.33 5 1.1155 0.349640

## Residuals 1304.57 5486

## ---

## Signif. codes: 0 '***' 0.001 '**' 0.01 '*' 0.05 '.' 0.1 ' ' 1

##

## [[2]]

## [1] 0.005280245

##

## [[3]]

## contrast estimate SE df t.ratio p.value

## 37 - 38 -0.04987 0.0314 5486 -1.587 1.0000

## 37 - 39 -0.02596 0.0300 5486 -0.865 1.0000

## 37 - 40 -0.05569 0.0294 5486 -1.895 0.8722

## 37 - 41 -0.04590 0.0306 5486 -1.499 1.0000

## 37 - 42 -0.03083 0.0380 5486 -0.812 1.0000

## 38 - 39 0.02391 0.0214 5486 1.118 1.0000

## 38 - 40 -0.00582 0.0205 5486 -0.284 1.0000

## 38 - 41 0.00396 0.0223 5486 0.178 1.0000

## 38 - 42 0.01904 0.0317 5486 0.601 1.0000

## 39 - 40 -0.02973 0.0182 5486 -1.630 1.0000

## 39 - 41 -0.01994 0.0202 5486 -0.988 1.0000

## 39 - 42 -0.00487 0.0303 5486 -0.161 1.0000

## 40 - 41 0.00979 0.0193 5486 0.508 1.0000

## 40 - 42 0.02486 0.0296 5486 0.839 1.0000

## 41 - 42 0.01507 0.0309 5486 0.489 1.0000

##

## Results are given on the log (not the response) scale.

## P value adjustment: holm method for 15 tests

S4.3 Testing birth type

Figure 1 i paper

cleaning up the data and splitting it up

Testing Here an ancova is made for each biomaker for each of the pairs of birthtypes. This is further corrected for GA

gest_type(bio="logCRP")

## $`Vaginal-inlabor`

## Anova Table (Type III tests)

##

## Response: m_planlagt[[bio]]

## Sum Sq Df F value Pr(>F)

## (Intercept) 55.37 1 106.7316 < 2.2e-16 ***

## AGE_AT_COLLECTION 16.58 1 31.9560 1.668e-08 ***

## ALDER_MODER 2.97 1 5.7192 0.016818 *

## BMI_MODER 0.52 1 1.0020 0.316887

## gest 75.33 3 48.3953 < 2.2e-16 ***

## Birth_type 5.10 1 9.8281 0.001729 **

## Residuals 2492.93 4805

## ---

## Signif. codes: 0 '***' 0.001 '**' 0.01 '*' 0.05 '.' 0.1 ' ' 1

##

## $`Vaginal-prelabor`

## Anova Table (Type III tests)

##

## Response: m_under[[bio]]

## Sum Sq Df F value Pr(>F)

## (Intercept) 90.91 1 173.2832 < 2.2e-16 ***

## AGE_AT_COLLECTION 18.31 1 34.9087 3.682e-09 ***

## ALDER_MODER 2.40 1 4.5759 0.03247 *

## BMI_MODER 0.56 1 1.0767 0.29949

## gest 70.02 3 44.4901 < 2.2e-16 ***

## Birth_type 70.60 1 134.5703 < 2.2e-16 ***

## Residuals 2649.31 5050

## ---

## Signif. codes: 0 '***' 0.001 '**' 0.01 '*' 0.05 '.' 0.1 ' ' 1

##

## $`Inlabor-prelabor`

## Anova Table (Type III tests)

##

## Response: m_vaginalt[[bio]]

## Sum Sq Df F value Pr(>F)

## (Intercept) 22.93 1 38.0881 9.461e-10 ***

## AGE_AT_COLLECTION 0.43 1 0.7147 0.3981

## ALDER_MODER 0.03 1 0.0509 0.8216

## BMI_MODER 0.36 1 0.6037 0.4373

## gest 17.46 3 9.6705 2.650e-06 ***

## Birth_type 10.39 1 17.2593 3.511e-05 ***

## Residuals 668.79 1111

## ---

## Signif. codes: 0 '***' 0.001 '**' 0.01 '*' 0.05 '.' 0.1 ' ' 1

##

## $`Pairwise test`

## contrast gest estimate SE df t.ratio p.value

## (In-labor) - (Pre-labor) 37 0.0352 0.1505 5480 0.234 0.9644

## (In-labor) - Vaginal 37 -0.3024 0.1403 5480 -2.155 0.1560

## (Pre-labor) - Vaginal 37 -0.3375 0.0903 5480 -3.739 0.0019

## (In-labor) - (Pre-labor) 38 0.2902 0.1012 5480 2.868 0.0290

## (In-labor) - Vaginal 38 -0.1141 0.0973 5480 -1.173 0.7222

## (Pre-labor) - Vaginal 38 -0.4043 0.0535 5480 -7.556 <.0001

## (In-labor) - (Pre-labor) 39 0.3274 0.0924 5480 3.544 0.0036

## (In-labor) - Vaginal 39 -0.0586 0.0833 5480 -0.703 0.9644

## (Pre-labor) - Vaginal 39 -0.3859 0.0519 5480 -7.430 <.0001

## (In-labor) - (Pre-labor) 40+ 0.2634 0.1267 5480 2.079 0.1560

## (In-labor) - Vaginal 40+ -0.1100 0.0473 5480 -2.326 0.1203

## (Pre-labor) - Vaginal 40+ -0.3734 0.1192 5480 -3.133 0.0139

##

## P value adjustment: holm method for 12 tests

gest_type(bio="logMCP1")

## $`Vaginal-inlabor`

## Anova Table (Type III tests)

##

## Response: m_planlagt[[bio]]

## Sum Sq Df F value Pr(>F)

## (Intercept) 1470.23 1 7922.1123 < 2.2e-16 ***

## AGE_AT_COLLECTION 40.19 1 216.5323 < 2.2e-16 ***

## ALDER_MODER 0.37 1 1.9671 0.1608

## BMI_MODER 0.04 1 0.2331 0.6293

## gest 7.73 3 13.8816 5.199e-09 ***

## Birth_type 0.15 1 0.8006 0.3709

## Residuals 891.74 4805

## ---

## Signif. codes: 0 '***' 0.001 '**' 0.01 '*' 0.05 '.' 0.1 ' ' 1

##

## $`Vaginal-prelabor`

## Anova Table (Type III tests)

##

## Response: m_under[[bio]]

## Sum Sq Df F value Pr(>F)

## (Intercept) 1523.59 1 8433.7296 < 2.2e-16 ***

## AGE_AT_COLLECTION 38.94 1 215.5699 < 2.2e-16 ***

## ALDER_MODER 0.18 1 1.0228 0.3119

## BMI_MODER 0.08 1 0.4674 0.4942

## gest 8.18 3 15.0865 8.975e-10 ***

## Birth_type 12.86 1 71.1648 < 2.2e-16 ***

## Residuals 912.30 5050

## ---

## Signif. codes: 0 '***' 0.001 '**' 0.01 '*' 0.05 '.' 0.1 ' ' 1

##

## $`Inlabor-prelabor`

## Anova Table (Type III tests)

##

## Response: m_vaginalt[[bio]]

## Sum Sq Df F value Pr(>F)

## (Intercept) 321.90 1 1889.4936 < 2.2e-16 ***

## AGE_AT_COLLECTION 3.17 1 18.6084 1.748e-05 ***

## ALDER_MODER 0.06 1 0.3449 0.5571

## BMI_MODER 0.00 1 0.0004 0.9843

## gest 0.96 3 1.8869 0.1300

## Birth_type 3.66 1 21.4627 4.034e-06 ***

## Residuals 189.27 1111

## ---

## Signif. codes: 0 '***' 0.001 '**' 0.01 '*' 0.05 '.' 0.1 ' ' 1

##

## $`Pairwise test`

## contrast gest estimate SE df t.ratio p.value

## (In-labor) - (Pre-labor) 37 0.1168 0.0882 5480 1.325 1.0000

## (In-labor) - Vaginal 37 -0.0444 0.0822 5480 -0.540 1.0000

## (Pre-labor) - Vaginal 37 -0.1613 0.0529 5480 -3.049 0.0208

## (In-labor) - (Pre-labor) 38 0.1628 0.0593 5480 2.748 0.0482

## (In-labor) - Vaginal 38 0.0028 0.0570 5480 0.049 1.0000

## (Pre-labor) - Vaginal 38 -0.1600 0.0313 5480 -5.106 <.0001

## (In-labor) - (Pre-labor) 39 0.2051 0.0541 5480 3.790 0.0015

## (In-labor) - Vaginal 39 0.0302 0.0488 5480 0.619 1.0000

## (Pre-labor) - Vaginal 39 -0.1749 0.0304 5480 -5.746 <.0001

## (In-labor) - (Pre-labor) 40+ 0.0999 0.0742 5480 1.346 1.0000

## (In-labor) - Vaginal 40+ -0.0380 0.0277 5480 -1.372 1.0000

## (Pre-labor) - Vaginal 40+ -0.1379 0.0698 5480 -1.975 0.3379

##

## P value adjustment: holm method for 12 tests

gest_type(bio="logIL18")

## $`Vaginal-inlabor`

## Anova Table (Type III tests)

##

## Response: m_planlagt[[bio]]

## Sum Sq Df F value Pr(>F)

## (Intercept) 417.86 1 2338.7666 < 2.2e-16 ***

## AGE_AT_COLLECTION 0.06 1 0.3504 0.553904

## ALDER_MODER 0.05 1 0.3025 0.582356

## BMI_MODER 0.90 1 5.0357 0.024876 *

## gest 2.48 3 4.6303 0.003084 **

## Birth_type 0.00 1 0.0001 0.993387

## Residuals 858.50 4805

## ---

## Signif. codes: 0 '***' 0.001 '**' 0.01 '*' 0.05 '.' 0.1 ' ' 1

##

## $`Vaginal-prelabor`

## Anova Table (Type III tests)

##

## Response: m_under[[bio]]

## Sum Sq Df F value Pr(>F)

## (Intercept) 431.72 1 2444.0400 < 2.2e-16 ***

## AGE_AT_COLLECTION 0.00 1 0.0147 0.903662

## ALDER_MODER 0.33 1 1.8843 0.169905

## BMI_MODER 0.84 1 4.7528 0.029296 *

## gest 2.32 3 4.3756 0.004403 **

## Birth_type 1.25 1 7.0721 0.007854 **

## Residuals 892.04 5050

## ---

## Signif. codes: 0 '***' 0.001 '**' 0.01 '*' 0.05 '.' 0.1 ' ' 1

##

## $`Inlabor-prelabor`

## Anova Table (Type III tests)

##

## Response: m_vaginalt[[bio]]

## Sum Sq Df F value Pr(>F)

## (Intercept) 95.890 1 506.3944 < 2e-16 ***

## AGE_AT_COLLECTION 0.692 1 3.6561 0.05612 .

## ALDER_MODER 0.013 1 0.0688 0.79318

## BMI_MODER 0.086 1 0.4534 0.50084

## gest 0.425 3 0.7489 0.52305

## Birth_type 0.213 1 1.1236 0.28938

## Residuals 210.377 1111

## ---

## Signif. codes: 0 '***' 0.001 '**' 0.01 '*' 0.05 '.' 0.1 ' ' 1

##

## $`Pairwise test`

## contrast gest estimate SE df t.ratio p.value

## (In-labor) - (Pre-labor) 37 0.086324 0.0874 5480 0.987 1.0000

## (In-labor) - Vaginal 37 0.099410 0.0815 5480 1.220 1.0000

## (Pre-labor) - Vaginal 37 0.013085 0.0525 5480 0.249 1.0000

## (In-labor) - (Pre-labor) 38 -0.006261 0.0588 5480 -0.107 1.0000

## (In-labor) - Vaginal 38 -0.058195 0.0565 5480 -1.030 1.0000

## (Pre-labor) - Vaginal 38 -0.051934 0.0311 5480 -1.671 1.0000

## (In-labor) - (Pre-labor) 39 0.071676 0.0537 5480 1.336 1.0000

## (In-labor) - Vaginal 39 0.005217 0.0484 5480 0.108 1.0000

## (Pre-labor) - Vaginal 39 -0.066458 0.0302 5480 -2.202 0.3322

## (In-labor) - (Pre-labor) 40+ 0.065074 0.0736 5480 0.884 1.0000

## (In-labor) - Vaginal 40+ 0.000276 0.0275 5480 0.010 1.0000

## (Pre-labor) - Vaginal 40+ -0.064798 0.0692 5480 -0.936 1.0000

##

## P value adjustment: holm method for 12 tests

gest_type(bio="logHSP70")

## $`Vaginal-inlabor`

## Anova Table (Type III tests)

##

## Response: m_planlagt[[bio]]

## Sum Sq Df F value Pr(>F)

## (Intercept) 6333.4 1 74048.1026 < 2.2e-16 ***

## AGE_AT_COLLECTION 0.0 1 0.4963 0.4812

## ALDER_MODER 0.0 1 0.0074 0.9314

## BMI_MODER 0.4 1 4.6834 0.0305 *

## gest 1.9 3 7.3023 6.978e-05 ***

## Birth_type 0.2 1 2.7536 0.0971 .

## Residuals 411.0 4805

## ---

## Signif. codes: 0 '***' 0.001 '**' 0.01 '*' 0.05 '.' 0.1 ' ' 1

##

## $`Vaginal-prelabor`

## Anova Table (Type III tests)

##

## Response: m_under[[bio]]

## Sum Sq Df F value Pr(>F)

## (Intercept) 6836.7 1 79605.6328 < 2.2e-16 ***

## AGE_AT_COLLECTION 0.0 1 0.4675 0.49416

## ALDER_MODER 0.0 1 0.2788 0.59752

## BMI_MODER 0.4 1 4.1929 0.04064 *

## gest 2.4 3 9.2127 4.481e-06 ***

## Birth_type 3.9 1 45.5533 1.652e-11 ***

## Residuals 433.7 5050

## ---

## Signif. codes: 0 '***' 0.001 '**' 0.01 '*' 0.05 '.' 0.1 ' ' 1

##

## $`Inlabor-prelabor`

## Anova Table (Type III tests)

##

## Response: m_vaginalt[[bio]]

## Sum Sq Df F value Pr(>F)

## (Intercept) 1507.26 1 17053.7504 < 2.2e-16 ***

## AGE_AT_COLLECTION 0.00 1 0.0298 0.8630800

## ALDER_MODER 0.03 1 0.3532 0.5524540

## BMI_MODER 0.23 1 2.6129 0.1062844

## gest 0.18 3 0.6837 0.5620564

## Birth_type 1.34 1 15.1320 0.0001062 ***

## Residuals 98.19 1111

## ---

## Signif. codes: 0 '***' 0.001 '**' 0.01 '*' 0.05 '.' 0.1 ' ' 1

##

## $`Pairwise test`

## contrast gest estimate SE df t.ratio p.value

## (In-labor) - (Pre-labor) 37 0.00949 0.0606 5480 0.157 1.0000

## (In-labor) - Vaginal 37 -0.05595 0.0565 5480 -0.990 1.0000

## (Pre-labor) - Vaginal 37 -0.06544 0.0364 5480 -1.800 0.5035

## (In-labor) - (Pre-labor) 38 0.08826 0.0407 5480 2.166 0.2729

## (In-labor) - Vaginal 38 -0.02325 0.0392 5480 -0.594 1.0000

## (Pre-labor) - Vaginal 38 -0.11151 0.0215 5480 -5.175 <.0001

## (In-labor) - (Pre-labor) 39 0.12527 0.0372 5480 3.368 0.0076

## (In-labor) - Vaginal 39 0.03734 0.0336 5480 1.112 1.0000

## (Pre-labor) - Vaginal 39 -0.08793 0.0209 5480 -4.204 0.0003

## (In-labor) - (Pre-labor) 40+ 0.08778 0.0510 5480 1.721 0.5121

## (In-labor) - Vaginal 40+ 0.04049 0.0191 5480 2.125 0.2729

## (Pre-labor) - Vaginal 40+ -0.04730 0.0480 5480 -0.985 1.0000

##

## P value adjustment: holm method for 12 tests

gest_type(bio="logSTNF_RI")

## $`Vaginal-inlabor`

## Anova Table (Type III tests)

##

## Response: m_planlagt[[bio]]

## Sum Sq Df F value Pr(>F)

## (Intercept) 1126.86 1 3920.2444 < 2.2e-16 ***

## AGE_AT_COLLECTION 30.87 1 107.4031 < 2.2e-16 ***

## ALDER_MODER 3.09 1 10.7638 0.001042 **

## BMI_MODER 4.72 1 16.4064 5.192e-05 ***

## gest 2.23 3 2.5879 0.051280 .

## Birth_type 0.86 1 3.0010 0.083279 .

## Residuals 1381.17 4805

## ---

## Signif. codes: 0 '***' 0.001 '**' 0.01 '*' 0.05 '.' 0.1 ' ' 1

##

## $`Vaginal-prelabor`

## Anova Table (Type III tests)

##

## Response: m_under[[bio]]

## Sum Sq Df F value Pr(>F)

## (Intercept) 1204.81 1 4237.4916 < 2.2e-16 ***

## AGE_AT_COLLECTION 33.19 1 116.7329 < 2.2e-16 ***

## ALDER_MODER 2.48 1 8.7113 0.0031770 **

## BMI_MODER 3.49 1 12.2886 0.0004597 ***

## gest 2.86 3 3.3509 0.0181984 *

## Birth_type 0.02 1 0.0600 0.8064580

## Residuals 1435.82 5050

## ---

## Signif. codes: 0 '***' 0.001 '**' 0.01 '*' 0.05 '.' 0.1 ' ' 1

##

## $`Inlabor-prelabor`

## Anova Table (Type III tests)

##

## Response: m_vaginalt[[bio]]

## Sum Sq Df F value Pr(>F)

## (Intercept) 252.972 1 905.4211 < 2.2e-16 ***

## AGE_AT_COLLECTION 3.082 1 11.0292 0.0009261 ***

## ALDER_MODER 0.865 1 3.0949 0.0788148 .

## BMI_MODER 0.010 1 0.0341 0.8535713

## gest 2.018 3 2.4081 0.0656685 .

## Birth_type 0.241 1 0.8640 0.3528218

## Residuals 310.410 1111

## ---

## Signif. codes: 0 '***' 0.001 '**' 0.01 '*' 0.05 '.' 0.1 ' ' 1

##

## $`Pairwise test`

## contrast gest estimate SE df t.ratio p.value

## (In-labor) - (Pre-labor) 37 0.04831 0.1105 5480 0.437 1.0000

## (In-labor) - Vaginal 37 0.07782 0.1029 5480 0.756 1.0000

## (Pre-labor) - Vaginal 37 0.02951 0.0663 5480 0.445 1.0000

## (In-labor) - (Pre-labor) 38 0.07276 0.0742 5480 0.980 1.0000

## (In-labor) - Vaginal 38 0.07980 0.0714 5480 1.118 1.0000

## (Pre-labor) - Vaginal 38 0.00704 0.0393 5480 0.179 1.0000

## (In-labor) - (Pre-labor) 39 0.03658 0.0678 5480 0.540 1.0000

## (In-labor) - Vaginal 39 0.00915 0.0612 5480 0.150 1.0000

## (Pre-labor) - Vaginal 39 -0.02743 0.0381 5480 -0.720 1.0000

## (In-labor) - (Pre-labor) 40+ 0.03538 0.0930 5480 0.381 1.0000

## (In-labor) - Vaginal 40+ 0.04604 0.0347 5480 1.326 1.0000

## (Pre-labor) - Vaginal 40+ 0.01066 0.0875 5480 0.122 1.0000

##

## P value adjustment: holm method for 12 tests

gest_type(bio="logEGF")

## $`Vaginal-inlabor`

## Anova Table (Type III tests)

##

## Response: m_planlagt[[bio]]

## Sum Sq Df F value Pr(>F)

## (Intercept) 423.52 1 1976.5601 < 2.2e-16 ***

## AGE_AT_COLLECTION 2.12 1 9.8833 0.001678 **

## ALDER_MODER 1.49 1 6.9459 0.008428 **

## BMI_MODER 1.14 1 5.3293 0.021012 *

## gest 0.32 3 0.5019 0.680988

## Birth_type 0.00 1 0.0062 0.937271

## Residuals 1029.56 4805

## ---

## Signif. codes: 0 '***' 0.001 '**' 0.01 '*' 0.05 '.' 0.1 ' ' 1

##

## $`Vaginal-prelabor`

## Anova Table (Type III tests)

##

## Response: m_under[[bio]]

## Sum Sq Df F value Pr(>F)

## (Intercept) 470.97 1 2246.9573 < 2.2e-16 ***

## AGE_AT_COLLECTION 1.94 1 9.2759 0.002334 **

## ALDER_MODER 0.61 1 2.9144 0.087854 .

## BMI_MODER 1.02 1 4.8799 0.027215 *

## gest 0.44 3 0.7060 0.548310

## Birth_type 1.15 1 5.5076 0.018972 *

## Residuals 1058.49 5050

## ---

## Signif. codes: 0 '***' 0.001 '**' 0.01 '*' 0.05 '.' 0.1 ' ' 1

##

## $`Inlabor-prelabor`

## Anova Table (Type III tests)

##

## Response: m_vaginalt[[bio]]

## Sum Sq Df F value Pr(>F)

## (Intercept) 98.515 1 513.2996 <2e-16 ***

## AGE_AT_COLLECTION 0.105 1 0.5445 0.4607

## ALDER_MODER 0.115 1 0.6015 0.4382

## BMI_MODER 0.024 1 0.1238 0.7250

## gest 1.200 3 2.0837 0.1007

## Birth_type 0.112 1 0.5815 0.4459

## Residuals 213.229 1111

## ---

## Signif. codes: 0 '***' 0.001 '**' 0.01 '*' 0.05 '.' 0.1 ' ' 1

##

## $`Pairwise test`

## contrast gest estimate SE df t.ratio p.value

## (In-labor) - (Pre-labor) 37 -0.07683 0.0947 5480 -0.811 1.0000

## (In-labor) - Vaginal 37 0.03094 0.0883 5480 0.351 1.0000

## (Pre-labor) - Vaginal 37 0.10777 0.0568 5480 1.897 0.6943

## (In-labor) - (Pre-labor) 38 0.03031 0.0637 5480 0.476 1.0000

## (In-labor) - Vaginal 38 0.08959 0.0612 5480 1.464 1.0000

## (Pre-labor) - Vaginal 38 0.05928 0.0337 5480 1.761 0.8613

## (In-labor) - (Pre-labor) 39 -0.01416 0.0581 5480 -0.244 1.0000

## (In-labor) - Vaginal 39 0.00905 0.0524 5480 0.173 1.0000

## (Pre-labor) - Vaginal 39 0.02321 0.0327 5480 0.710 1.0000

## (In-labor) - (Pre-labor) 40+ -0.08952 0.0797 5480 -1.123 1.0000

## (In-labor) - Vaginal 40+ -0.03126 0.0298 5480 -1.050 1.0000

## (Pre-labor) - Vaginal 40+ 0.05827 0.0750 5480 0.777 1.0000

##

## P value adjustment: holm method for 12 tests

gest_type(bio="logVEGF")

## $`Vaginal-inlabor`

## Anova Table (Type III tests)

##

## Response: m_planlagt[[bio]]

## Sum Sq Df F value Pr(>F)

## (Intercept) 738.89 1 3819.3018 < 2.2e-16 ***

## AGE_AT_COLLECTION 33.04 1 170.7599 < 2.2e-16 ***

## ALDER_MODER 0.92 1 4.7618 0.029146 *

## BMI_MODER 1.34 1 6.9382 0.008464 **

## gest 0.49 3 0.8394 0.472061

## Birth_type 0.48 1 2.4955 0.114236

## Residuals 929.58 4805

## ---

## Signif. codes: 0 '***' 0.001 '**' 0.01 '*' 0.05 '.' 0.1 ' ' 1

##

## $`Vaginal-prelabor`

## Anova Table (Type III tests)

##

## Response: m_under[[bio]]

## Sum Sq Df F value Pr(>F)

## (Intercept) 826.29 1 4331.6908 < 2.2e-16 ***

## AGE_AT_COLLECTION 37.07 1 194.3453 < 2.2e-16 ***

## ALDER_MODER 0.56 1 2.9274 0.0871509 .

## BMI_MODER 0.99 1 5.2109 0.0224870 *

## gest 0.80 3 1.3911 0.2434799

## Birth_type 2.75 1 14.4039 0.0001492 ***

## Residuals 963.31 5050

## ---

## Signif. codes: 0 '***' 0.001 '**' 0.01 '*' 0.05 '.' 0.1 ' ' 1

##

## $`Inlabor-prelabor`

## Anova Table (Type III tests)

##

## Response: m_vaginalt[[bio]]

## Sum Sq Df F value Pr(>F)

## (Intercept) 173.871 1 900.6149 < 2.2e-16 ***

## AGE_AT_COLLECTION 9.415 1 48.7663 4.954e-12 ***

## ALDER_MODER 0.024 1 0.1227 0.7262

## BMI_MODER 0.033 1 0.1724 0.6780

## gest 1.183 3 2.0424 0.1062

## Birth_type 0.173 1 0.8960 0.3441

## Residuals 214.488 1111

## ---

## Signif. codes: 0 '***' 0.001 '**' 0.01 '*' 0.05 '.' 0.1 ' ' 1

##

## $`Pairwise test`

## contrast gest estimate SE df t.ratio p.value

## (In-labor) - (Pre-labor) 37 -0.0224 0.0907 5480 -0.247 1.0000

## (In-labor) - Vaginal 37 0.0624 0.0845 5480 0.738 1.0000

## (Pre-labor) - Vaginal 37 0.0848 0.0544 5480 1.559 1.0000

## (In-labor) - (Pre-labor) 38 -0.0403 0.0609 5480 -0.661 1.0000

## (In-labor) - Vaginal 38 0.0681 0.0586 5480 1.163 1.0000

## (Pre-labor) - Vaginal 38 0.1084 0.0322 5480 3.364 0.0093

## (In-labor) - (Pre-labor) 39 -0.0226 0.0556 5480 -0.407 1.0000

## (In-labor) - Vaginal 39 0.0138 0.0502 5480 0.275 1.0000

## (Pre-labor) - Vaginal 39 0.0364 0.0313 5480 1.165 1.0000

## (In-labor) - (Pre-labor) 40+ -0.0925 0.0763 5480 -1.213 1.0000

## (In-labor) - Vaginal 40+ 0.0301 0.0285 5480 1.055 1.0000

## (Pre-labor) - Vaginal 40+ 0.1226 0.0718 5480 1.708 0.9653

##

## P value adjustment: holm method for 12 tests

gest_type(bio="logS100B")

## $`Vaginal-inlabor`

## Anova Table (Type III tests)

##

## Response: m_planlagt[[bio]]

## Sum Sq Df F value Pr(>F)

## (Intercept) 1425.67 1 4729.2707 <2e-16 ***

## AGE_AT_COLLECTION 0.58 1 1.9196 0.1660

## ALDER_MODER 0.64 1 2.1188 0.1456

## BMI_MODER 0.00 1 0.0029 0.9571

## gest 1.52 3 1.6848 0.1680

## Birth_type 0.79 1 2.6094 0.1063

## Residuals 1448.50 4805

## ---

## Signif. codes: 0 '***' 0.001 '**' 0.01 '*' 0.05 '.' 0.1 ' ' 1

##

## $`Vaginal-prelabor`

## Anova Table (Type III tests)

##

## Response: m_under[[bio]]

## Sum Sq Df F value Pr(>F)

## (Intercept) 1543.15 1 5070.1961 < 2e-16 ***

## AGE_AT_COLLECTION 0.38 1 1.2324 0.26699

## ALDER_MODER 0.01 1 0.0216 0.88328

## BMI_MODER 0.00 1 0.0134 0.90796

## gest 1.46 3 1.6045 0.18613

## Birth_type 1.11 1 3.6308 0.05678 .

## Residuals 1537.01 5050

## ---

## Signif. codes: 0 '***' 0.001 '**' 0.01 '*' 0.05 '.' 0.1 ' ' 1

##

## $`Inlabor-prelabor`

## Anova Table (Type III tests)

##

## Response: m_vaginalt[[bio]]

## Sum Sq Df F value Pr(>F)

## (Intercept) 328.19 1 1023.1729 < 2e-16 ***

## AGE_AT_COLLECTION 1.31 1 4.0729 0.04382 *

## ALDER_MODER 0.00 1 0.0089 0.92491

## BMI_MODER 0.00 1 0.0048 0.94480

## gest 1.50 3 1.5553 0.19861

## Birth_type 0.23 1 0.7172 0.39723

## Residuals 356.37 1111

## ---

## Signif. codes: 0 '***' 0.001 '**' 0.01 '*' 0.05 '.' 0.1 ' ' 1

##

## $`Pairwise test`

## contrast gest estimate SE df t.ratio p.value

## (In-labor) - (Pre-labor) 37 0.03464 0.1141 5480 0.303 1.0000

## (In-labor) - Vaginal 37 0.16086 0.1064 5480 1.512 1.0000

## (Pre-labor) - Vaginal 37 0.12622 0.0685 5480 1.844 0.7833

## (In-labor) - (Pre-labor) 38 -0.01127 0.0767 5480 -0.147 1.0000

## (In-labor) - Vaginal 38 0.04693 0.0737 5480 0.636 1.0000

## (Pre-labor) - Vaginal 38 0.05820 0.0406 5480 1.435 1.0000

## (In-labor) - (Pre-labor) 39 -0.02190 0.0700 5480 -0.313 1.0000

## (In-labor) - Vaginal 39 -0.02071 0.0632 5480 -0.328 1.0000

## (Pre-labor) - Vaginal 39 0.00119 0.0394 5480 0.030 1.0000

## (In-labor) - (Pre-labor) 40+ -0.11235 0.0961 5480 -1.170 1.0000

## (In-labor) - Vaginal 40+ 0.05132 0.0359 5480 1.431 1.0000

## (Pre-labor) - Vaginal 40+ 0.16367 0.0904 5480 1.811 0.7833

##

## P value adjustment: holm method for 12 tests

gest_type(bio="logBDNF")

## $`Vaginal-inlabor`

## Anova Table (Type III tests)

##

## Response: m_planlagt[[bio]]

## Sum Sq Df F value Pr(>F)

## (Intercept) 1613.10 1 2938.7633 < 2.2e-16 ***

## AGE_AT_COLLECTION 3.70 1 6.7366 0.0094741 **

## ALDER_MODER 6.56 1 11.9449 0.0005527 ***

## BMI_MODER 7.24 1 13.1976 0.0002832 ***

## gest 1.74 3 1.0550 0.3669748

## Birth_type 0.53 1 0.9737 0.3237997

## Residuals 2637.48 4805

## ---

## Signif. codes: 0 '***' 0.001 '**' 0.01 '*' 0.05 '.' 0.1 ' ' 1

##

## $`Vaginal-prelabor`

## Anova Table (Type III tests)

##

## Response: m_under[[bio]]

## Sum Sq Df F value Pr(>F)

## (Intercept) 1839.22 1 3402.6157 < 2.2e-16 ***

## AGE_AT_COLLECTION 4.21 1 7.7843 0.0052899 **

## ALDER_MODER 6.93 1 12.8282 0.0003446 ***

## BMI_MODER 6.02 1 11.1313 0.0008549 ***

## gest 1.16 3 0.7166 0.5419259

## Birth_type 1.11 1 2.0595 0.1513243

## Residuals 2729.68 5050

## ---

## Signif. codes: 0 '***' 0.001 '**' 0.01 '*' 0.05 '.' 0.1 ' ' 1

##

## $`Inlabor-prelabor`

## Anova Table (Type III tests)

##

## Response: m_vaginalt[[bio]]

## Sum Sq Df F value Pr(>F)

## (Intercept) 375.80 1 679.8308 < 2e-16 ***

## AGE_AT_COLLECTION 0.00 1 0.0036 0.95228

## ALDER_MODER 2.11 1 3.8257 0.05072 .

## BMI_MODER 0.60 1 1.0868 0.29741

## gest 0.76 3 0.4590 0.71101

## Birth_type 1.90 1 3.4340 0.06413 .

## Residuals 614.14 1111

## ---

## Signif. codes: 0 '***' 0.001 '**' 0.01 '*' 0.05 '.' 0.1 ' ' 1

##

## $`Pairwise test`

## contrast gest estimate SE df t.ratio p.value

## (In-labor) - (Pre-labor) 37 -0.2275 0.1527 5480 -1.490 1.0000

## (In-labor) - Vaginal 37 -0.1529 0.1423 5480 -1.075 1.0000

## (Pre-labor) - Vaginal 37 0.0746 0.0916 5480 0.814 1.0000

## (In-labor) - (Pre-labor) 38 0.0343 0.1026 5480 0.334 1.0000

## (In-labor) - Vaginal 38 0.0603 0.0987 5480 0.611 1.0000

## (Pre-labor) - Vaginal 38 0.0260 0.0543 5480 0.480 1.0000

## (In-labor) - (Pre-labor) 39 -0.0772 0.0937 5480 -0.824 1.0000

## (In-labor) - Vaginal 39 -0.0375 0.0845 5480 -0.443 1.0000

## (Pre-labor) - Vaginal 39 0.0397 0.0527 5480 0.753 1.0000

## (In-labor) - (Pre-labor) 40+ -0.2377 0.1285 5480 -1.850 0.7732

## (In-labor) - Vaginal 40+ -0.0470 0.0480 5480 -0.979 1.0000

## (Pre-labor) - Vaginal 40+ 0.1907 0.1209 5480 1.577 1.0000

##

## P value adjustment: holm method for 12 tests

gest_type(bio="logNT3")

## $`Vaginal-inlabor`

## Anova Table (Type III tests)

##

## Response: m_planlagt[[bio]]

## Sum Sq Df F value Pr(>F)

## (Intercept) 94.04 1 387.2777 < 2.2e-16 ***

## AGE_AT_COLLECTION 3.97 1 16.3379 5.382e-05 ***

## ALDER_MODER 1.88 1 7.7505 0.005391 **

## BMI_MODER 0.80 1 3.3082 0.068997 .

## gest 1.08 3 1.4888 0.215459

## Birth_type 0.06 1 0.2607 0.609673

## Residuals 1166.73 4805

## ---

## Signif. codes: 0 '***' 0.001 '**' 0.01 '*' 0.05 '.' 0.1 ' ' 1

##

## $`Vaginal-prelabor`

## Anova Table (Type III tests)

##

## Response: m_under[[bio]]

## Sum Sq Df F value Pr(>F)

## (Intercept) 101.82 1 429.9260 < 2.2e-16 ***

## AGE_AT_COLLECTION 3.29 1 13.8903 0.0001959 ***

## ALDER_MODER 1.33 1 5.6228 0.0177659 *

## BMI_MODER 0.90 1 3.7982 0.0513633 .

## gest 0.98 3 1.3752 0.2483558

## Birth_type 0.11 1 0.4523 0.5012743

## Residuals 1196.03 5050

## ---

## Signif. codes: 0 '***' 0.001 '**' 0.01 '*' 0.05 '.' 0.1 ' ' 1

##

## $`Inlabor-prelabor`

## Anova Table (Type III tests)

##

## Response: m_vaginalt[[bio]]

## Sum Sq Df F value Pr(>F)

## (Intercept) 16.580 1 75.2178 <2e-16 ***

## AGE_AT_COLLECTION 0.030 1 0.1360 0.7124

## ALDER_MODER 0.082 1 0.3704 0.5429

## BMI_MODER 0.153 1 0.6939 0.4050

## gest 0.827 3 1.2500 0.2903

## Birth_type 0.018 1 0.0833 0.7729

## Residuals 244.900 1111

## ---

## Signif. codes: 0 '***' 0.001 '**' 0.01 '*' 0.05 '.' 0.1 ' ' 1

##

## $`Pairwise test`

## contrast gest estimate SE df t.ratio p.value

## (In-labor) - (Pre-labor) 37 -0.10601 0.1008 5480 -1.051 1.0000

## (In-labor) - Vaginal 37 -0.05427 0.0940 5480 -0.577 1.0000

## (Pre-labor) - Vaginal 37 0.05174 0.0605 5480 0.855 1.0000

## (In-labor) - (Pre-labor) 38 0.02508 0.0678 5480 0.370 1.0000

## (In-labor) - Vaginal 38 0.06649 0.0652 5480 1.020 1.0000

## (Pre-labor) - Vaginal 38 0.04141 0.0358 5480 1.155 1.0000

## (In-labor) - (Pre-labor) 39 0.04032 0.0619 5480 0.652 1.0000

## (In-labor) - Vaginal 39 0.01550 0.0558 5480 0.278 1.0000

## (Pre-labor) - Vaginal 39 -0.02482 0.0348 5480 -0.713 1.0000

## (In-labor) - (Pre-labor) 40+ -0.03916 0.0849 5480 -0.461 1.0000

## (In-labor) - Vaginal 40+ 0.00632 0.0317 5480 0.199 1.0000

## (Pre-labor) - Vaginal 40+ 0.04549 0.0798 5480 0.570 1.0000

##

## P value adjustment: holm method for 12 tests

S4.4 Testing gender

Figure 2 in the paper

Splitting the dataset in boys and girls to get an overall p-value for each of the genders

Testing

koen_type(bio="logCRP")

## $Boys

## Anova Table (Type III tests)

##

## Response: boys[[bio]]

## Sum Sq Df F value Pr(>F)

## (Intercept) 22.91 1 41.6999 1.241e-10 ***

## AGE_AT_COLLECTION 9.89 1 17.9998 2.278e-05 ***

## ALDER_MODER 0.53 1 0.9643 0.3262

## BMI_MODER 0.46 1 0.8314 0.3620

## Birth_type 74.29 2 67.5948 < 2.2e-16 ***

## Residuals 1613.89 2937

## ---

## Signif. codes: 0 '***' 0.001 '**' 0.01 '*' 0.05 '.' 0.1 ' ' 1

##

## $Girls

## Anova Table (Type III tests)

##

## Response: girls[[bio]]

## Sum Sq Df F value Pr(>F)

## (Intercept) 19.73 1 37.6512 9.781e-10 ***

## AGE_AT_COLLECTION 9.47 1 18.0609 2.216e-05 ***

## ALDER_MODER 1.30 1 2.4789 0.1155

## BMI_MODER 0.83 1 1.5761 0.2094

## Birth_type 96.28 2 91.8514 < 2.2e-16 ***

## Residuals 1334.43 2546

## ---

## Signif. codes: 0 '***' 0.001 '**' 0.01 '*' 0.05 '.' 0.1 ' ' 1

##

## $`Pairwise test`

## contrast Birth_type estimate SE df t.ratio p.value

## K - M In-labor -0.0919 0.0717 5486 -1.282 0.2000

## K - M Pre-labor -0.2633 0.0563 5486 -4.679 <.0001

## K - M Vaginal -0.1559 0.0222 5486 -7.019 <.0001

##

## P value adjustment: holm method for 3 tests

koen_type(bio="logMCP1")

## $Boys

## Anova Table (Type III tests)

##

## Response: boys[[bio]]

## Sum Sq Df F value Pr(>F)

## (Intercept) 1100.61 1 5795.8166 <2e-16 ***

## AGE_AT_COLLECTION 18.76 1 98.8034 <2e-16 ***

## ALDER_MODER 0.09 1 0.4738 0.4913

## BMI_MODER 0.05 1 0.2639 0.6075

## Birth_type 15.75 2 41.4749 <2e-16 ***

## Residuals 557.73 2937

## ---

## Signif. codes: 0 '***' 0.001 '**' 0.01 '*' 0.05 '.' 0.1 ' ' 1

##

## $Girls

## Anova Table (Type III tests)

##

## Response: girls[[bio]]

## Sum Sq Df F value Pr(>F)

## (Intercept) 912.35 1 5292.3321 < 2.2e-16 ***

## AGE_AT_COLLECTION 22.79 1 132.2126 < 2.2e-16 ***

## ALDER_MODER 0.52 1 3.0377 0.08147 .

## BMI_MODER 0.01 1 0.0293 0.86421

## Birth_type 12.00 2 34.8170 1.208e-15 ***

## Residuals 438.91 2546

## ---

## Signif. codes: 0 '***' 0.001 '**' 0.01 '*' 0.05 '.' 0.1 ' ' 1

##

## $`Pairwise test`

## contrast Birth_type estimate SE df t.ratio p.value

## K - M In-labor -0.0548 0.0417 5486 -1.314 0.1889

## K - M Pre-labor -0.0629 0.0327 5486 -1.922 0.1093

## K - M Vaginal -0.0810 0.0129 5486 -6.267 <.0001

##

## P value adjustment: holm method for 3 tests

koen_type(bio="logIL18")

## $Boys

## Anova Table (Type III tests)

##

## Response: boys[[bio]]

## Sum Sq Df F value Pr(>F)

## (Intercept) 322.57 1 1771.9647 < 2.2e-16 ***

## AGE_AT_COLLECTION 0.06 1 0.3513 0.553449

## ALDER_MODER 0.03 1 0.1508 0.697787

## BMI_MODER 1.00 1 5.4778 0.019326 *

## Birth_type 1.96 2 5.3810 0.004649 **

## Residuals 534.66 2937

## ---

## Signif. codes: 0 '***' 0.001 '**' 0.01 '*' 0.05 '.' 0.1 ' ' 1

##

## $Girls

## Anova Table (Type III tests)

##

## Response: girls[[bio]]

## Sum Sq Df F value Pr(>F)

## (Intercept) 253.83 1 1441.8939 < 2e-16 ***

## AGE_AT_COLLECTION 0.04 1 0.2206 0.63863

## ALDER_MODER 0.32 1 1.8213 0.17727

## BMI_MODER 0.17 1 0.9540 0.32880

## Birth_type 1.16 2 3.3084 0.03673 *

## Residuals 448.19 2546

## ---

## Signif. codes: 0 '***' 0.001 '**' 0.01 '*' 0.05 '.' 0.1 ' ' 1

##

## $`Pairwise test`

## contrast Birth_type estimate SE df t.ratio p.value

## K - M In-labor -0.0273 0.0414 5486 -0.659 1.0000

## K - M Pre-labor 0.0045 0.0325 5486 0.139 1.0000

## K - M Vaginal -0.0149 0.0128 5486 -1.159 0.7395

##

## P value adjustment: holm method for 3 tests

koen_type(bio="logHSP70")

## $Boys

## Anova Table (Type III tests)

##

## Response: boys[[bio]]

## Sum Sq Df F value Pr(>F)

## (Intercept) 4815.9 1 54164.7289 < 2.2e-16 ***

## AGE_AT_COLLECTION 0.2 1 1.9955 0.157877

## ALDER_MODER 0.0 1 0.4794 0.488752

## BMI_MODER 0.2 1 2.5317 0.111689

## Birth_type 1.2 2 6.9672 0.000958 ***

## Residuals 261.1 2937

## ---

## Signif. codes: 0 '***' 0.001 '**' 0.01 '*' 0.05 '.' 0.1 ' ' 1

##

## $Girls

## Anova Table (Type III tests)

##

## Response: girls[[bio]]

## Sum Sq Df F value Pr(>F)

## (Intercept) 3799.2 1 45623.6212 < 2.2e-16 ***

## AGE_AT_COLLECTION 0.1 1 0.9314 0.3346

## ALDER_MODER 0.0 1 0.2180 0.6406

## BMI_MODER 0.2 1 2.4756 0.1158

## Birth_type 1.8 2 10.9254 1.885e-05 ***

## Residuals 212.0 2546

## ---

## Signif. codes: 0 '***' 0.001 '**' 0.01 '*' 0.05 '.' 0.1 ' ' 1

##

## $`Pairwise test`

## contrast Birth_type estimate SE df t.ratio p.value

## K - M In-labor 0.00848 0.0287 5486 0.295 0.9902

## K - M Pre-labor -0.01538 0.0225 5486 -0.682 0.9902

## K - M Vaginal 0.01435 0.0089 5486 1.612 0.3210

##

## P value adjustment: holm method for 3 tests

koen_type(bio="logSTNF_RI")

## $Boys

## Anova Table (Type III tests)

##

## Response: boys[[bio]]

## Sum Sq Df F value Pr(>F)

## (Intercept) 807.19 1 2925.6746 < 2.2e-16 ***

## AGE_AT_COLLECTION 13.85 1 50.1927 1.737e-12 ***

## ALDER_MODER 1.40 1 5.0844 0.02421 *

## BMI_MODER 0.75 1 2.7259 0.09884 .

## Birth_type 1.55 2 2.8146 0.06009 .

## Residuals 810.32 2937

## ---

## Signif. codes: 0 '***' 0.001 '**' 0.01 '*' 0.05 '.' 0.1 ' ' 1

##

## $Girls

## Anova Table (Type III tests)

##

## Response: girls[[bio]]

## Sum Sq Df F value Pr(>F)

## (Intercept) 697.60 1 2355.7995 < 2.2e-16 ***

## AGE_AT_COLLECTION 19.51 1 65.8909 7.339e-16 ***

## ALDER_MODER 1.89 1 6.3917 0.0115253 *

## BMI_MODER 3.97 1 13.4002 0.0002567 ***

## Birth_type 0.19 2 0.3214 0.7251576

## Residuals 753.93 2546

## ---

## Signif. codes: 0 '***' 0.001 '**' 0.01 '*' 0.05 '.' 0.1 ' ' 1

##

## $`Pairwise test`

## contrast Birth_type estimate SE df t.ratio p.value

## K - M In-labor 0.00211 0.0522 5486 0.040 1.0000

## K - M Pre-labor -0.01149 0.0410 5486 -0.280 1.0000

## K - M Vaginal 0.04519 0.0162 5486 2.791 0.0158

##

## P value adjustment: holm method for 3 tests

koen_type(bio="logEGF")

## $Boys

## Anova Table (Type III tests)

##

## Response: boys[[bio]]

## Sum Sq Df F value Pr(>F)

## (Intercept) 298.72 1 1433.8133 < 2e-16 ***

## AGE_AT_COLLECTION 0.91 1 4.3696 0.03667 *

## ALDER_MODER 0.18 1 0.8512 0.35629

## BMI_MODER 0.31 1 1.4942 0.22167

## Birth_type 0.60 2 1.4375 0.23770

## Residuals 611.90 2937

## ---

## Signif. codes: 0 '***' 0.001 '**' 0.01 '*' 0.05 '.' 0.1 ' ' 1

##

## $Girls

## Anova Table (Type III tests)

##

## Response: girls[[bio]]

## Sum Sq Df F value Pr(>F)

## (Intercept) 270.00 1 1284.1810 < 2e-16 ***

## AGE_AT_COLLECTION 1.14 1 5.4088 0.02011 *

## ALDER_MODER 1.14 1 5.4411 0.01975 *

## BMI_MODER 0.86 1 4.1070 0.04281 *

## Birth_type 1.12 2 2.6577 0.07031 .

## Residuals 535.30 2546

## ---

## Signif. codes: 0 '***' 0.001 '**' 0.01 '*' 0.05 '.' 0.1 ' ' 1

##

## $`Pairwise test`

## contrast Birth_type estimate SE df t.ratio p.value

## K - M In-labor 0.0662 0.0447 5486 1.481 0.1388

## K - M Pre-labor 0.0672 0.0351 5486 1.915 0.1110

## K - M Vaginal 0.0522 0.0139 5486 3.765 0.0005

##

## P value adjustment: holm method for 3 tests

koen_type(bio="logVEGF")

## $Boys

## Anova Table (Type III tests)

##

## Response: boys[[bio]]

## Sum Sq Df F value Pr(>F)

## (Intercept) 528.64 1 2808.3810 < 2.2e-16 ***

## AGE_AT_COLLECTION 18.88 1 100.2929 < 2.2e-16 ***

## ALDER_MODER 0.17 1 0.8917 0.345099

## BMI_MODER 0.31 1 1.6257 0.202401

## Birth_type 2.00 2 5.3071 0.005004 **

## Residuals 552.85 2937

## ---

## Signif. codes: 0 '***' 0.001 '**' 0.01 '*' 0.05 '.' 0.1 ' ' 1

##

## $Girls

## Anova Table (Type III tests)

##

## Response: girls[[bio]]

## Sum Sq Df F value Pr(>F)

## (Intercept) 471.76 1 2435.8837 < 2.2e-16 ***

## AGE_AT_COLLECTION 21.28 1 109.8810 < 2.2e-16 ***

## ALDER_MODER 0.74 1 3.8049 0.0512125 .

## BMI_MODER 0.84 1 4.3145 0.0378897 *

## Birth_type 3.12 2 8.0597 0.0003241 ***

## Residuals 493.09 2546

## ---

## Signif. codes: 0 '***' 0.001 '**' 0.01 '*' 0.05 '.' 0.1 ' ' 1

##

## $`Pairwise test`

## contrast Birth_type estimate SE df t.ratio p.value

## K - M In-labor 0.0888 0.0427 5486 2.081 0.0375

## K - M Pre-labor 0.1008 0.0335 5486 3.007 0.0053

## K - M Vaginal 0.0761 0.0132 5486 5.752 <.0001

##

## P value adjustment: holm method for 3 tests

koen_type(bio="logS100B")

## $Boys

## Anova Table (Type III tests)

##

## Response: boys[[bio]]

## Sum Sq Df F value Pr(>F)

## (Intercept) 1066.40 1 3420.8797 < 2.2e-16 ***

## AGE_AT_COLLECTION 0.20 1 0.6471 0.421226

## ALDER_MODER 0.01 1 0.0186 0.891670

## BMI_MODER 0.00 1 0.0003 0.985815

## Birth_type 3.48 2 5.5818 0.003806 **

## Residuals 915.56 2937

## ---

## Signif. codes: 0 '***' 0.001 '**' 0.01 '*' 0.05 '.' 0.1 ' ' 1

##

## $Girls

## Anova Table (Type III tests)

##

## Response: girls[[bio]]

## Sum Sq Df F value Pr(>F)

## (Intercept) 838.78 1 2821.5004 < 2e-16 ***

## AGE_AT_COLLECTION 0.81 1 2.7151 0.09952 .

## ALDER_MODER 0.40 1 1.3391 0.24730

## BMI_MODER 0.00 1 0.0097 0.92136

## Birth_type 0.76 2 1.2771 0.27904

## Residuals 756.87 2546

## ---

## Signif. codes: 0 '***' 0.001 '**' 0.01 '*' 0.05 '.' 0.1 ' ' 1

##

## $`Pairwise test`

## contrast Birth_type estimate SE df t.ratio p.value

## K - M In-labor -0.0435 0.0540 5486 -0.805 0.8417

## K - M Pre-labor -0.0232 0.0424 5486 -0.546 0.8417

## K - M Vaginal 0.0242 0.0167 5486 1.444 0.4467

##

## P value adjustment: holm method for 3 tests

koen_type(bio="logBDNF")

## $Boys

## Anova Table (Type III tests)

##

## Response: boys[[bio]]

## Sum Sq Df F value Pr(>F)

## (Intercept) 1220.51 1 2206.9416 < 2.2e-16 ***

## AGE_AT_COLLECTION 0.90 1 1.6231 0.202762

## ALDER_MODER 4.97 1 8.9834 0.002747 **

## BMI_MODER 4.13 1 7.4673 0.006321 **

## Birth_type 1.90 2 1.7197 0.179298

## Residuals 1624.26 2937

## ---

## Signif. codes: 0 '***' 0.001 '**' 0.01 '*' 0.05 '.' 0.1 ' ' 1

##

## $Girls

## Anova Table (Type III tests)

##

## Response: girls[[bio]]

## Sum Sq Df F value Pr(>F)

## (Intercept) 1052.36 1 1976.3429 < 2.2e-16 ***

## AGE_AT_COLLECTION 3.73 1 7.0139 0.008138 **

## ALDER_MODER 3.12 1 5.8513 0.015635 *

## BMI_MODER 2.76 1 5.1923 0.022770 *

## Birth_type 0.98 2 0.9225 0.397665

## Residuals 1355.69 2546

## ---

## Signif. codes: 0 '***' 0.001 '**' 0.01 '*' 0.05 '.' 0.1 ' ' 1

##

## $`Pairwise test`

## contrast Birth_type estimate SE df t.ratio p.value

## K - M In-labor 0.1984 0.0721 5486 2.754 0.0118

## K - M Pre-labor 0.1162 0.0566 5486 2.053 0.0401

## K - M Vaginal 0.0733 0.0223 5486 3.282 0.0031

##

## P value adjustment: holm method for 3 tests

koen_type(bio="logNT3")

## $Boys

## Anova Table (Type III tests)

##

## Response: boys[[bio]]

## Sum Sq Df F value Pr(>F)

## (Intercept) 66.90 1 293.4098 < 2e-16 ***

## AGE_AT_COLLECTION 1.28 1 5.6283 0.01774 *

## ALDER_MODER 0.69 1 3.0478 0.08095 .

## BMI_MODER 0.14 1 0.6114 0.43434

## Birth_type 0.12 2 0.2734 0.76078

## Residuals 669.61 2937

## ---

## Signif. codes: 0 '***' 0.001 '**' 0.01 '*' 0.05 '.' 0.1 ' ' 1

##

## $Girls

## Anova Table (Type III tests)

##

## Response: girls[[bio]]

## Sum Sq Df F value Pr(>F)

## (Intercept) 66.59 1 266.8716 < 2.2e-16 ***

## AGE_AT_COLLECTION 2.10 1 8.4045 0.003775 **

## ALDER_MODER 0.90 1 3.6055 0.057703 .

## BMI_MODER 1.04 1 4.1517 0.041696 *

## Birth_type 0.34 2 0.6893 0.502013

## Residuals 635.25 2546

## ---

## Signif. codes: 0 '***' 0.001 '**' 0.01 '*' 0.05 '.' 0.1 ' ' 1

##

## $`Pairwise test`

## contrast Birth_type estimate SE df t.ratio p.value

## K - M In-labor 0.0594 0.0477 5486 1.246 0.6383

## K - M Pre-labor -0.0178 0.0374 5486 -0.476 0.7766

## K - M Vaginal 0.0128 0.0148 5486 0.863 0.7766

##

## P value adjustment: holm method for 3 tests
